# Tactile-Transparent Wearable Sensor for Clinician-Friendly Pulse Wave Velocity Monitoring and Cardiovascular Risk Profiling

**DOI:** 10.1021/acsnano.5c11375

**Published:** 2025-09-05

**Authors:** Senlin Hou, Xiaotong Chen, Dani S. Assi, Yu Feng, Chun-Ka Wong, Jingting Tian, Jian Li, Xiaodong Yu, Binghe Guan, Xiaohu Zhu, Xinge Yu, Xinyue Li, Vellaisamy A. L. Roy, Jiangang Shen, Wen Jung Li

**Affiliations:** † Department of Mechanical Engineering, 53025 City University of Hong Kong, Kowloon 000000, Hong Kong; ‡ School of Chinese Medicine, Li Ka Shing Faculty of Medicine, 25809 The University of Hong Kong, Pok Fu Lam 000000, Hong Kong; § School of Science and Technology, 66386 Hong Kong Metropolitan University, Ho Man Tin 000000, Hong Kong; ∥ Department of Medicine, School of Clinical Medicine, Li Ka Shing Faculty of Medicine, The University of Hong Kong, Pok Fu Lam 000000, Hong Kong; ⊥ Department of Biomedical Engineering, City University of Hong Kong, Kowloon 000000, Hong Kong; # Preventive Treatment Center, Shenzhen Bao’an Authentic TCM Therapy Hospital, Shenzhen 518000, Guangdong Province China; ¶ Ultrasound Medicine Department, Shenzhen Bao’an Authentic TCM Therapy Hospital, Shenzhen 518000, Guangdong Province China; ∇ Department of Data Science, City University of Hong Kong, Kowloon 000000, Hong Kong

**Keywords:** pulse wave velocity, arterial stiffening, wearable sensors, tactile transparent, graphene oxide

## Abstract

Arterial stiffening is an independent risk factor for cardiovascular diseases, particularly affecting organs with low vascular resistance, such as the brain and kidneys. Pulse wave velocity (PWV) is the clinical gold standard for arterial stiffness assessment; however, conventional equipment requires complex setups and trained operators, limiting real-world and point-of-care monitoring. Here, we introduce a tactile-transparent wearable (TTW) sensor that preserves physicians’ tactile pulse palpation abilities while providing quantitative cardiovascular risk assessment by integrating flexible Polydimethylsiloxane (PDMS) electrodes and ultrathin graphene oxide dielectric films. In a clinical study with 20 healthy volunteers (aged 22–60 years), the TTW sensor showed strong agreement with Doppler ultrasound for carotid-radial PWV (Pearson’s *r* = 0.88, *p* < 0.001; mean difference = – 0.084 m/s) and identified that arterial stiffness is significantly correlated with age and BMI (both *p* < 0.001). High-fidelity waveform analysis further provided dynamic vascular risk indices (augmentation index, reflection index, stiffness index) not accessible by Doppler. This TTW sensor democratizes arterial stiffness assessment, providing transformative potential for preventive cardiology and enabling widespread adoption, especially in resource-limited or community healthcare settings.

## Graphical abstract

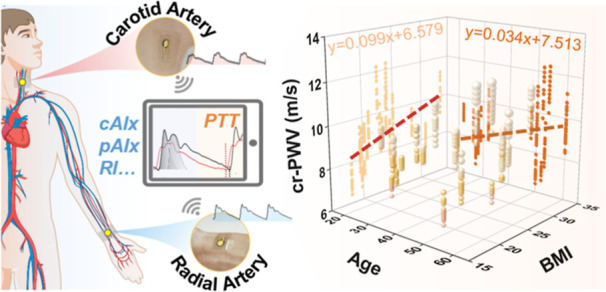

Cardiovascular diseases (CVDs), including coronary heart disease and stroke, remain the leading causes of death worldwide, accounting for an estimated 17.8 million deaths annually—an alarmingly persistent figure over recent decades.^1^ A critical factor in the progression of many CVDs is arterial stiffness, a measure of the elastic properties of the arterial wall. Arterial stiffness significantly affects pulse pressure, blood flow dynamics in peripheral arteries with each heartbeat, and structural changes in the arterial wall, serving as a key indicator of arterial and overall cardiovascular health.^2^ Pulse wave velocity (PWV) is widely recognized as the gold standard for arterial stiffness assessment.^3,4^ It foreshadows adverse cardiovascular events and highlights its utility in the early identification of high-risk individuals.^5,6^ In addition, accurate pulse wave measurements are essential for assessing cardiovascular health status, as they provide crucial information about arterial stiffness, blood pressure, and wave reflection, which are fundamental indicators of vascular function and overall cardiovascular risk.^7–11^ These approaches enable timely interventions, thereby reducing morbidity and mortality associated with CVDs and promoting long-term cardiovascular health.

PWV is calculated using the pulse transit time along the arterial tree and the distance interval between the two locations.^12^ In clinical settings, while magnetic resonance imaging (MRI)^13–15^ and Doppler ultrasound^16–18^ provide high-accuracy PWV evaluation, their high cost and operational complexity limit routine use. Tonometry, though noninvasive, demands precise sensor placement and operator expertise.^17,19^ Cuff-based measurement schemes have emerged as an alternative, providing simplified measurement processes and reduced reliance on operator expertise, but induce discomfort during prolonged use caused by repeated cuff inflation and deflation.^20^ As technology advances, wearable devices have garnered attention for providing portability, continuous monitoring, and real-time feedback in cardiovascular function detection. Wearable devices based on photoplethysmography (PPG), ultrasound, and flexible pressure sensors have been extensively investigated. Among these, PPG is one of the most commonly used methods due to its simplicity and low cost, however, it faces challenges in motion artifact, signal aliasing between arteriovenous signals, and insufficient penetration depth.^12,21,22^ Flexible ultrasound patches are promising candidates for PWV monitoring due to their high penetration depth and strong sensing capability for hemodynamic parameters.^23–25^ However, these patches necessitate complex postprocessing, which can complicate their widespread adoption. Recent developments in flexible electronics have enabled the development of wearable devices for continuous pulse wave monitoring using flexible sensors such as capacitive,^5,26^ piezoresistive,^27–29^ and piezoelectric sensors.^10,21,30–32^ Among these sensing mechanisms, capacitive sensors stand out due to their simple working principle, good pressure sensitivity, fast response time, and compact circuit design.

Here, we develop a novel capacitive-based tactile-transparent wearable (TTW) sensor addressing these limitations, designed to collect high-fidelity waveforms from superficial arteries for cardiovascular assessments accurately. The TTW sensor incorporates flexible PDMS electrodes and thin-paper graphene oxide films for the dielectric layer, achieving outstanding linearity, sensitivity, and durability. The extremely thin sensor thickness enables physicians to palpate radial artery pulses through the device while capturing precise waveforms. By synchronizing proximal-distal arterial pulses (e.g., carotid-radial), the system achieves Doppler-level accuracy (Pearson’s *r* = 0.88, *p* < 0.001) in 20 healthy volunteers (22–60 years), eliminating operator dependency inherent to tonometry. Additionally, the system was further utilized to track real-time arterial stiffness indices under physical activity (cycling, reflection index Δ = 37%, stiffness index Δ = 1.33 m/s). Compared with commercial PWV monitoring devices, the TTW sensor provides a simpler, faster, and more clinician-friendly measurement process, democratizing vascular screening in resource-limited settings. This work introduces a clinician-centric wearable platform for continuous PWV monitoring, expanded means of monitoring arterial stiffness and other vascular diseases.

## Results and Discussion

### Clinical Significance and Design Concept of the TTW Sensor

Among distinct arterial measurement pathways, carotid-femoral pulse wave velocity (cf-PWV) is recognized as the gold standard for evaluating atherosclerosis in major arteries.^33,34^ In peripheral arteries, PWV serves as an essential clinical indicator of conditions such as vascular-muscular insufficiency in the elderly.^17^ Current PWV measurement approaches, including MRI, Doppler ultrasound, tonometry, and cuff-based systems, suffer from limitations such as high cost, operational complexity, operator dependency, or discomfort during prolonged use, hindering their adoption in routine clinical practice and continuous monitoring.

For these challenges, the TTW sensor system combines tactile-transparent sensors with a high-speed data acquisition circuit, introducing a novel solution for the efficient and accurate acquisition of PWV signals from superficial arteries such as carotid-femoral, carotid-radial, and brachial–radial pathways. The TTW sensor’s portability and ability to conform seamlessly to the skin enable continuous, wearable PWV monitoring. By adhering to the skin surface over target arteries (e.g., carotid, radial), physicians can palpate and stabilize the sensor via fingertip pressure, enabling real-time capture of high-fidelity pulse waveforms and calculation of pulse transit time (PTT) and risk markers (e.g., augmentation index, reflection index, and stiffness index) to reflect the cardiovascular status (Figure 1A). Compared to ultrasound or cuff-based methods, the TTW sensor system provides a noninvasive, portable, and continuous monitoring solution, providing a practical pathway for vascular health management in both clinical and everyday settings.

**Figure 1:**
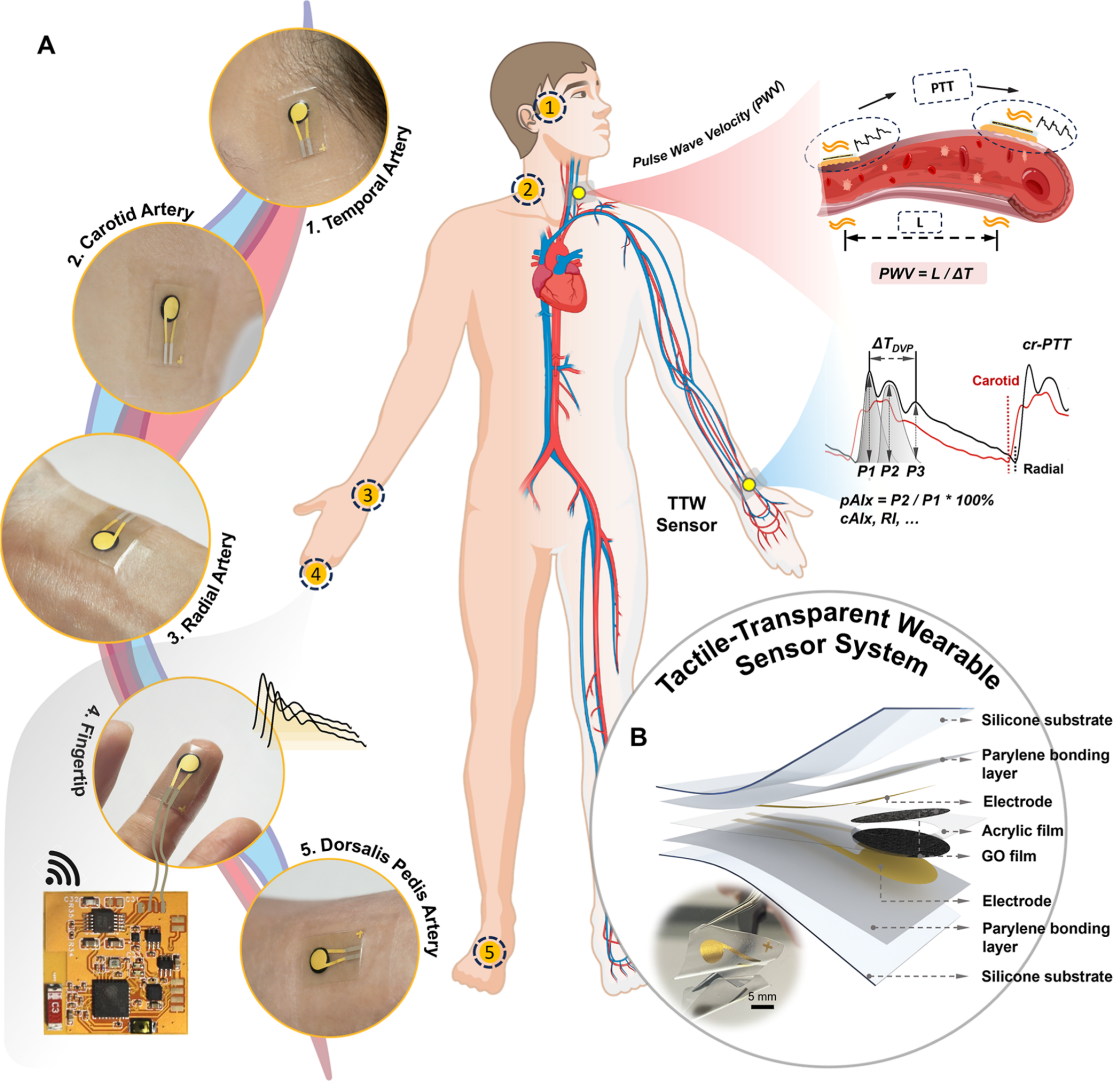
Concept and layouts of the tactile-transparent wearable sensor. (A) Schematic diagram of pulse wave velocity (PWV) monitoring and cardiovascular risk analysis. The PWV is calculated from the time difference and distance between the two sensor units. (B) Explosive view of the TTW sensor and optical image of the PDMS electrode.

### Working Principle and Structural Design of the TTW Sensor

The TTW sensor works on the capacitive sensing principle, converting arterial pulsations into electrical signals. When blood is ejected from the left ventricle into the aorta and peripheral arteries, vascular expansion induces localized tissue deformation. The mechanical pressure alters the distance between the electrodes and compresses the dielectric layer, generating a capacitive response recorded as a continuous pulse.

As illustrated in Figure 1B, the sensor is primarily composed of three layers: the upper and bottom layers consist of flexible PDMS electrodes, each coated with a parylene bonding layer. To address the insufficient interfacial adhesion between Au and PDMS, a layer of parylene C is plated on the reverse side of the PDMS film, acting as a bonding interface. The fabrication details are shown in Methods and Figure S1, Supporting Information. This parylene layer not only provides a stable surface for the Au sputtered on top but also provides an acrylic adhesive surface for the electrode, which reduces the difficulty of sensor encapsulation. The dielectric layer consists of a thin film of graphene oxide dried at a low temperature. With compact dimensions (15 × 8 mm) and an ultrathin profile (<150 μm), the sensor achieves high signal-to-noise ratios while minimizing interference with physicians’ tactile feedback during palpation. This design positions the TTW system as an ideal platform for long-term, continuous cardiovascular monitoring.

### Material and Characteristic Analysis of the TTW Sensor

To validate the sensor’s potential for clinical adoption, a comprehensive characterization of its electrical, mechanical, and sensing performance was conducted. The dielectric layer, critical to capacitive sensing performance, was engineered using graphene oxide (GO). As a derivative of graphene, GO exhibits good mechanical, thermal, and electrical properties, making it a suitable dielectric material. GO’s high relative dielectric constant (ε_*r*_ = 2.2 at 50 kHz for 50 °C-dried films, Figure S2 and Text S1, Supporting Information) arises from its oxygen-functionalized group and structural defect, which hinder electron mobility while enhancing polarization under electric fields.^35,36^ Figure 2A shows a schematic structure and equivalent circuit diagram of the TTW sensor cross-section. Two layers of GO film are applied to the upper and bottom electrode surfaces. This design leverages air (ε_0_ = 1) introduced by the air layer between the two GO layers and structural defects in the GO film, as a secondary dielectric, reducing initial capacitance (*C*_0_) while enabling dynamic modulation via pressure-induced contact area (*A*) and air gaps (*d*) changes. Figure 2B,C showcase the SEM images of the cross-section of the TTW sensor, illustrating the surface structure and position of the PDMS electrode and GO film. Two layers of GO film are formed by depositing an aqueous GO dispersion onto the upper and bottom electrode surfaces, followed by baking at different drying temperatures (50 °C, 100 °C, and 150 °C). GO is uniformly dispersed into water, and the color of the solution gradually deepens as the GO concentration increases (Figure 2D). The relationship between thickness, surface profile, and GO concentration is shown in Figures 2E and S3 Supporting Information. The thickness of the GO film increases gradually with the increase of GO concentration and surface roughness. The dielectric constant and dissipation coefficient of the GO film increase with higher drying temperatures, but higher drying temperatures significantly increase the Young’s modulus due to hydrophilicity loss, resulting in the films being brittle and unsuitable for flexible sensor applications (Figures S4 and S5, Supporting Information). The 2 mg/mL GO concentration was chosen. Air trapped between the GO films acts as part of the dielectric layer. When the sensor is subjected to pressure, the air gap decreases, resulting in an increased contact area between the two layers of GO film. Simultaneously, voids within each GO layer are also being pressurized, increasing in capacitance as shown in Figure 3A (For sensing principle and theoretical explanation, refer to Text S2, Supporting Information).

**Figure 2:**
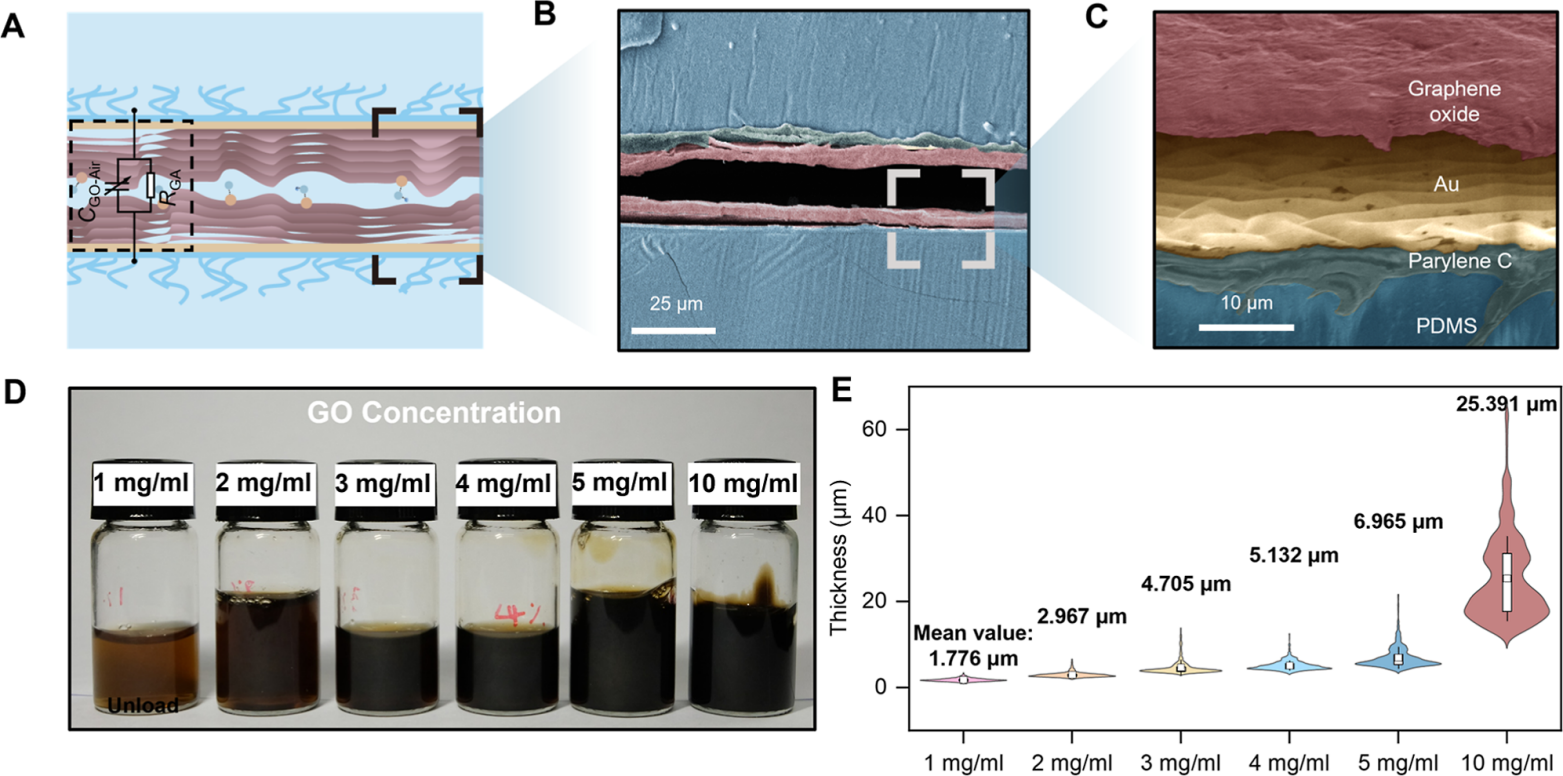
SEM image of the TTW sensor cross-section and dielectric material analysis. (A) Schematic illustration of the TTW sensor and equivalent circuit model. (B,C) SEM images of the cross-section of the TTW sensor. (D) Optical images of aqueous dispersions of GO at different concentrations, from 1 mg/mL to 10 mg/mL. (E) Comparison of the thickness and surface flatness of paper-thin graphene oxide films obtained from GO aqueous dispersions with different concentrations, from 1 mg/mL to 10 mg/mL.

**Figure 3:**
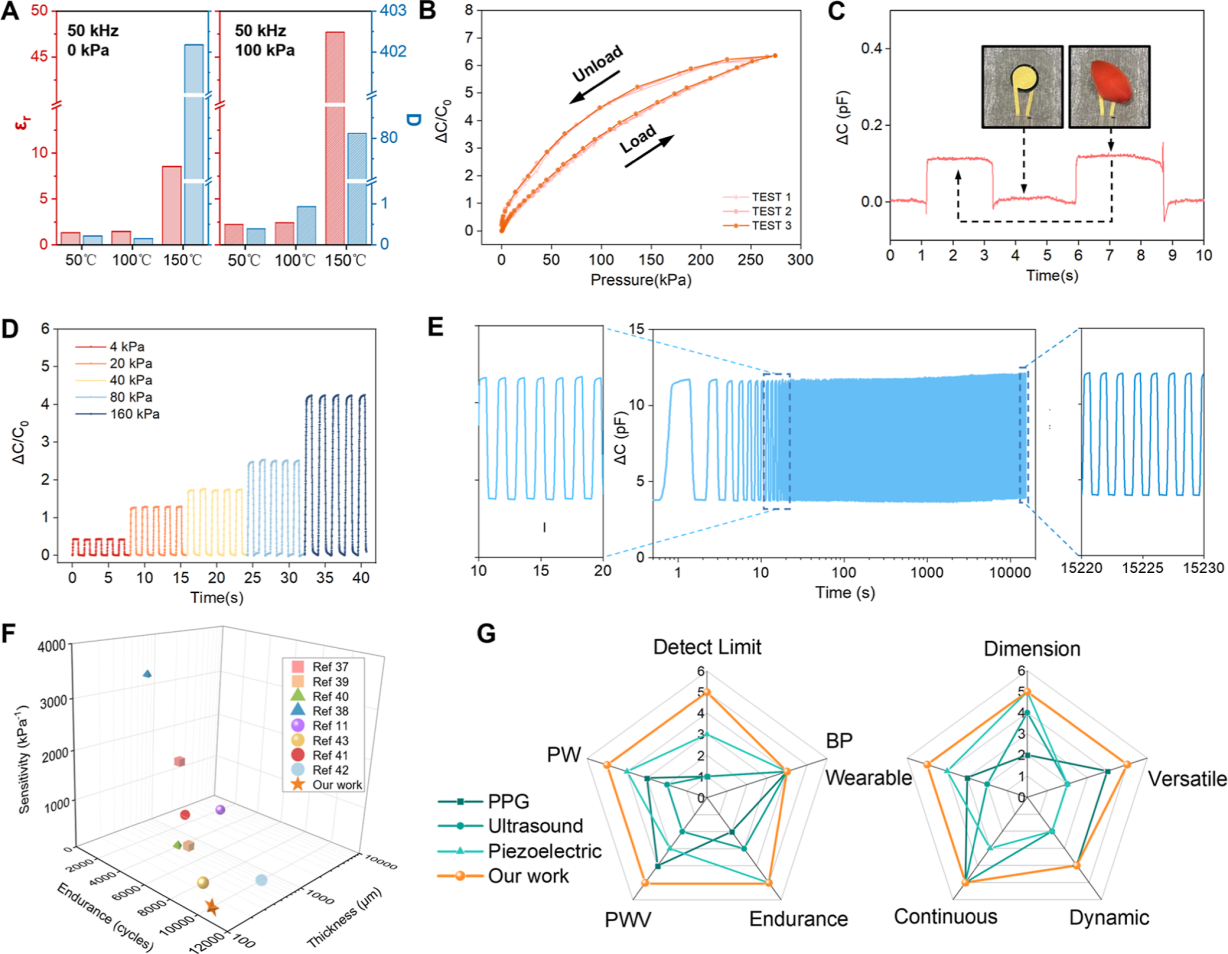
Electrical performance of the TTW sensor. (A) Comparison of different drying temperatures and pressures affected on dielectric spectroscopy of GO film at 50 kHz. (B) Change in the sensor’s capacitance over a pressure range of up to 250 kPa, load and unload. (C) Response to placing and removing of a petal (10 mg) on top of the sensor. (D) Relative change in capacitance of the sensor under repeated pressure of 4, 20, 40, 80, and 160 kPa. (E) The sensor’s working stability was determined after 10,000 cycles from 1 to 20 kPa; the insets show the close-up views at the beginning and end of the test. (F) Thickness, durability, and sensitivity of TTW sensors compared with reported capacitive sensors. (G) Technical comparison between our device and published works utilizing PPG, ultrasound, and piezoelectric for hemodynamic monitoring in terms of ten aspects.

To measure the PWV, the sensor needs to accurately measure the arterial wave profile from the superficial artery. The pressures range from 10 to 100 kPa when the physician’s palpation senses the superficial artery (for details of the pressure test of the physician, refer to Figure S6 and Text S3, Supporting Information). The sensitivity of the TTW sensor was evaluated using a MARK-10 Series 5 Force Gauge (Mark-10 Corp, Copiague, NY) paired with a test stand (ESM303, Mark-10). The probe of the force gauge fully covered the sensor’s monitoring area, enabling precise determination of the applied pressure based on the force exerted and the contact area between the probe and the sensor. Sensitivity measurements were conducted within a pressure range of 0 to 250 kPa, with changes in capacitance recorded continuously using an LCR meter (HIOKI IM3570, Japan) operating at 50 kHz and 1 V. The applied pressure and the corresponding capacitance response were plotted point by point, and the sensitivity was calculated based on the tangent line of the resulting curve (Figures 3B and S7, Supporting Information). The sensor demonstrated a sensitivity of 0.06 kPa^–1^ in the range of 0 to 10 kPa and a sensitivity of 0.03 kPa^–1^ in the range of 10 to 250 kPa. These results confirm the sensor’s excellent linearity across the test range (*R* ^2^ > 0.975). To ensure the reliability of the data, two repetitive tests of the sensor were conducted, proving that the results were consistent. The mechanical vibration signals induced by pulse waves in the superficial peripheral arteries are weak, so the ability of the sensors to perceive weak signals is essential. To determine the limit of detection, a flower petal weighing 10 mg (∼5 Pa) was placed on the sensor and then removed (Figure 3C). The sensor’s capacitance varied significantly (approximately 0.1 pF) in response, demonstrating its capability to perceive weak signals effectively. The sensor was further subjected to a gradient repeatability test in the regular pressing pressure interval, with 5 repetitions of each pressure at 4, 20, 40, 80, and 160 kPa. As shown in Figure 3D, the results demonstrate the sensor’s high-resolution pressure detection ability. In the repeatability and stability tests, the sensor maintained consistent sensitivity after enduring 10,000 pressure cycles from 1 to 20 kPa (Figure 3E). The inset in Figure 3E shows that the amplitude of signals at the beginning and end of the repetitive test displayed nearly identical waveforms, confirming the sensor’s durability and stability.

A systematic comparison with reported flexible capacitive sensors is presented (Figure 3F). Capacitive sensors employing ionic gels or complex three-dimensional microstructures^37–40^ can achieve sensitivities exceeding 3000 kPa^–1^ but often exhibit limited mechanical durability and increased thickness, constraining their applicability for long-term, unobtrusive monitoring. The TTW sensor achieves a balanced performance, combining endurance over 10,000 cycles with an ultrathin profile (∼150 μm), offering advantages in conformal skin attachment and maintaining signal fidelity compared with thicker, millimeter-scale devices.^11,41–43^ These physical properties above underpin the application-level performance shown in Figure 3G and Table S1. For continuous hemodynamic monitoring, PPG devices exhibit a rigid form, have limited wearability, and are susceptible to motion artifacts.^12,44^ Compared with ultrasound, the TTW sensor achieves comparable detection accuracy while offering improved portability and real-time data processing capability.^24,25^ Piezoelectric sensors, which are primarily sensitive to high-frequency signals, require calibration algorithms for hemodynamic parameter estimation,^21,31,32^ whereas the TTW sensor does not present this constraint.

### Validation of Pulse Wave Velocity Measurement for Clinical Application

PWV is an important parameter highly correlated with vascular elasticity and is used to assess specific cardiovascular risks.^12,45–47^ To translate the TTW sensor from a technical innovation into a clinically valuable tool, its performance against the Doppler ultrasound was compared. The Doppler ultrasound method determines PTT by measuring signals separately at the carotid and femoral arteries, synchronized via ECG gating. In contrast, the TTW sensor system simultaneously measures PTT between the two arterial locations in real time, enhancing efficiency and synchronization. To validate this comparison, 20 healthy volunteers aged from 22 to 60 years participated in the study (for the volunteers’ information, refer to Table S2, Supporting Information). Other clinical variables, such as the volunteers’ BMI, blood pressure, and heart rate, were also recorded. This study was approved by the HKU/HA HKW Institutional Review Board (HKU/HA HKW IRB) under reference number (UW19–490).

Blood flow Doppler ultrasound images at the carotid, brachial, and radial arteries were continuously recorded by trained physicians for 1 min each, with a sliding window size of 5 s (Figure 4A, for the details of experimental procedures, refer to Methods). The PAT was obtained by measuring the interval from the ECG R-wave to the starting point of the blood flow velocity to calculate the PTT. The distance between the two positions is divided by PTT to calculate PWV. The distance between the carotid and radial arteries was determined by summing the distance from the suprasternal notch to the carotid artery to the distance from the suprasternal notch to the radial artery. The brachial-radial artery distance was measured as the straight-line distance between the two monitoring points. Following the Doppler ultrasound test, the volunteers remained in the same position while the TTW sensors were attached to the identical arterial locations—the carotid, brachial, and radial.

**Figure 4:**
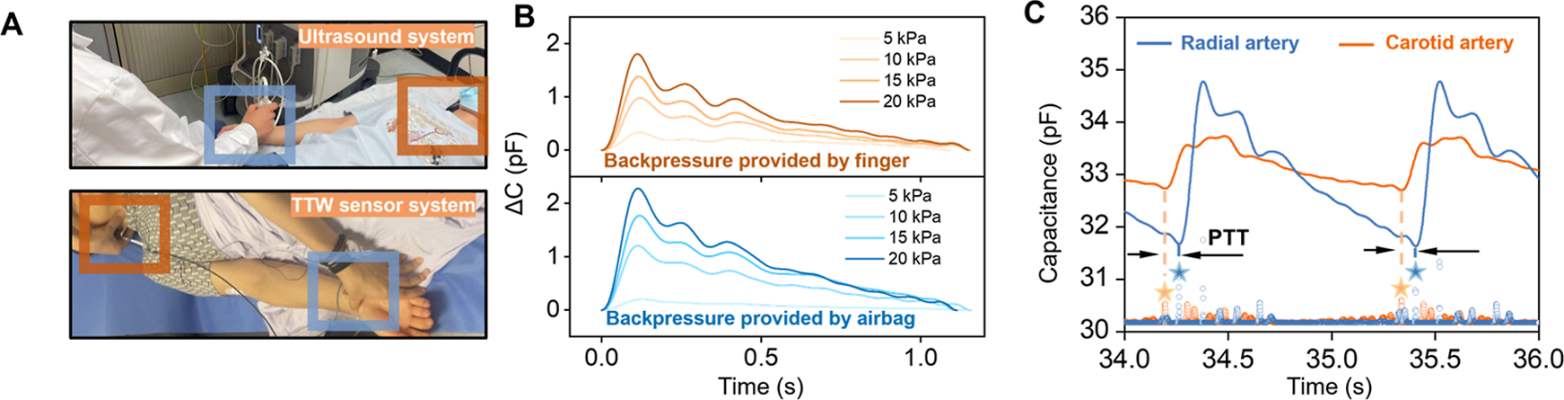
Pulse wave velocity studies on healthy individuals. (A) Demonstration of a physician’s maneuver to collect carotid-radial PWV using Doppler ultrasound and the TTW sensor system. (B) Pulse waveform collected by the TTW sensor with backpressure provided by the airbag and fingertip. (C) Illustration of calculating the pulse transit time between two arteries by the peaks of pulse curvature.

Pulse measurements are prone to motion artifacts. For example, at the carotid artery, the measurement site is in the upper neck, where the carotid artery is relatively superficial but is partially covered by the sternocleidomastoid muscle, making the signal prone to loss due to muscle contraction. During carotid pulse acquisition, subjects were instructed to rotate their heads to expose the artery and facilitate waveform detection. To ensure proper contact between the sensors and the skin, the customized airbags or fingers were applied to provide backpressure to detect the capacitive response to blood propagation (Figure 4A, for the details of using airbag system to apply pressure, refer to Figure S8 and Video S1, Supporting Information; For the details of using fingers to apply pressure, refer to Video S2, Supporting Information; For the details of sensor-skin interface stability on pulse signals with/without backpressure provided by airbag, refer to Figure S9, Supporting Information). Pulse waves acquired using fingertip localization exhibited only minimal amplitude differences compared with those obtained using the customized airbag (Figure 4B). Moreover, physician-guided fingertip positioning, combined with real-time waveform monitoring and pressure adjustment, provides greater signal stability and reduced motion-induced noise. To calculate PWV, a point-based method was used to identify the “foot” of the systole at the beginning of the pulse wave and extract the time for calculating the PTT.^48^ The curvature of the pulse wave is calculated, and the PTT is determined by identifying the local maximum curvature (For the details of the calculation process, refer to Methods, eqs 1, 2, and 3), corresponding to the foot of the wave (Figure 4C). The TTW sensor system allows the transition of vibration signals to the physician, avoiding positional shifts caused by the equipment switching, as often occurs when using a tonometer. There is a strong correlation between the carotid-radial PWV (cr-PWV) values obtained using the TTW sensor system and Doppler ultrasound (regression equation: *y =* 0.942*x* + 0.598; Pearson’s *r =* 0.88, *p* < 0.001; Figure S10A, Supporting Information). Similarly, the brachial-radial PWV (br-PWV) values showed a moderate correlation (regression equation: *y =* 0.675*x* + 4.383; Pearson’s *r* = 0.639, *p* < 0.001; Figure S10C, Supporting Information). And the mean differences in cr-PWV and br-PWV are −0.084 m/s and −1.017 m/s, respectively (Figures S10B and S10D, Supporting Information). Notably, the standard deviation of cr-PWV measurements collected from Doppler ultrasound was higher than that of the TTW sensor system (Figure S11, Supporting Information). These findings underscore its reliability in clinical settings, offering comparable accuracy with improved practicality and convenience.

Cr-PWV measured by the TTW system exhibited a significant age-dependent increase (regression equation: *y* = 0.034*x* + 7.513, *p* < 0.001; Figure 5A), with 50% of participants aged >50 years exhibiting values >10 m/s-a threshold linked to elevated cardiovascular risk. Although minor fluctuations were observed within individual groups, the overall trend demonstrates a consistent rise in cr-PWV with age for both males and females. In the age groups 20–30 and 30–50 years old, cr-PWV values remained within the healthy range, with a maximum of 9.617 m/s (mean values of subject 11). However, among volunteers over 50 years old, 4 out of 8 participants exceeded 10 m/s (3 males and 1 female), with the highest recorded value being 11.353 m/s (mean values of subject 20). Similarly, BMI correlated positively with cr-PWV (regression equation: *y* = 0.099*x* + 6.579, *p* < 0.001; Figure 5B). In contrast, br-PWV also showed a positive correlation with age (regression equation: *y* = 0.011*x* + 9.985, *p* < 0.001; Figure 5C) and BMI (regression equation: *y* = 0.019*x* + 5.967, *p* < 0.001; Figure 5D), while the trend of change was smaller than that of cr-PWV. Among the 20–30 age group, the highest br-PWV recorded was 12.382 m/s (mean value of subject 9), while the 30–50 age group reached 13.292 m/s (mean value of subject 10). In the group over 50 years old, the highest br-PWV value was 13.197 (mean value of subject 20).

**Figure 5:**
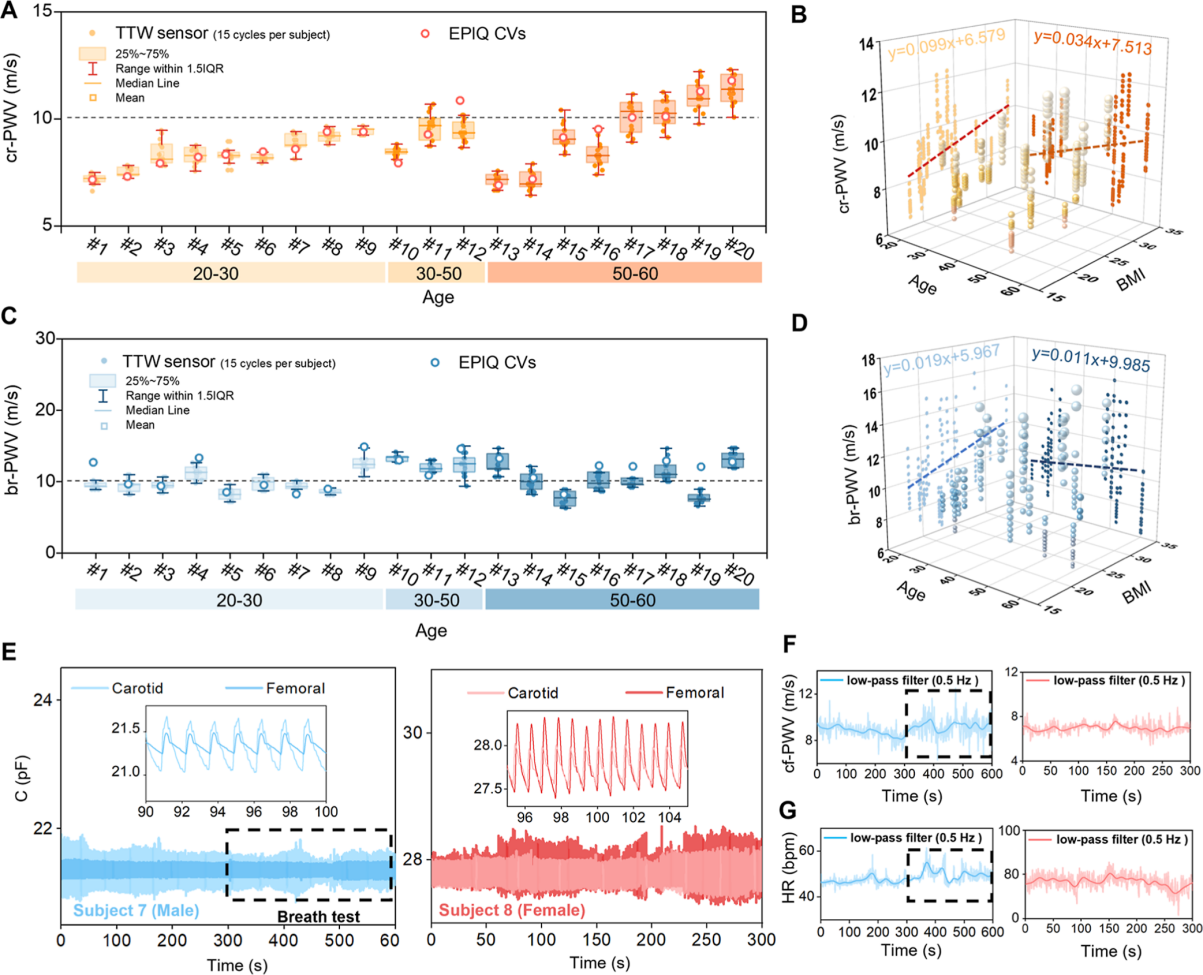
Validation of Pulse Wave Velocity Measurement. (A) Comparison of cr-PWV collected by Doppler ultrasound and TTW sensors of 20 subjects. (B) Trend in cr-PWV of healthy participants with different ages and BMIs. (C) Comparison of br-PWV collected by Doppler ultrasound and TTW sensors of 20 subjects. (D) Trend in br-PWV of healthy participants of different ages and BMIs. (E) Continuous PWV monitoring test with 10 min of continuous monitoring of carotid and femoral PWV in subject 7, including a 5 min cyclic breath-hold test; subject 8 for 5 min. (F,G) Cf-PWV and heart rate monitoring results for two subjects. *n* = 15 pulse cycles data points in the box plots of (A and C). Square, mean; center line, median; box limits, upper and lower quartiles; whiskers, 1.5 × interquartile range (IQR); points, data points, in box plots of (A and C).

The system was further tested for its ability to continuously monitor cf-PWV, which is an essential metric for assessing arterial stiffness. The study included two healthy volunteers (subject 7 and subject 8). The pulse propagation distance was calculated by adding the distance from the suprasternal notch to the carotid artery and the distance from the suprasternal notch to the femoral artery measurement point. As shown in Figure 5E, pulse signals were continuously recorded over a minimum duration of 5 min. The foot positions of the pulse waves, marking the start of the systolic upstroke, were used to calculate changes in heart rate by measuring the time intervals between two consecutive pulse cycles. The calculation of PTT followed the same methodology described in the previous section, determining the time difference between the foot positions of the carotid and femoral arterial pulse waves within the same cycle. During the 5 min prior to testing, Subject 7 exhibited a heart rate (HR) of 47 ± 1.9 bpm and a cf-PWV of 8.8 ± 0.6 m/s (measures ± standard deviations). Then, subject 7 underwent a cyclic breath-hold test, performing two voluntary breath-holds over the following 5 min. The results showed that his heart rate (49.6 ± 3.1 bpm) and cf-PWV (9.2 ± 0.9 m/s, mean ± SD) both increased as the duration of breath-holding increased, with the range of fluctuation also noticeably greater (Figure 5F,G). During the 5 min of testing, subject 8 maintained stable respiration with no detectable body movement during testing. There are no significant fluctuations in cf-PWV (7 ± 0.5 m/s, mean ± SD) or heart rate (76.7 ± 3.5 bpm). The TTW system eliminates key limitations of Doppler ultrasound by enabling simultaneous, real-time PWV acquisition at multiple arterial sites. Its tactile-transparent design allows physicians to palpate arteries during measurements, reducing positional errors and operation difficulties. The system is also capable of continuous monitoring. The test validates its utility for noninvasive, real-time assessment of cardiovascular health in clinical and everyday scenarios.

### Cardiovascular Risk Assessment Based on High-Fidelity Pulse Waveform Analysis

Notably, the system enables physicians to simultaneously extract cardiovascular risk markers from the high-fidelity waveform when monitoring PWV. The radial pulse waves of 20 subjects are shown in Figure 6A (for detail of the carotid, brachial, and radial pulse waveforms of 20 subjects participating in the PWV tests, refer to Figure S12, Supporting Information). The augmentation index (AI) as a risk marker is a widely recognized measure of the augmentation of central aortic pressure by a reflected pulse wave. It serves as an essential biomarker of arterial stiffness and an independent predictor of kidney damage.^49–51^ Reflection index (RI) and stiffness index (SI) are commonly used to assess artery stiffness, vasoactive drug effects, and endothelial function.^52,53^ Derived from radial/brachial waveform, the peripheral augmentation index (pAIx), RI, and SI can be calculated. On the radial artery, the highest values were 114% for pAIx and 80% for RI (mean value of subject 15, the oldest subject), as shown in Figures 6B and S13, Supporting Information. The pAIx, RI, and SI all showed an increasing trend with age (for the details of the calculation of cAIx, pAIx, RI, and SI, refer to Text S4, Supporting Information). For the brachial artery, the trend of risk indicators was generally consistent with that of the radial artery (Figure S14, Supporting Information). However, some subjects exhibited more dispersed results in the brachial artery, indicating that the radial artery represents a more optimal site for assessing peripheral arterial status. Central augmentation index (cAIx) in carotid arteries increased with age, with the highest value of 13.5% (mean value of subject 14, Figure S15, Supporting Information).

**Figure 6:**
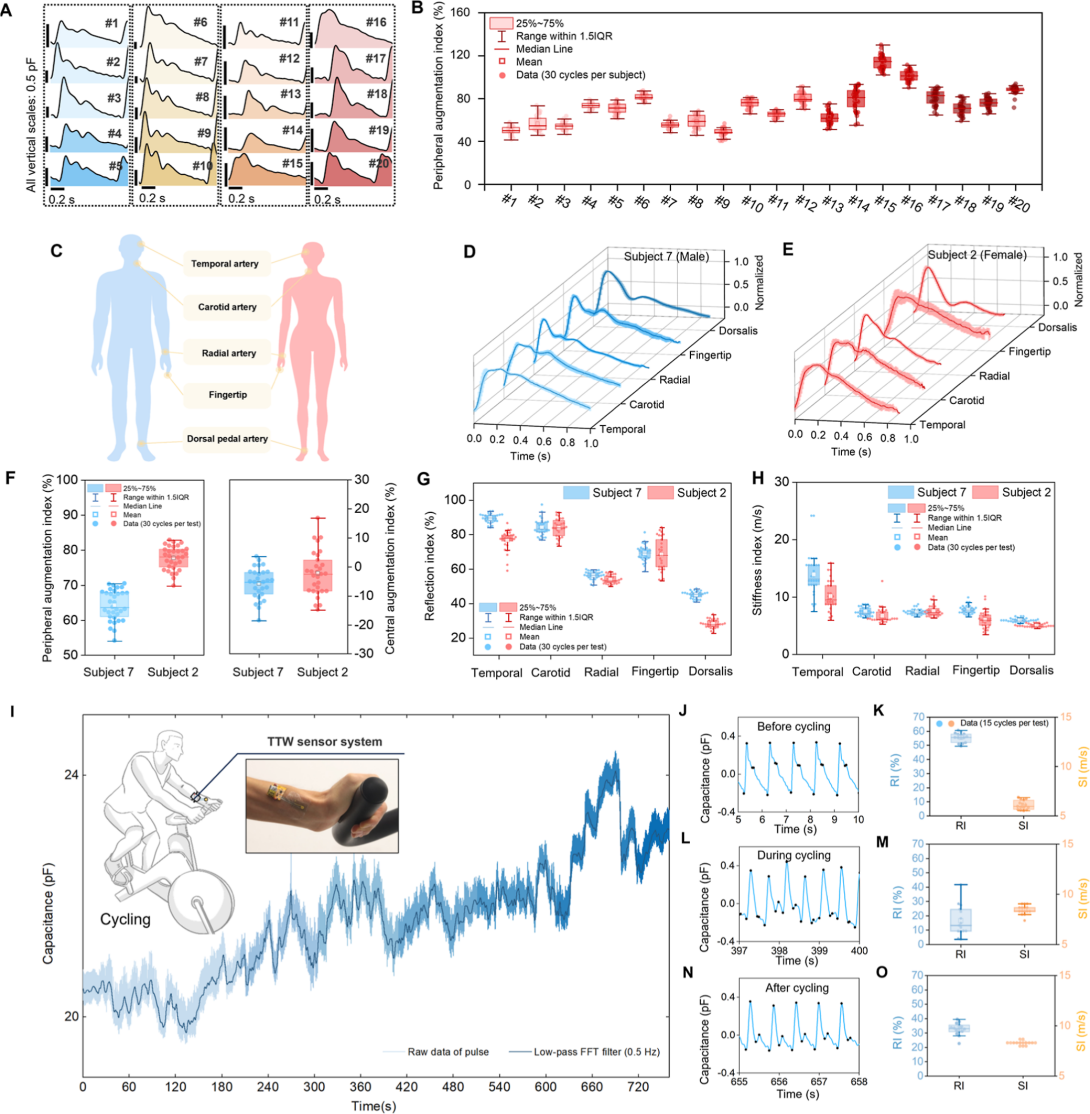
Cardiovascular assessments based on high-fidelity pulse waveform. (A) The radial pulse waves of subject 1 to subject 20. (B) Distribution of the peripheral augmentation index on radial artery of subject 1 to subject 20. (C) Measurement positions for pulse collecting of two subjects. The temporal artery, carotid artery, radial artery, fingertip, and dorsalis pedis artery. (D) Comparison of the pulse waveforms obtained from the different positions of subject 7. Shadow areas indicate the standard deviations of the results from 30 pulse wave cycles. (E) Comparison of the pulse waveforms obtained from the different positions of subject 2. (F) The central augmentation index at the carotid artery and the peripheral augmentation index at the radial artery of the two subjects. (G) Trends of reflection index at five different artery locations. (H) Trends of stiffness index at five different artery locations. (I) Continuous monitoring of radial pulse signals under different cycle states. (J–O) Changes of reflection index (RI) and stiffness index (SI) at radial artery locations during cycling. *n* = 30 pulse cycles data points in the box plots of (B, F, G, and H), *n* = 15 pulse cycles data points in the box plots of (K, M, and O). Square, mean; center line, median; box limits, upper and lower quartiles; whiskers, 1.5 × interquartile range; points, data points, in box plots of (B, F, G, H, K, M, and O).

Effective monitoring of patients with peripheral atherosclerosis requires capturing the pulse signal from multiple arterial locations to assess vascular health comprehensively. To validate the capability of the TTW sensor system to monitor a comprehensive range of peripheral arteries, the sensors were used to collect arterial waveforms from the temporal artery, carotid artery, radial artery, fingertip, and dorsalis pedis artery, as illustrated in Figure 6C. The results of pulse wave detection at five locations for two subjects (subject 7 and subject 2) are presented in Figure 6D,E. For each location, 30 consecutive pulse cycles were extracted, and the mean and standard deviation of the data were calculated. The solid lines represent the pulse curves derived from the mean of the 30 pulse cycles, while the shaded areas indicate the standard deviation. The results showed that the height of the late systolic peak for the temporal artery is higher than that of the early systolic peak. As the measurement shifts from the temporal artery to the dorsalis pedis artery, the location of the late systolic peak gradually lowers and disappears, and the location of the dicrotic notch gradually lowers and becomes more prominent. For example, the dorsalis pedis artery does not have a late systolic waveform but has an obvious diastolic peak. The pAIx values for the radial artery in two volunteers were 64.18% and 77.71%, respectively. Similarly, the cAIx values for the carotid artery were found to be −5.81% and −2.29%, respectively, all within the normal range. Young subjects generally exhibit higher arterial elasticity and compliance, resulting in slower pulse wave propagation. Consequently, the reflected wave returns to the aorta during the late systole, leading to a high late systolic peak, often producing a negative cAIx (Figure 6F).^54,55^ In this study, RI and SI values were calculated from 30 consecutive pulse cycles at 5 different measurement locations and plotted on scatter plots to visualize waveform variations across sites (Figure 6G,H). For subject 7, RI decreased from 89.42% (temporal artery) to 44.88% (dorsalis pedis), while SI declined from 13.88 m/s (temporal artery) to 5.91 m/s (dorsalis pedis). Similarly, for subject 2, the RI at the same locations decreased from 76.66% (temporal artery) to 27.99% (dorsalis pedis), with corresponding SI declining from 10.11 m/s (temporal artery) to 4.99 m/s (dorsalis pedis). The results showed that both RI and SI values decreased consistently as the measurement location shifted from the temporal artery to the dorsalis pedis artery, corresponding to the increasing distance from the aortic root. The arteries located farther from the aortic root accumulated more reflected pressure waves at the peaks, resulting in higher systolic pressure, and distal arteries (with stronger muscles) have lower compliance and less elastic tissue, leading to decreased RI. Additionally, the farther down the arterial tree, the longer it takes for the reflected waves caused by the closure of the aortic valve to reach the distal arteries, causing decreased SI, which is consistent with the physiological results. The results of pulse wave acquisition at different locations validate the TTW sensor system’s capability to realize cardiovascular risk profiling monitoring.

### Dynamic Cardiovascular Monitoring Under Exercise-Induced Stress

The cardiovascular response to exercise is also noteworthy, but this is rarely monitored continuously. The TTW sensor has shown its accuracy in measuring PWV and cardiovascular risk markers across different arteries. Based on those findings, the system’s ability to detect cardiovascular risk was further evaluated during exercise. To enable continuous monitoring under dynamic conditions, a lightweight signal acquisition module was developed (only 0.315 g without battery, refer to Methods; Figures S16 and S17, Supporting Information). The pulse data are transmitted via WiFi/Bluetooth, enabling real-time display during physical activity. In the cycling test, the volunteer (subject 7) first rested for 3 min. The exercise phase involved cycling on a spinning bicycle at a resistance level of 5 and a speed of 20 km/h for 10 min. The TTW sensor was over the radial artery with 3 M tape, and the fPCB circuit was attached to the arm for continuous radial pulse wave monitoring (Figure 6I). At rest, the heart rate was 63 bpm, the wrist pulse showed an average 10 s RI value of 55%, and the average 10 s SI of 6.93 m/s, with a high-positioned diastolic peak showing subdued morphology (Figure 6J,K). During cycling, significant changes were observed. The heart rate increased to 136.2 bpm, the RI decreased to 18% (67% decrease), and the SI increased to 8.26 m/s (19% increase). Additionally, the systolic peak amplitude increased (+0.12 au) and the diastolic peak descended with enhanced prominence (7.1% of primary peak height), while the late systolic peak disappeared (Figure 6L,M). One minute after cycling, the heart rate decreased to 105 bpm, the RI increased to 33%, and the SI slightly decreased to 8.23 m/s. The diastolic peak location was higher than during exercise but lower than the pre-exercise resting state, with a height of 11.3% of the primary peak (Figure 6N,O). These changes indicate greater vascular expansion and high PWV caused by higher cardiac output (higher pulse rate) during cycling. Vasodilation reduces reflected wave amplitude, while the combination of high PWV and shorter heart resting time contributes to a lower SI. In addition, the frequency spectrum of the continuously collected pulse wave provides real-time tracking of heart rate and detailed monitoring for exercise-induced changes during cycling (Figure S18). These capabilities are crucial for understanding the dynamic cardiovascular adaptation that occurs during physical activity, providing quantitative insights into how the cardiac and vascular systems respond to increased physiological demands.

The pre- and postexercise trends in blood pressure and br-PWV were preliminarily tested. A healthy volunteer (subject 9) performed a 5 min squatting exercise. The physician used the TTW sensor system to record br-PWV and the cuff blood pressure monitor (OMRON T31) to measure the blood pressure during the rest. The data showed a significant decrease in both blood pressure and br-PWV as the rest period increased, the blood pressure decreased from hypertensive levels (Systolic Blood Pressure: 143 mmHg, Diastolic Blood Pressure: 85 mmHg) to normal levels (Systolic Blood Pressure: 117 mmHg, Diastolic Blood Pressure: 73 mmHg) after 12 min (Figure S19, Supporting Information). Results indicate a strong correlation between these two indicators. Overall, the results demonstrated that exercise induced significant cardiovascular adaptations, which were effectively recorded by the TTW system in real time. This highlights the versatility and effectiveness of the TTW system as a reliable tool for noninvasive, continuous cardiovascular assessment and for tracking cardiovascular risk profiling during exercise.

## Conclusion

This study demonstrates a TTW sensor system capable of continuous, clinician-integrated cardiovascular monitoring, addressing critical limitations in current PWV technologies. Unlike bulky one-step PWV devices, the TTW sensor system achieves high-fidelity hemodynamic profiling (central/peripheral PWV, cAIx, pAIx, heart rate, etc.) through a compact, wearable form factor.

Tactile transparency bridging palpation and precision: The TTW sensor’s breakthrough lies in its ultrathin (<150 μm) tactile-transparent design, which uniquely preserves physicians’ ability that allows the physician to easily sense and measure the pulse wave at a specific location, enabling efficient assessment of arterial health while capturing high-fidelity pulse waveforms, providing a practical platform for resource-limited settings. This dual capability addresses a fundamental limitation in wearable sensing: prior rigid sensors (e.g., tonometry) sacrificed tactile feedback for quantitative accuracy. Our characterization tests validate the system’s excellent linearity (*R* ^2^ > 0.975), sensitivity (0.06 kPa^–1^), and durability (>10^4^ cycles) metrics critical for long-term monitoring, while its small size and convenience support suitability for wearable applications.

Synchronized PWV accuracy and clinical insights: In 20 healthy volunteers, the TTW sensor system exhibited Doppler-level accuracy for carotid-radial PWV (Pearson’s *r* = 0.88, *p* < 0.001), reducing variability caused by interdevice asynchronization. The strong correlations between cr-PWV and age/BMI (*y* = 0.034*x* + 7.513, *p* < 0.001; *y* = 0.099*x* + 6.579, *p* < 0.001) reaffirm its validity as an arterial stiffness marker. In contrast, the trends in br-PWV with age and BMI were not as pronounced, suggesting that br-PWV may not be a valid measure of arterial stiffness associated with these variables. These findings emphasize the importance of selecting appropriate PWV metrics based on specific clinical or research applications.

Functional Versatility for Cardiovascular: The TTW system also demonstrated its capability in monitoring peripheral atherosclerosis by successfully detecting superficial arterial waveforms from multiple sites (e.g., carotid, radial, etc.). High-fidelity pulse waveforms collected from these sites enabled the calculation of key indices such as the peripheral augmentation index, central augmentation index, reflection index, and stiffness index. These capabilities position the TTW system as a valuable tool for monitoring both central and peripheral cardiovascular health and detecting early signs of atherosclerosis. Moreover, the TTW system successfully captured real-time cardiovascular changes across pre-exercise, exercise, and postexercise recovery periods, demonstrating feasibility for dynamic and continuous monitoring in applications such as fitness tracking, rehabilitation, and clinical exercise testing.

Limitations and Future Directions: Despite these promising results, there are areas for improvement in the current work. For high-fidelity arterial waveform measurements, motion artifacts remain a major challenge in wearable applications, particularly at the carotid artery. This is due to its deeper anatomical location, reduced signal amplitude from the high elastic tissue content of the aortic wall, and signal loss from variations in sensor-skin contact pressure and positional shifts caused by sternocleidomastoid muscle activity. In clinical PWV measurements with physician involvement, neck motion is minimal, and manual localization with fingertip pressure allows for stable signal acquisition. For wearable use during physical activity, reliable signal acquisition would require both hardware improvements (e.g., incorporating a micropump-based module to apply controlled pressure to the carotid site) and software algorithms capable of selectively identifying pulse waveforms during rest intervals within movement sequences for reliable cardiovascular parameter estimation. Regarding the peripheral augmentation index, values in this study were derived from high-fidelity waveforms acquired by the TTW sensors and compared only across participants due to equipment constraints. The absence of a direct comparison with PWV measurements obtained from tonometry is another limitation. Future studies involving validation against invasive carotid catheterization and tonometry will be essential to confirm the system’s accuracy and emphasize its unique advantages in continuous and portable monitoring.

Additionally, validating the TTW system across diverse populations and exploring its potential for long-term cardiovascular health monitoring will further strengthen its clinical utility. Advancements in data processing, possibly incorporating artificial intelligence, can enhance the interpretation of the collected data, providing clinicians with actionable insights. Overall, the TTW system, with its ease of use, continuous monitoring capabilities, and portability, represents a significant leap forward in advancing cardiovascular diagnostics and personalized healthcare.

## Methods

### Fabrication of the TTW Sensor

First, a layer of polydimethylsiloxane (PDMS, Sylgard 184 silicone elastomer, cross-linking ratio of 10:1) with a thickness of 70 μm was spin-coated (1500 rpm, 30 s) on a glass substrate (70 × 70 mm) and cured at 70 °C for 1 h (Figures S1A,B, Supporting Information). The promotion solution was prepared (isopropanol (IPA), deionized water (DI), and A-174 solution (Aladdin), proportion by volume ratios of IPA/DI/A-174 = 50:50:1), and the solution was stirred for 30 s and stood for at least 2 h. The glass sheet with the PDMS film was submerged in the prepared promotion solution for 20 min, taken out, and dried for 20 min, finally dried after rinsing with IPA (Figure S1C, Supporting Information). Two μm Parylene C (pressure: 15 mTorr, furnace set point: 690 °C) was deposited on the PDMS thin film as an adhesive interlayer (Figure S1D, Supporting Information). Then 100 nm of Au was sputtered (Quorum Q150T ES Plus Sputter) onto the processed PDMS film (Figure S1E, Supporting Information). To form patterns by photolithography, a photoresist layer (photoresist layer: AZ 4620, AZ Electronic Materials) was spin-coated (3000 r.p.m., 30 s) on the Au layer, followed by prebaking at 110 °C for 5 min. After alignment (URE-2000/35AL Deep UV, IOE, CAS), it was exposed to UV light for 45 s and developed in AZ 400k solution for 90 s to form the design pattern. Then, the Au layer was wet etched by the Au etchant (I_2_/KI solution, I_2_/KI: water = 1:4:40). Rinsing in acetone removed the remaining photoresist. After completing the above steps, the gold remained firmly adhered on the PDMS surface (Figure S1F, Supporting Information). Then the aqueous dispersion of single-layer graphene oxide (concentration of 2 mg/mL, Graphene China, average lateral particle size was ≈0.5–5.0 μm, and the thickness was ≈0.335–1.000 nm) was drop-coated onto the electrode surface and dried at 50 °C for 6 h to form a 3 μm graphene oxide paper-like thin film, and then drop-coated again after drying to finally form a 5 μm graphene oxide paper film with uniform thickness (Figure S1G). Finally, a 5 μm thick optical acrylic film (acrylate adhesive, parylene ensured the adhesive interface) was used to bond the two electrodes with graphene oxide dielectric films together to complete the fabrication of the TTW sensor, with a total thickness of about 150 μm (Figures S1H and S1I, Supporting Information), for detail of thickness, surface flatness of different GO films generated by different GO concentration aqueous, refer to Figure S3, Supporting Information.

### Fabrication Process of the Airbag System

The process of producing the airbag system began by using silicone glue to secure a small balloon (GINCHO, Japan) inside a 3D-printed housing (ANYCUBIC PHOTON D2, Shenzhen Anycubic Technology Co., Ltd.). Then, the balloon was connected to an airbag pressure gauge via a silicone tube (outer diameter 8 mm, inner diameter 5 mm) and a three-way valve, and sealed with silicone glue; The optical image of the customized airbag system is shown in Figure S8B, Supporting Information

### Lightweight Signal Acquisition Module

To enhance the wearability and usability of the sensor system, a small and lightweight flexible printed circuit board (fPCB) was designed for dynamic monitoring during cycling. The fPCB dimensions were 20 mm × 20 mm and weighed only 0.315 g (battery weight: 1.453 g). An MCU (ESP32-C6, Espressif Systems, China) was used for wireless communication, function control, signal reading, and transmission. The capacitive signals were acquired by a capacitance-to-digital converter (FDC1004, TEXAS INSTRUMENTS, USA). The MCU accessed the FDC1004 data via the I2C protocol and sent it to a mobile device via WiFi/Bluetooth to realize real-time display of the pulse waveforms. The microcontroller used Arduino programming. For details of the schematic design of the signal sampling module, refer to Figure S17, Supporting Information.

### Experiments with Human Participants

Blood flow velocity and pulse wave collection were performed with the full informed consent of the volunteers. All human experiments were conducted in accordance with protocols approved by the Institutional Review Board of The University of Hong Kong/Hospital Authority Hong Kong West Cluster (HKU/HA HKW IRB), China, and guidelines were followed.

### PWV Monitoring Based on Doppler Ultrasound

No significant differences were observed between PWVs measured simultaneously with two ultrasound devices and those obtained sequentially with a single ultrasound device (Figure S11 and Text S5, Supporting Information). Twenty volunteers of different genders and ages (22–60) were measured by a single ultrasound device method (the details of the subjects see Table S2, Supporting Information). The specific ultrasound data acquisition process is as follows. The subject was optimally positioned in a supine orientation with a slight elevation of the head, facilitating accessibility to the carotid and femoral arteries while minimizing muscular interference. Subjects were connected to the ultrasound system (Philips EPIQ CVs)’s integrated ECG leads to detect cardiac electrical activity, facilitating ECG-gated measurements that synchronize PWV assessments with the cardiac cycle. This ensured that the measurements were taken at the same phase of the cardiac cycle, thereby enhancing the accuracy and consistency of the results. The vascular examination preset on the Philips EPIQ was selected to fine-tune the imaging parameters for vascular assessments. Depth, gain, and frequency settings were meticulously adjusted to ensure high-resolution imaging of the arterial structures. A high-frequency L12–3 linear array transducer was employed to provide the requisite resolution for visualizing arterial walls and lumen. Ultrasound gel was applied to the transducer, which was then positioned over the artery to obtain a clear longitudinal view. Blood flow images were continuously recorded at the carotid artery for 1 min. The location of the carotid artery was marked, and the transducer was subsequently repositioned over the radial artery or brachial artery, with a similar longitudinal view obtained and marked. The straight-line distances between the marked carotid, brachial, and radial artery locations were measured. The carotid-to-radial distance was calculated by combining the measurements from the suprasternal notch to the carotid artery and from the notch to the radial artery, while the brachial-to-radial distance was measured as the straight-line distance between the two monitoring points. Concurrently, the time elapsed for the pulse wave to propagate from the carotid to the radial artery was recorded. PWV was determined using the formula: PWV = Distance/Time delay. The time delay was calculated by measuring the time from the ECG R-wave to the starting point of the blood flow waveform. After averaging the measurement results, PTT was calculated by difference, and cr-PWV or br-PWV were further calculated. Postexamination, the transducer was cleaned with a disinfectant wipe and stored in accordance with recommended protocols.

### PWV Monitoring Based on the TTW Sensor System

For the cr-PWV and br-PWV monitoring, the physician used the TTW sensor system for simultaneous multichannel pulse wave collection at the same arterial location, using finger pressure to ensure stable contact between the sensor and the skin to capture high-fidelity pulse waveforms (for detailed experiment process see Video S2, Supporting Information). For the continuous monitoring of cf-PWV, the physician used the TTW sensor to locate the proper pulse acquisition sites on the right carotid artery and right femoral artery of the subject. The sensors were then fixed by using 3 M Tegaderm film tape, and an airbag system was applied to ensure stable signal acquisitions by providing consistent pressure at measurement sites. The acquisition circuit (PCAP04-EVA-KIT, ScioSense) for PWV monitoring was configured to capture the capacitance response at a rate of 1000 points per second to ensure accurate measurement of the pulse transit time. Moreover, the TTW system demonstrated superior precision in simultaneous measurements, achieving a lower standard deviation (SD: 0.00393) compared to the two ultrasound devices (SD: 0.00674, Figure S11C, Supporting Information).

### Peripheral Artery Pulse Monitoring Based on the TTW Sensor System

For pulse signal detection at the fingertips and dorsalis pedis artery, the sensors were secured using 3 M Tedagerm film due to the superficial nature of the arteries in these regions. This setup enabled optimal signal acquisition. In contrast, the temporal and radial arteries have a concave skin surface, and the carotid artery is located deeper beneath the skin. To ensure stable attachment and additional pressure, a silicon pad was added between the 3 M Tedagerm film tape and the TTW sensor. The pressure exerted on the skin surface by the two sensor fixation methods was measured using a MARK-10 force gauge. The pressure was approximately 0.55 kPa when the sensor was fixed with only the 3 M Tedagerm film tape, and around 5 kPa when the sensor was fixed with the added silicon pad. The pulse wave signal is captured by the acquisition circuitry (PCAP04-EVA-KIT, ScioSense) at a frequency of 350 Hz.

### Signal Processing of Pulse Wave Data for PWV Monitoring

Noise and irrelevant features are removed from the raw data to enhance the accuracy and efficiency in feature extraction. Initially, the raw data are processed using a band-pass filter with cutoff frequencies set at 0.5 and 10 Hz. This range is selected to eliminate low-frequency fluctuations and high-frequency noise, considering the typical human pulse frequency of 40 to 120 beats per minute (approximately 0.6 to 2 Hz). For pulse wave signals *P*(*t*), the curvature κ at each point is calculated using the discrete approximation

1 $$\kappa =\frac{\left|P^{\prime \prime }\left(i\right)\right|}{{\left[1+{\left(P^{\prime }\left(i\right)\right)}^{2}\right]}^{3/2}}$$

where *P′*(*i*) and *P*″(*i*) represent the first and second derivatives approximated by finite differences

2 $$P^{\prime }\left(i\right)=\frac{P\left(i+1\right)-P\left(i-1\right)}{2\Delta t}$$

3 $$P^{\prime \prime }\left(i\right)=\frac{P\left(i+1\right)-2P\left(i\right)+P\left(i-1\right)}{{\Delta t}^{2}}$$

The onset of each pulse corresponds to the local minimum of the curvature contour before the peak of systole, while the peak shows the maximum curvature value. By recognizing the inherent geometric features in the pressure waveform based on the curvature method, the reliable identification of key pulse wave features and obtain accurate pulse wave onset points are achieved.

### Characterization and Measurements

The mechanical pressure was provided by a tensile tester (ESM 303), and the pressure data were collected using Mark-10. The sensitivity and step response of the sensor were measured using an impedance analyzer (HIOKI IM3570, Japan, 50 kHz test frequency and 1 V capacitive mode). Repeatability and petal detection tests of the sensor were performed by a capacitance acquisition board (PCAP04-EVA-KIT, ScioSense). The SEM images and EDX spectroscopies were collected using QUANTA FEG 450. Dielectric spectroscopy tests of graphene oxide thin film samples were performed at room temperature on a dielectric impedance temperature spectrum Instrument (DMS 1000, Bailibo Technology Co., Ltd., China). Ultrasound image data were processed by RadiAnt DICOM Viewer. Data analysis and visualization were conducted using OriginPro 2021 and Python 3.12.

## Supplementary Material











## Data Availability

The data that support the findings of this study are available from the corresponding author upon reasonable request.

## Notes

The text part includes dielectric characterization of GO films (Text S1), principles of the TTW sensor detection (Text S2), the experimental setup for fingertip pressure test for physicians (Text S3), cardiovascular indicators calculation and comparison (Text S4 and S5). Other features and results are shown in Figures S1–S19. Supplementary tables include a technical comparison of continuous hemodynamic monitoring (Table S1) and volunteer information (Table S2) (PDF)
Video S1. Simultaneous acquisition of radial, brachial, and carotid pulses using a balloon airbag for PWV measurement (MP4)
Video S2. Simultaneous acquisition of radial and carotid pulses using finger pressure for PWV measurement (MP4)
Hospital Authority Hong Kong West Cluster (PDF)
Informed Consent Form (20 subjects, Chinese and English versions) (PDF)
