# Supplementary material for: Tactile-Transparent Wearable Sensor for Clinician-Friendly Pulse Wave Velocity Monitoring and Cardiovascular Risk Profiling
Source: ACS Nano. 2025 Sep 5;19(36):32822–35. doi: 10.1021/acsnano.5c11375 (PMC12444991; doi:10.1021/acsnano.5c11375)
Supplement: Supplementary file 1 [file nn5c11375_si_001.pdf]

Supporting Information

Tactile-Transparent Wearable Sensor for Clinician-Friendly Pulse Wave Velocity Monitoring and Cardiovascular Risk Profiling

Senlin Hou[1,#], Xiaotong Chen[2,#], Dani S. Assi[3,#], Yu Feng[1], Chun-Ka Wong[4], Jingting Tian[2], Jian Li[5], Xiaodong Yu[1], Binghe Guan[6], Xiaohu Zhu[7], Xinge Yu[5], Xinyue Li[8], Vellaisamy A. L. Roy[3], Jiangang Shen[2,*]and Wen Jung Li[1,*]

1 Department of Mechanical Engineering, City University of Hong Kong, Kowloon, Hong Kong.

2 School of Chinese Medicine, The University of Hong Kong, Hong Kong.

3 School of Science and Technology, Hong Kong Metropolitan University, Ho Man Tin, Hong Kong.

4 Department of Medicine, School of Clinical Medicine, Li Ka Shing Faculty of Medicine, The University of Hong Kong, Hong Kong.

5 Department of Biomedical Engineering, City University of Hong Kong, Kowloon, Hong Kong.

6 Preventive Treatment Center, Shenzhen Bao'an Authentic TCM Therapy Hospital, Guangdong Province, China

7 Ultrasound Medicine Department, Shenzhen Bao'an Authentic TCM Therapy Hospital, Guangdong Province, China.

8 Department of Data Science, City University of Hong Kong, Kowloon, Hong Kong.

#: Equal contribution authors

*: Corresponding Email: shenjg@hku.hk; wenjli@cityu.edu.hk

Supplementary Notes

Text S1: Dielectric characterization of paper-like graphene oxide (GO) films for the TTW sensors.

Text S2: The TTW sensor detection principles and theoretical explanation.

Text S3: The experimental setup for fingertip pressure test for physicians obtaining pulse waves.

Text S4: Cardiovascular indicators calculation: augmentation index, reflection index, and stiffness index.

Text S5: Comparison of Doppler ultrasound methods for measuring carotid-radial PWV.

Supplementary Figures

Figure S1. Fabrication process of the tactile-transparent wearable sensor.

Figure S2. Dielectric spectroscopy of paper-like graphene oxide film.

Figure S3. The influence of GO aqueous dispersions concentration on the thickness and surface flatness properties of the film, formed at a temperature of 50℃.

Figure S4. Energy-dispersive X-ray (EDX) spectroscopies of paper-like GO film at different temperatures.

Figure S5. Tensile test results of thin paper-like graphene oxide films at different annealing temperatures.

Figure S6. Fingertip pressure test for physicians obtaining pulse waves.

Figure S7. Correlation coefficient between pressure and capacitance variation.

Figure S8. Airbag system for applying pressure at the PWV test sites.

Figure S9. Validation of the stability of a customized airbag-enhanced sensor-skin interface.

Figure S10. Pearson's correlation plots and Bland-Altman plots.

Figure S11. Measurement of PWV based on Doppler ultrasound.

Figure S12. From left to right are the carotid, brachial, and radial pulse waveforms of 20 subjects participating in the PWV tests.

Figure S13. Distribution of cardiovascular risk assessment indicators on the radial artery in 20 subjects.

Figure S14. Distribution of cardiovascular risk assessment indicators on the brachial artery in 20 subjects.

Figure S15. Distribution of cardiovascular risk assessment indicators on the carotid artery in 20 subjects.

Figure S16. Optical images of the TTW sensor system.

Figure S17. Schematic design of the signal sampling module.

Figure S18. Continuous wavelets transform radial pulse waves during the cycling test.

Figure S19. Relationship between blood pressure and brachial-radial pulse wave velocity after high-intensity anaerobic exercise.

Supplementary Tables

Table S1. Technical comparison of continuous cardiovascular monitoring.

Table S2. Volunteer information.

Supplementary Videos

Video S1. Simultaneous acquisition of radial, brachial, and carotid pulses using a balloon airbag for PWV measurement (.MP4).

Video S2. Simultaneous acquisition of radial and carotid pulses using finger pressure for PWV measurement (.MP4).

Text S1: Dielectric characterization of paper-like graphene oxide (GO) films for the TTW sensors**.**

GO films for the dielectric spectroscopy test were prepared by drying aqueous dispersions of graphene oxide at 50 °C for 6 hours, yielding paper-like films with a thickness of ~15 μm. To investigate temperature effects on the dielectric properties of GO films, these films were further annealed at 100 °C and 150 °C for 6 hours.

Dielectric spectroscopy was performed under two pressure conditions (0 kPa and 100 kPa) across a frequency range of 10 Hz–10 MHz. At 0 kPa (simulating the low-pressure phase in TTW sensors), the dielectric constant (ε_r) increased with drying temperature. At 50 kHz, ε_r rose from 1.32 (50 °C-dried) to 8.53 (150 °C-dried), accompanied by a marked increase in dissipation factor (D) from 0.21 to 402.18 (**Figures S2A and S2C**). This behavior reflects the dominance of air ($\varepsilon_{air}\approx1$) in the low-pressure state, as interfacial voids.

Under 100 kPa (simulating the high-pressure phase), ε_r exhibited significant enhancement at the same temperature. At 50 kHz, ε_r reached 2.21 for 50 °C-dried films and 47.7 for 150 °C-dried films, with dissipation factors of 0.39 and 80.12, respectively (**Figures S2D and S2F**). The elevated ε_r at higher temperatures correlates with reduced oxygen-containing functional groups (*e.g.*, hydroxyl, epoxy) upon thermal annealing, which enhances graphitic domain alignment and polarization[1]. However, excessive drying temperatures (*e.g.*, 150 °C) drastically increased the Young's modulus due to hydrophilicity loss, rendering the films brittle and unsuitable for flexible sensor applications (**Figure S4**). Tensile testing confirmed that 50 °C-dried GO films retained sufficient mechanical compliance while maintaining moderate dielectric performance and were selected as the dielectric layer for TTW sensors, balancing flexibility and capacitive sensitivity (**Figure S5**).

Text S2. The TTW sensor detection principles and theoretical explanation.

The TTW sensor is constructed using a layer of GO film covering upper/lower electrodes, and due to the rough surface of the GO film and the fact that it does not fully fit the electrodes, the following features affect the capacitance accordingly: Interfacial air layer, d_{air} (determined by surface roughness), effective contact area, A_{eff} ($A_{eff,0} < A_0$, A_0 is electrode geometric area), and initial air volume fraction of GO layer defects, f_{defect} ($f_{defect,0} \approx 50\%$).

Pressure-Dependent Capacitance Response

Low-Pressure phase (Pressure (P) < 10 kPa, sensitivity 0.06 kPa^{-1}, **Figure 3B**):

Compression of the interfacial air layer (d_{air}) and increase in effective contact area (A_{eff}) dominate.

$$d_{air} = d_{air,0} - k_d P \quad (1)$$

k_d is the air layer compression factor.

$$A_{eff} = A_{eff} + k_A P \quad (2)$$

k_A is the contact coefficient.

In the low-pressure phase, the direction of the electric field is perpendicular to the interface between the air and GO layers, so the effective dielectric constant is calculated using the series mixing rule[2]. Initial air volume fraction $f_{air,0} \approx 50\%$, dielectric constant of GO and air are ε_{GO} = 3[3], ε_{air} = 1, respectively.

$$\frac{1}{\varepsilon_{eff}} = \frac{f_{air,0}}{\varepsilon_{air}} + \frac{1-f_{air,0}}{\varepsilon_{GO}} \quad (3)$$

The calculated results are ε_{eff} = 1.5, close to the experimental values (1.32, **Figure S2A**), and the model is reasonable. The void within the GO layer is not significantly compressed, and the overall dielectric constant enhancement is limited.

Capacitance can be expressed as:

$$C = \frac{\varepsilon_0 \varepsilon_{eff} A_{eff}}{d_{air}} \propto \frac{A_{eff}}{d_{air}} \propto \frac{k_A P}{k_d P} \propto \alpha P \quad (4)$$

The actual contact area increases at a slower rate ($A_{eff} \propto \sqrt{P}$), so the capacitance grows linearly with pressure.

High-Pressure phase (10 kPa < P < 250 kPa, sensitivity: 0.03 kPa^{-1}, **Figure 3B**):

The effective dielectric constant increased (ε_{eff}) by compressing intra-GO defects. In the high-pressure phase, the intermediate air layer is fully compressed, and the contact area converges with the electrode geometry. The residual air gap in the GO film is randomly distributed and further compressed, the air volume fraction decreases, and the dielectric constant increases. The effective dielectric constant is calculated using the parallel mixing rule.

$$\varepsilon_{eff} = \varepsilon_{GO}\left(1 - f_{defect}\right) + \varepsilon_{air} f_{defect} \quad (5)$$

$$f_{defect} = f_{defect,0} - k_f P \quad (6)$$

When the defects in the GO film are compressed (100 kPa), assume the air volume is reduced to 20%, that of GO is up to 80%, the dielectric constant is calculated as ε_{eff} = 2.6, close to the experimental values (2.2, **Figure S2D**). The void within the GO layer is not significantly compressed, and the overall dielectric constant enhancement is limited.

Capacitance can be expressed as:

$$C = \frac{\varepsilon_0 \varepsilon_{eff} A_{GO}}{d_{GO}} \propto \varepsilon_{eff} \propto k_f P \quad (7)$$

The volume of defects within GO film linearly reduced and the capacitive response increased.

The linear sensitivity of the TTW sensor results from a staged pressure response mechanism: 1. Low-pressure phase: air layer compression synergizes with contact area expansion to achieve high sensitivity. 2. High-pressure phase: compression of defects within the GO layer leads to a linear increase in dielectric constant and maintains full-domain linearity. Sensitivity and linear range can be further optimized by structural design (surface roughness, initial air layer) and material tuning (defect density).

Text S3. The experimental setup for fingertip pressure test for physicians obtaining pulse waves.

To determine the pressure that physicians were feeling when they felt the pulse, the physicians were blindfolded and asked to measure the pulse wave at the carotid and radial artery. When the pulse wave was felt, the appropriate pressure was applied to the force gauge (Mark-10 Corp, Copiague, NY), and this process was repeated three times to ensure the reliability of the results (**Figure S6**).

Text S4. Cardiovascular indicators calculation: augmentation index, reflection index, and stiffness index.

The augmentation index (AI) is a widely recognized measure of the augmentation of central aortic pressure by a reflected pulse wave.

The central augmentation index (cAIx) is defined as augmentation pressure (AP), the difference between the systolic pressure (P1) and late systolic pressure (P2) peaks of the central arterial waveform, to a percentage of the height of the systolic pressure[4].

$$cAIx = \frac{AP}{P1} = \frac{P2 - P1}{P1} \times 100$$

In contrast, the peripheral augmentation index (pAIx) is defined as the ratio of the late systolic pressure (P2) to systolic pressure (P1) for peripheral arterial waveforms[5].

$$pAIx = \frac{P2}{P1} \times 100$$

The reflection index (RI) quantifies the relative height of the diastolic peak as a percentage of the total amplitude of the digital volume pulse (DVP) waveform[6].

$$RI = \frac{P3}{P1} \times 100$$

The stiffness index (SI) is calculated by dividing the subject's height by the time between the systolic and diastolic peaks[7,8].

$$SI = \frac{Subject\ height}{\Delta T_{DVP}}$$

Text S5. Comparison of Doppler ultrasound methods for measuring carotid-radial PWV.

Although the ultrasound device supports multiple probes, it can only use one linear probe at a time to detect arterial blood flow velocity. First, Doppler blood flow velocities were simultaneously obtained at two locations using two ultrasound devices (Philips EPIQ CVs and GE Vivid, **Figure S11A**), with the electrocardiogram (ECG) used as a reference for alignment. This process was complex and needed two trained physicians to operate at the same time. To simplify the process, a single device (Philips EPIQ CVs) was used to measure the pulse arrival time (PAT) at each location sequentially, and the time difference was calculated to determine PWV. A comparison of the two methods showed no significant difference in the PWV measurements (**Figure S11B**).

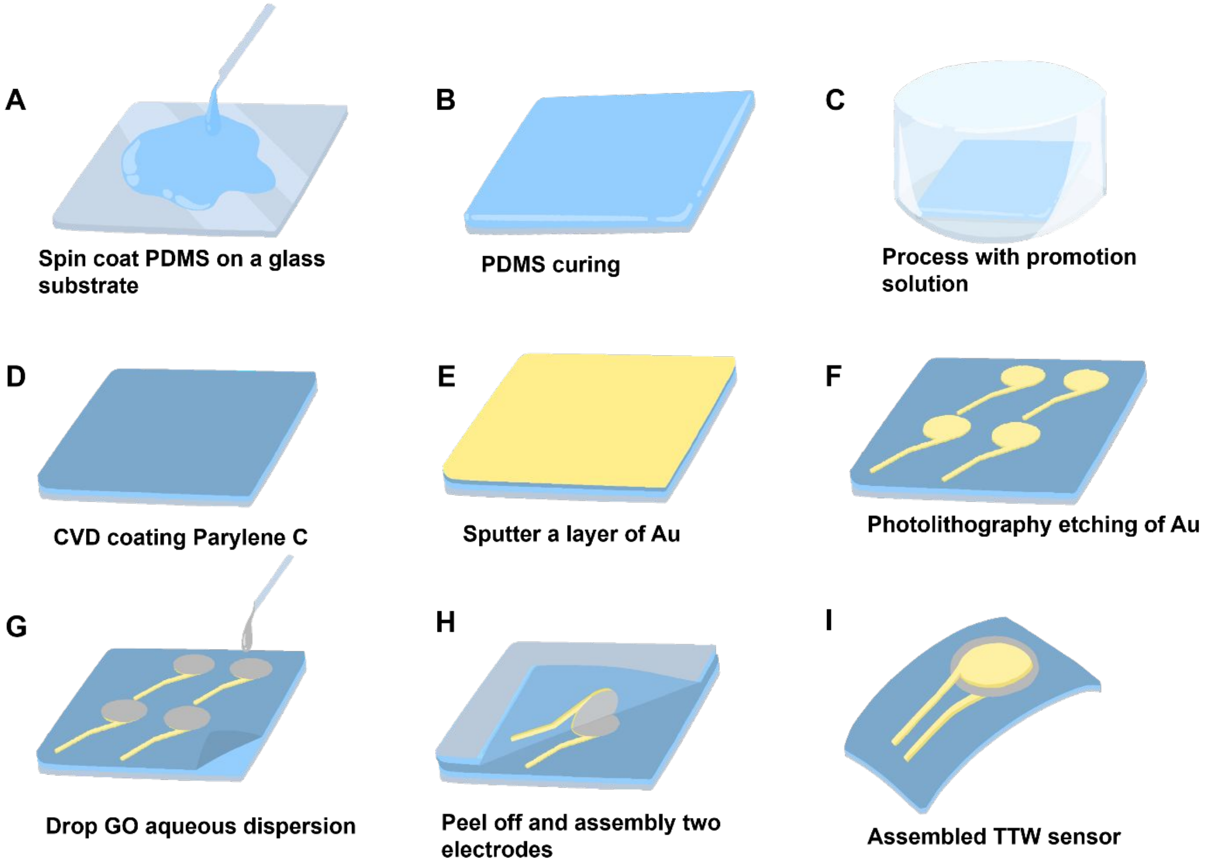

Figure S1. Fabrication process of the tactile-transparent wearable sensor.

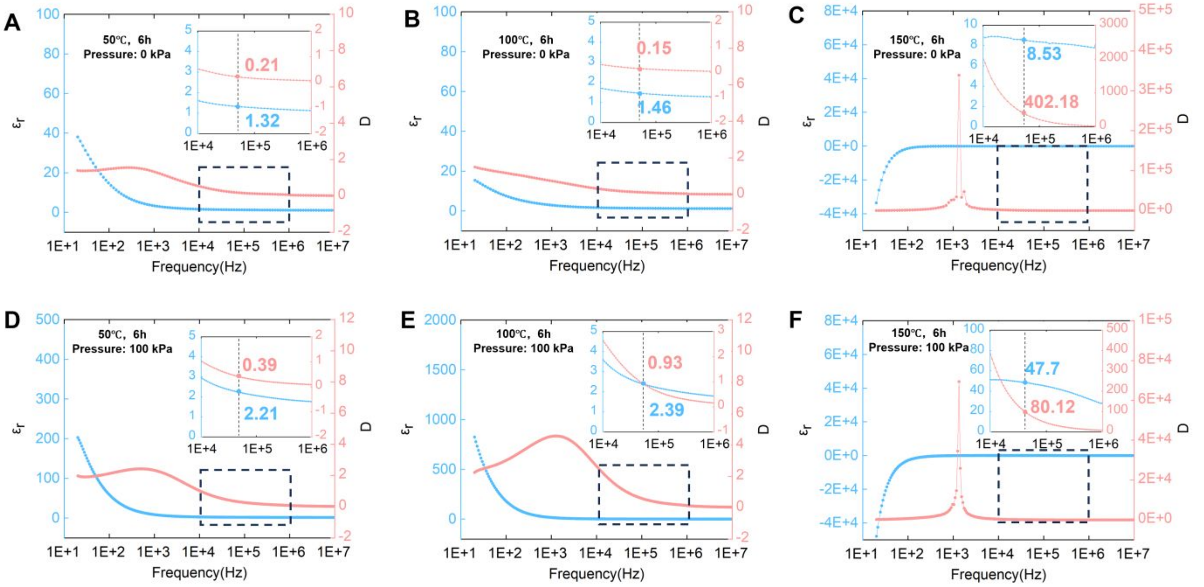

Figure S2. Dielectric spectroscopy of paper-like graphene oxide film. A-C) Dielectric spectroscopy of GO film at different drying temperatures across a frequency range of 10 Hz-10 MHz without pressure. The inset shows the close-up views of frequency from 10^4 to 10^6 Hz. D-F) Dielectric spectroscopy of GO film at different drying temperatures across a frequency range of 10 Hz-10 MHz with 100 kPa pressure. The inset shows the close-up views of frequency from 10^4 to 10^6 Hz.

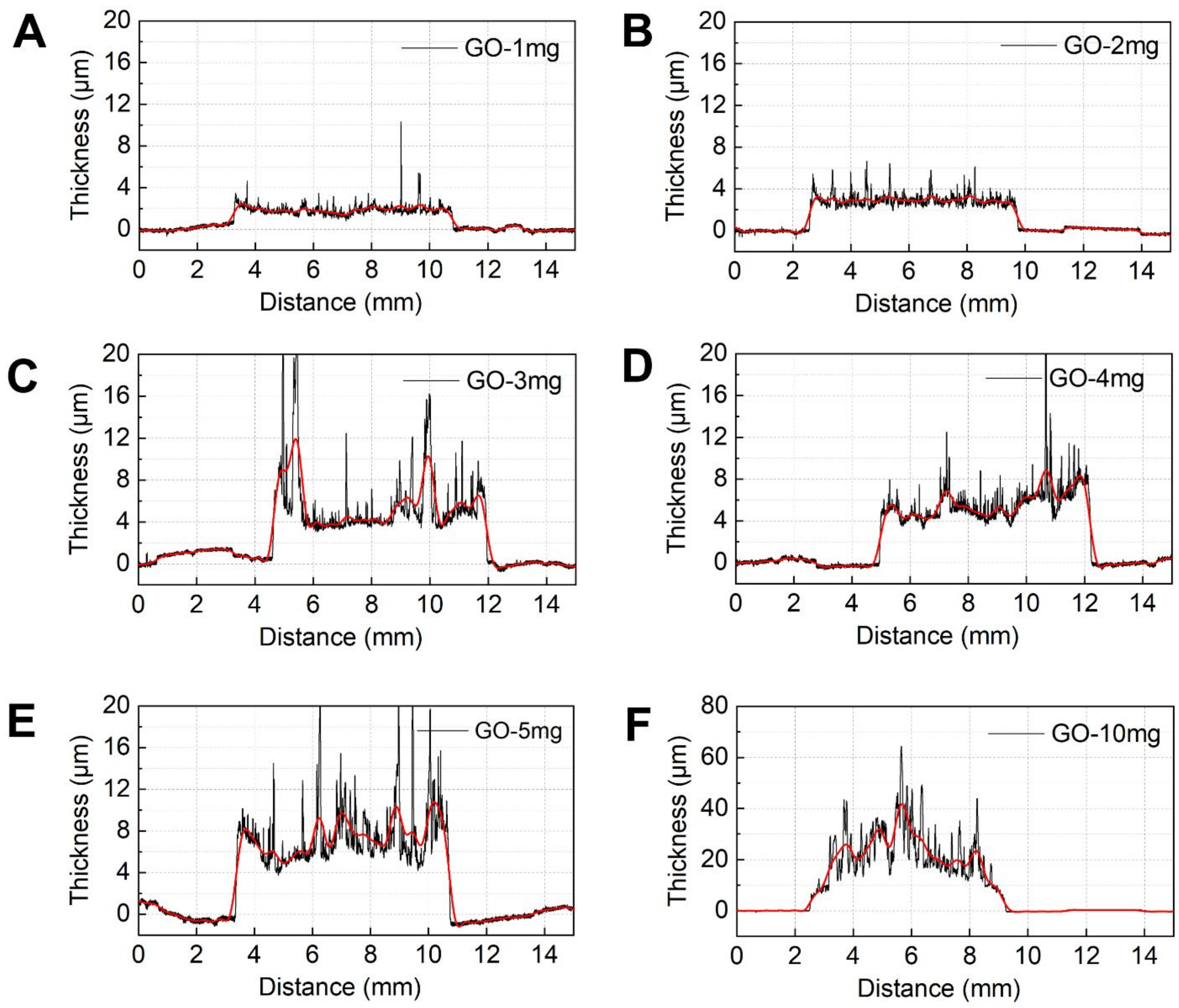

Figure S3. The influence of GO aqueous dispersions concentration on the thickness and surface flatness properties of the film, formed at a temperature of 50℃. A-F) Thickness and surface flatness of paper-thin GO films obtained from GO aqueous dispersions with different concentrations, from 1 mg/ml to 10 mg/ml.

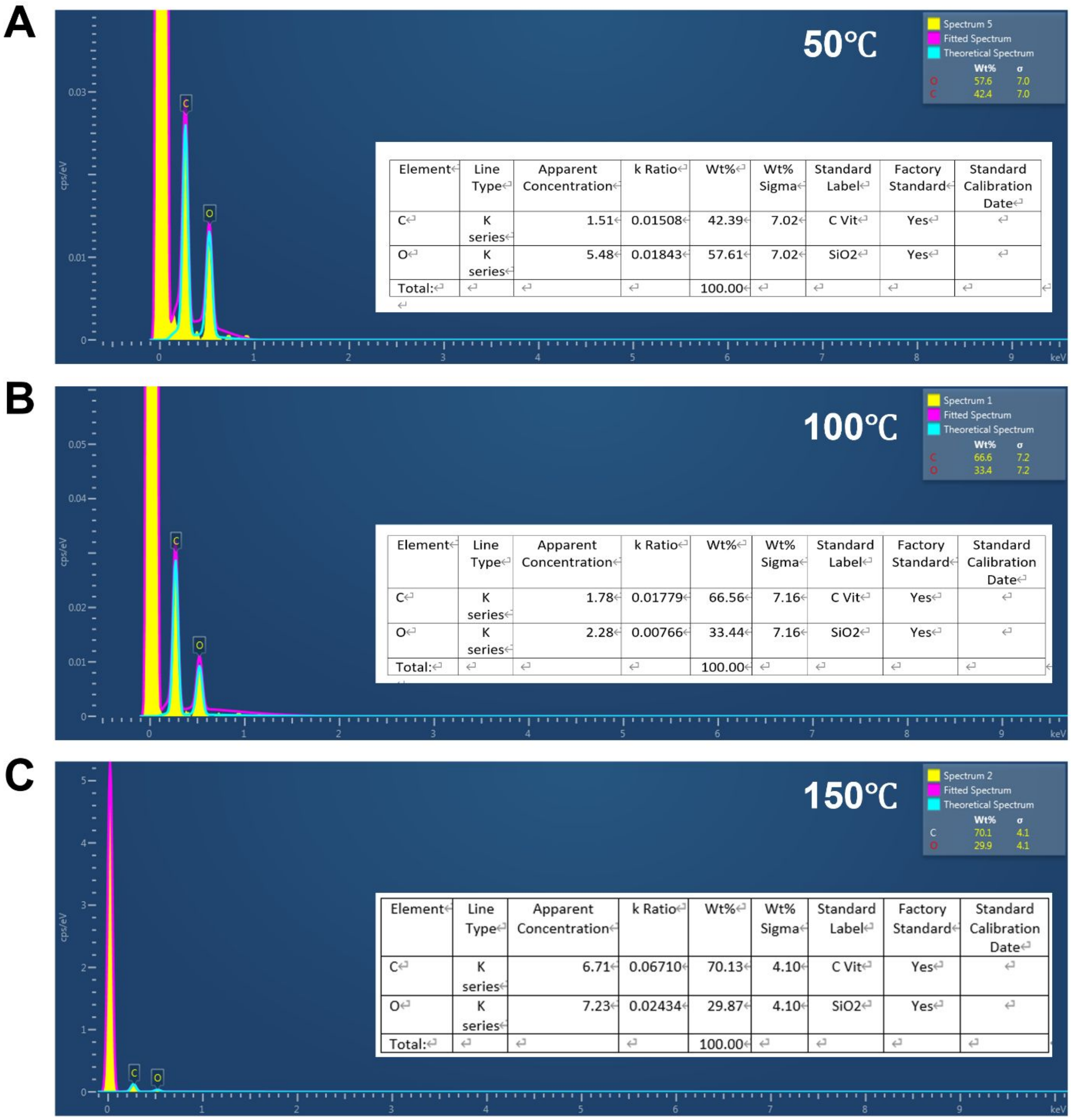

Figure S4. Energy-dispersive X-ray (EDX) spectroscopies of paper-like GO film at different temperatures. (A) 50 °C. (B) 100 °C. (C) 150 °C.

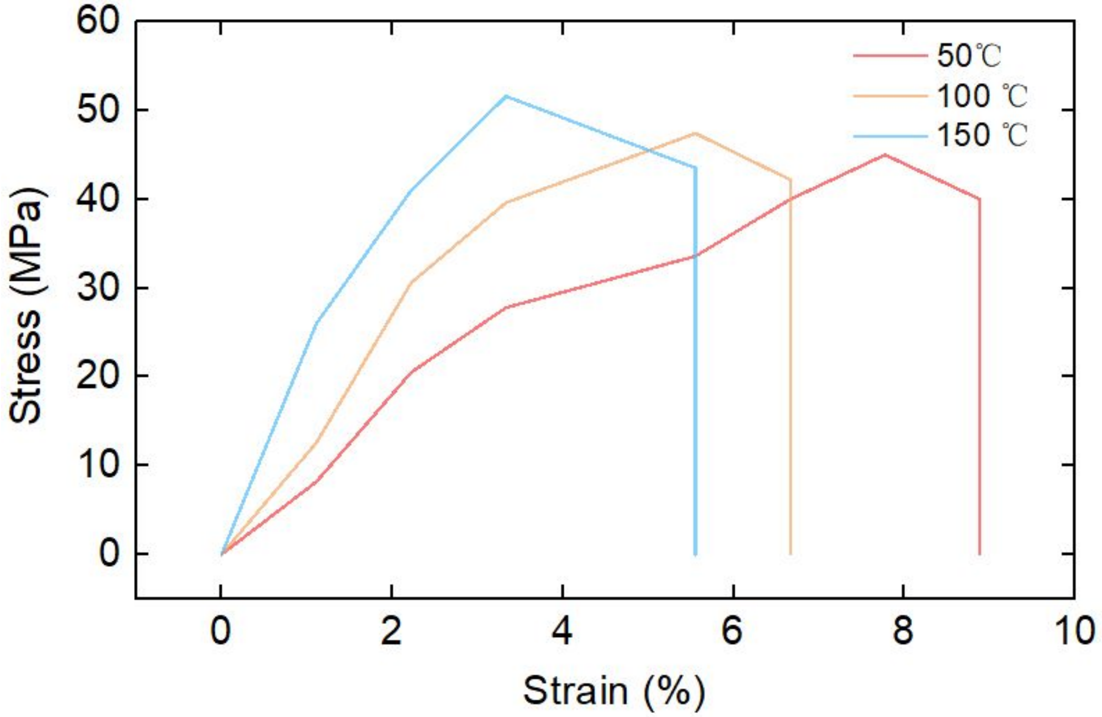

Figure S5. Tensile test results of thin paper-like graphene oxide films at different annealing temperatures.

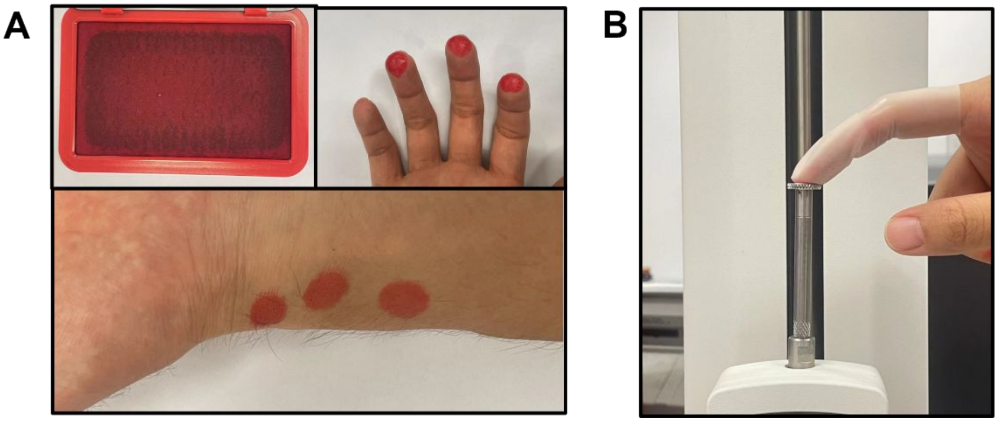

Figure S6. Fingertip pressure test for physicians obtaining pulse waves. A) Measurement of contact area during fingertip force application. B) Fingertip force measurement.

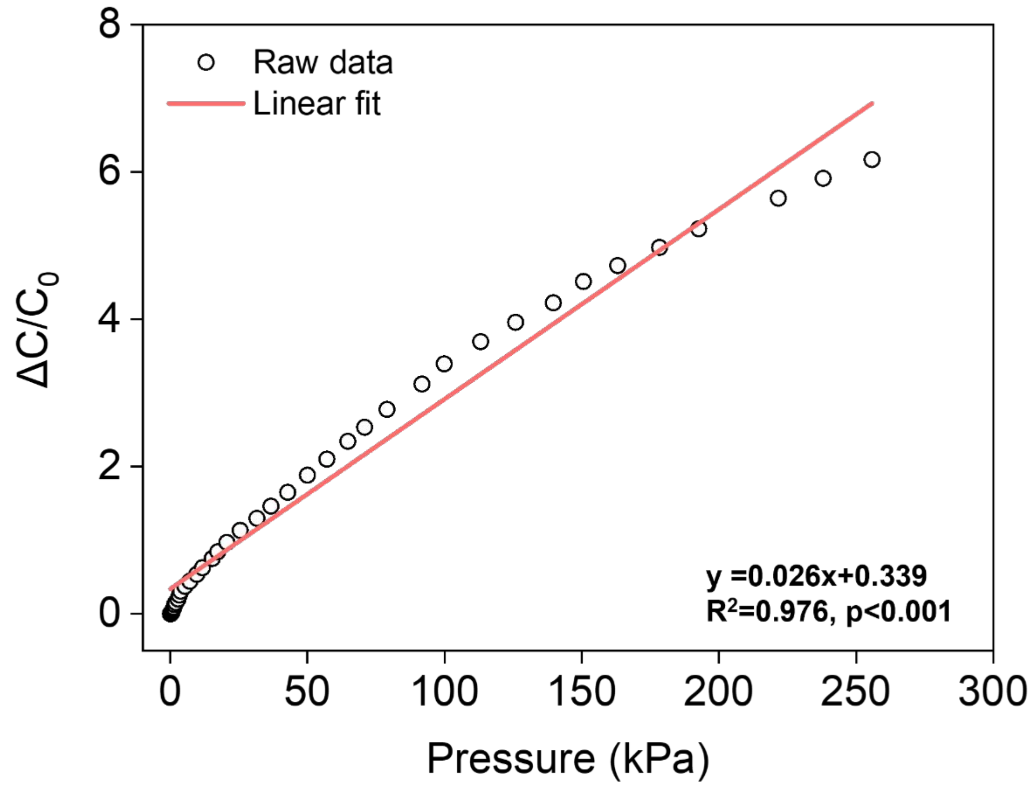

Figure S7. Correlation coefficient between pressure and capacitance variation.

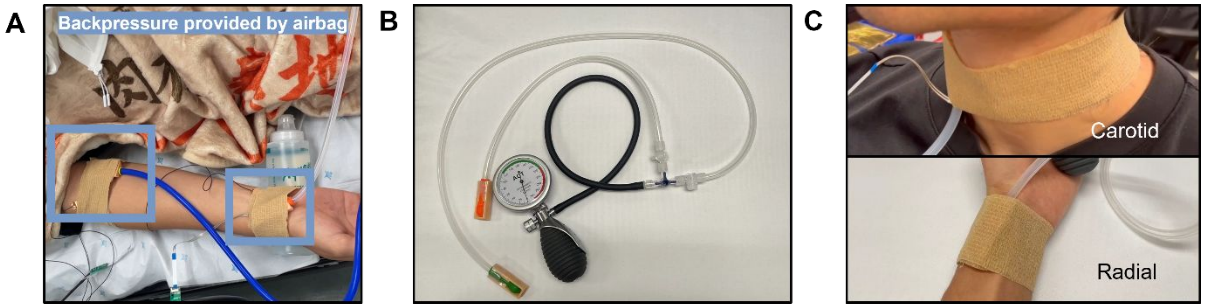

Figure S8. Airbag system for applying pressure at the PWV test sites. A) Experimental setup for brachial-radial PWV acquisition using an airbag. B) The optical image of the airbag system. C) Images of the experimental setup using an airbag system to apply pressure to the radial and carotid arteries.

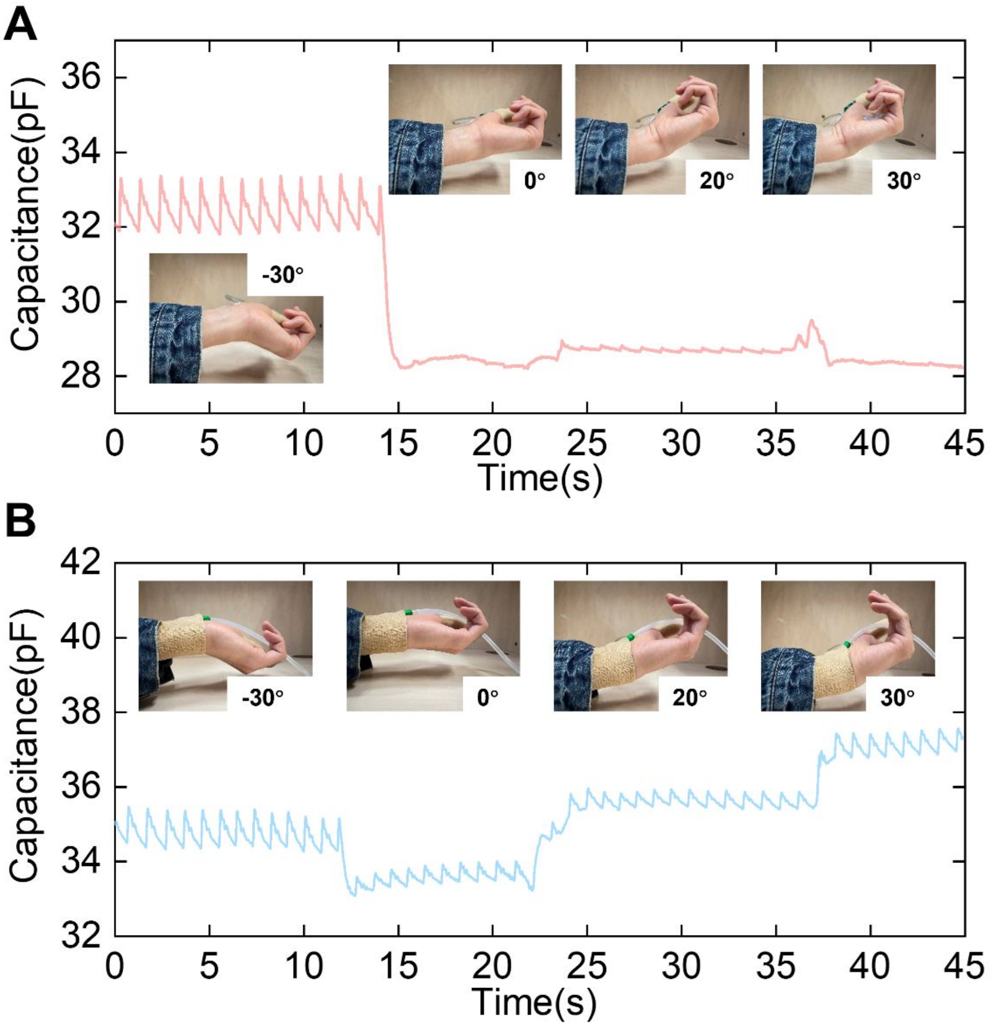

Figure S9. Validation of the stability of a customized airbag-enhanced sensor-skin interface. A) Wrist pulse wave results captured by the TTW sensor at different bending angles when pressurized without an airbag. B) Wrist pulse wave results captured by the TTW sensor at different bending angles when pressurized with an airbag.

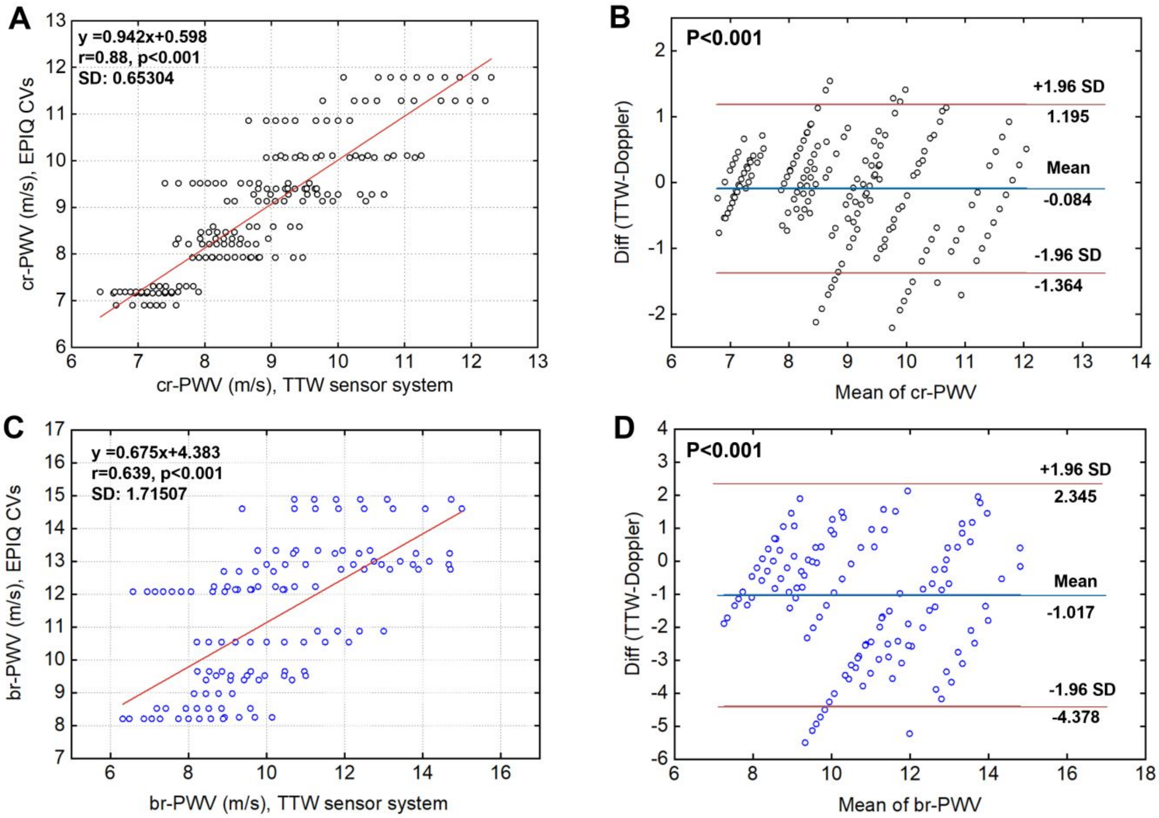

Figure S10. Pearson's correlation plots and Bland-Altman plots. A) Pearson's correlation of cr-PWV measurements. B) Bland-Altman plot of cr-PWV measurements. C) Pearson's correlation of br-PWV measurements. D) Bland-Altman plot of br-PWV measurements.

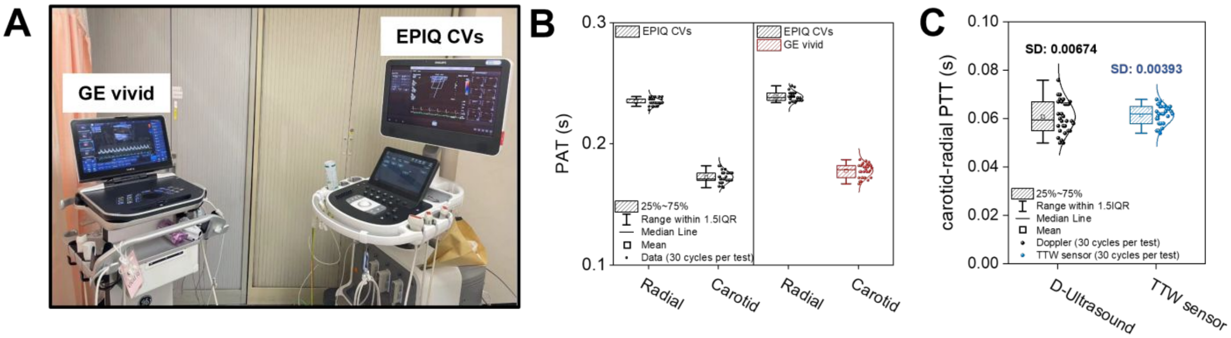

Figure S11. Measurement of PWV based on Doppler ultrasound. A) Optical image of the two devices used for Doppler ultrasound measurement of PWV. B) Scatterplot of PAT results from measurements made by the single-device-based method and the two-device-based method. C) Scatterplot of carotid-radial PTT results based on measurements from the two-device-based method and the TTW sensor system. D-Ultrasound: Two ultrasound devices collect data synchronously. n = 30 pulse cycles data points in the box plots of (B and C). Square, mean; center line, median; box limits, upper and lower quartiles; whiskers, 1.5 × interquartile range; points, data points, in box plots of (B and C).

242

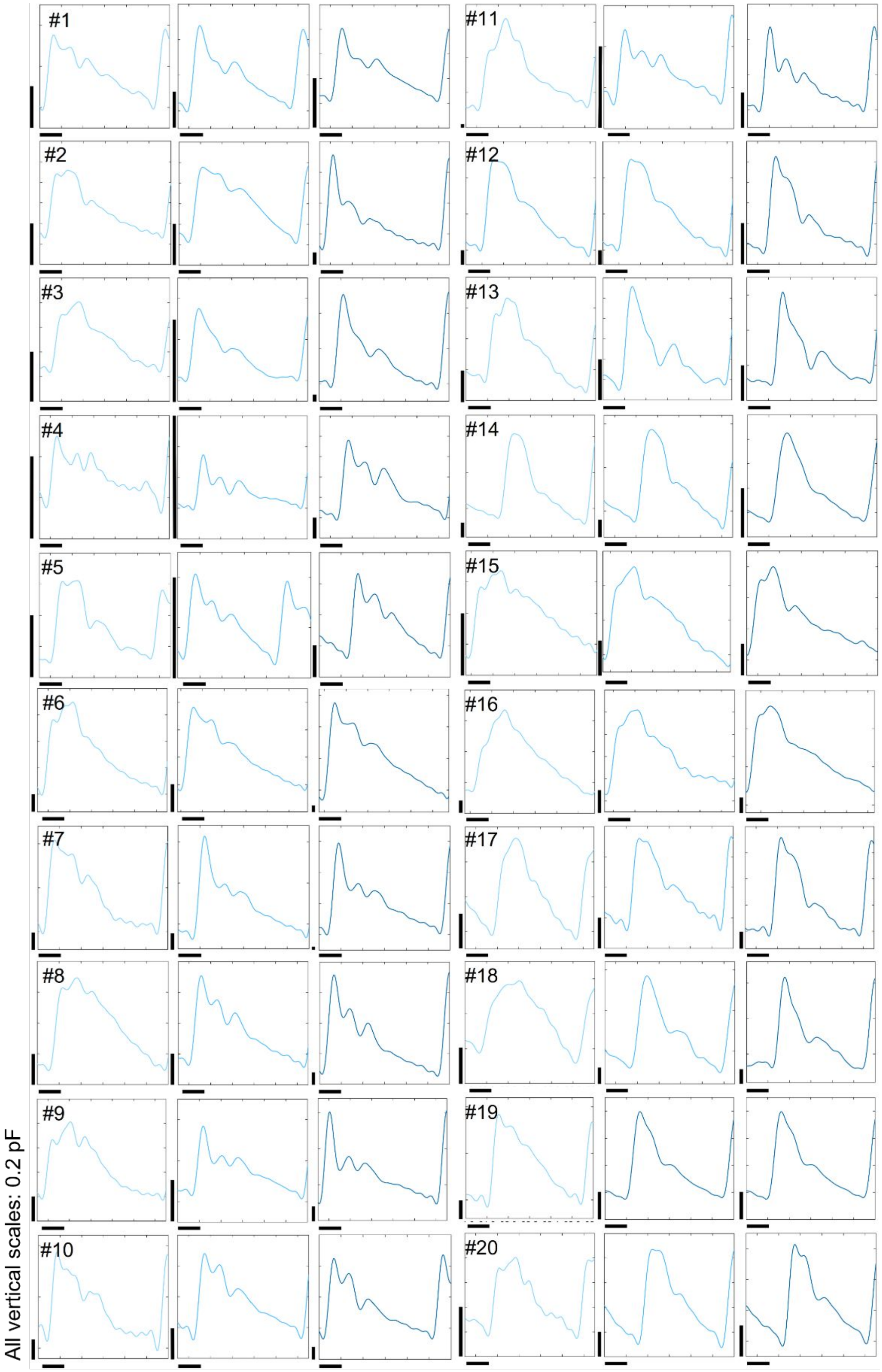

243 **Figure S12.** From left to right are the carotid, brachial, and radial pulse waveforms of 20 subjects participating
244 in the PWV tests.

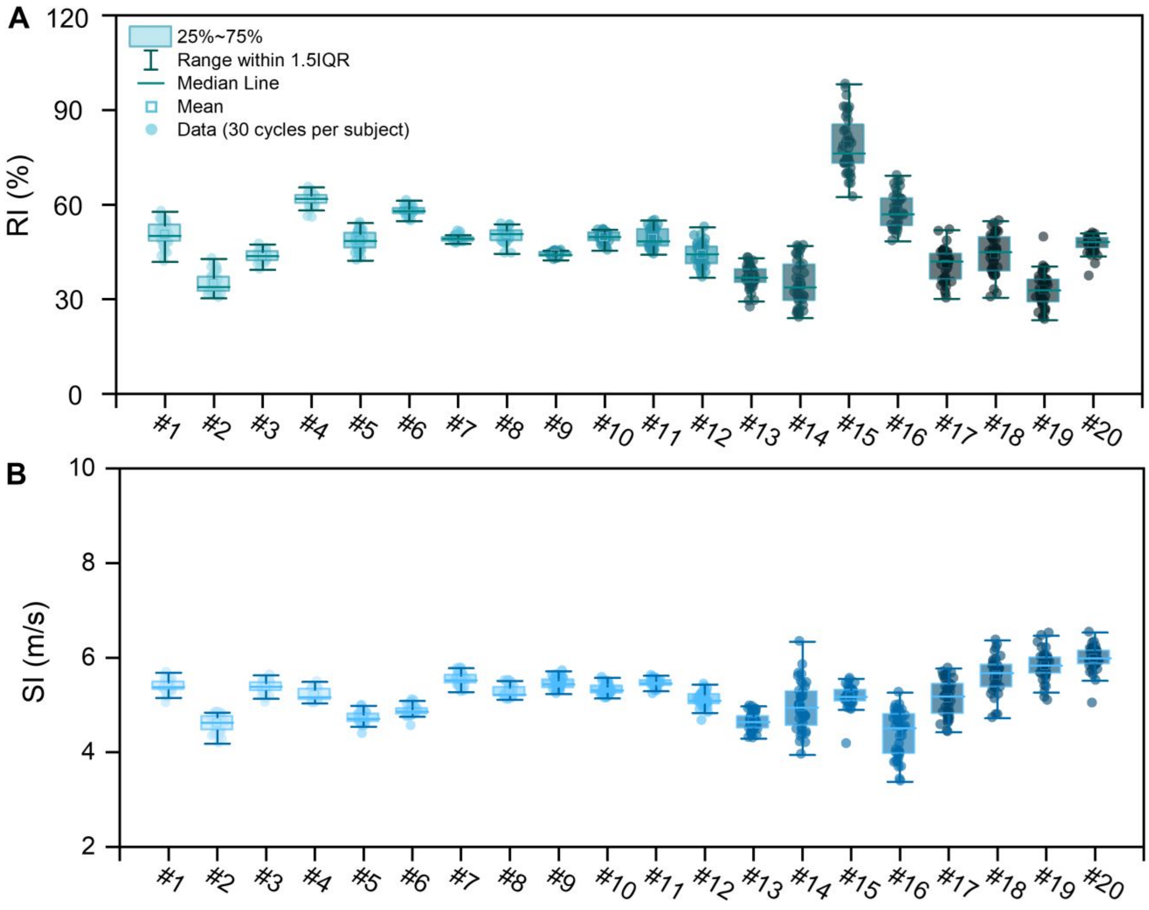

Figure S13. Distribution of cardiovascular risk assessment indicators on the radial artery in 20 subjects. A) Reflection index. B) Stiffness index. n = 30 pulse cycles data points in the box plots of (A and B). Square, mean; center line, median; box limits, upper and lower quartiles; whiskers, 1.5 × interquartile range; points, data points.

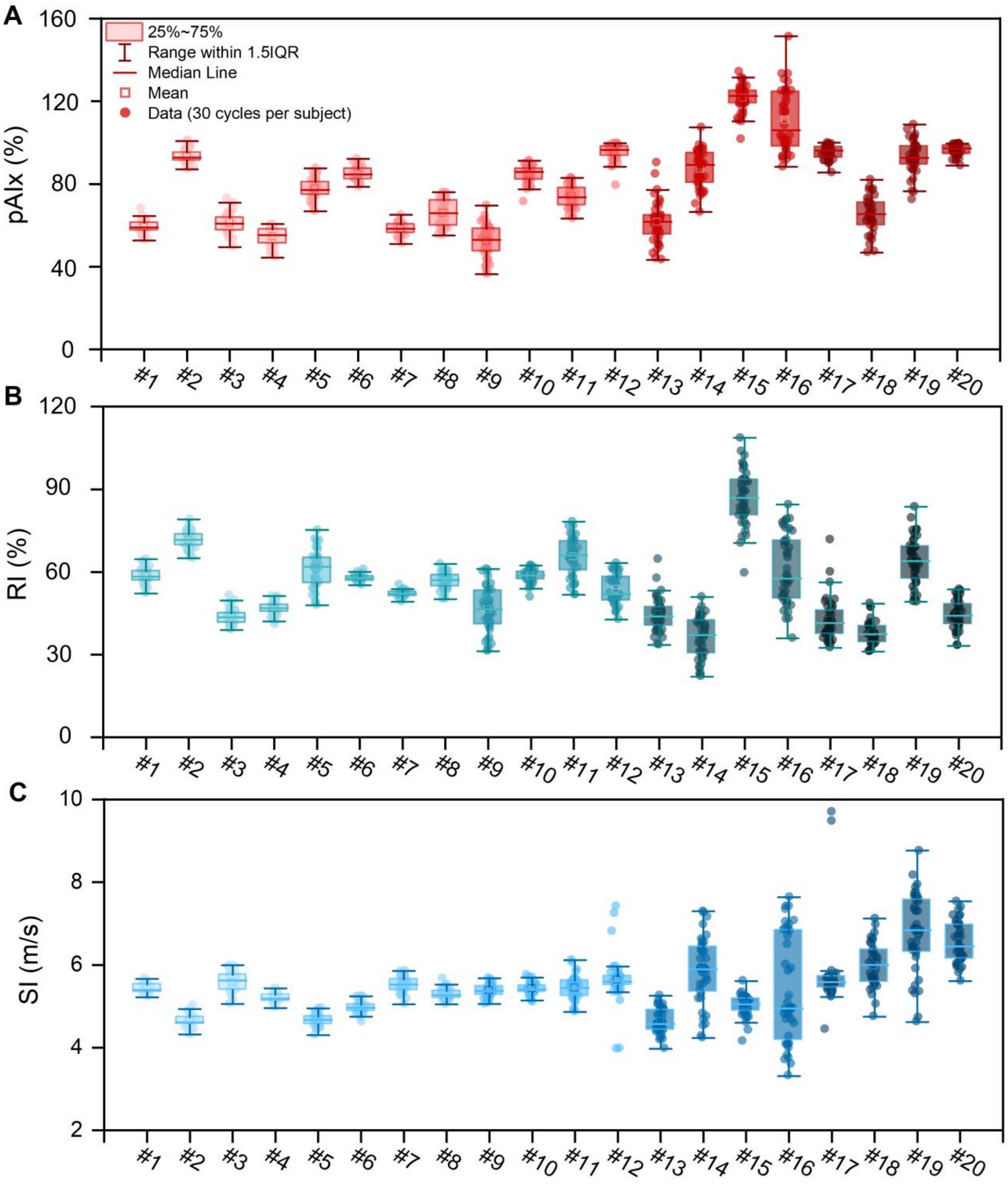

Figure S14. Distribution of cardiovascular risk assessment indicators on the brachial artery in 20 subjects. A) Peripheral augmentation index. B) Reflection index. C) Stiffness index. n = 30 pulse cycles data points in the box plots of (A, B, and C). Square, mean; center line, median; box limits, upper and lower quartiles; whiskers, 1.5 × interquartile range; points, data points.

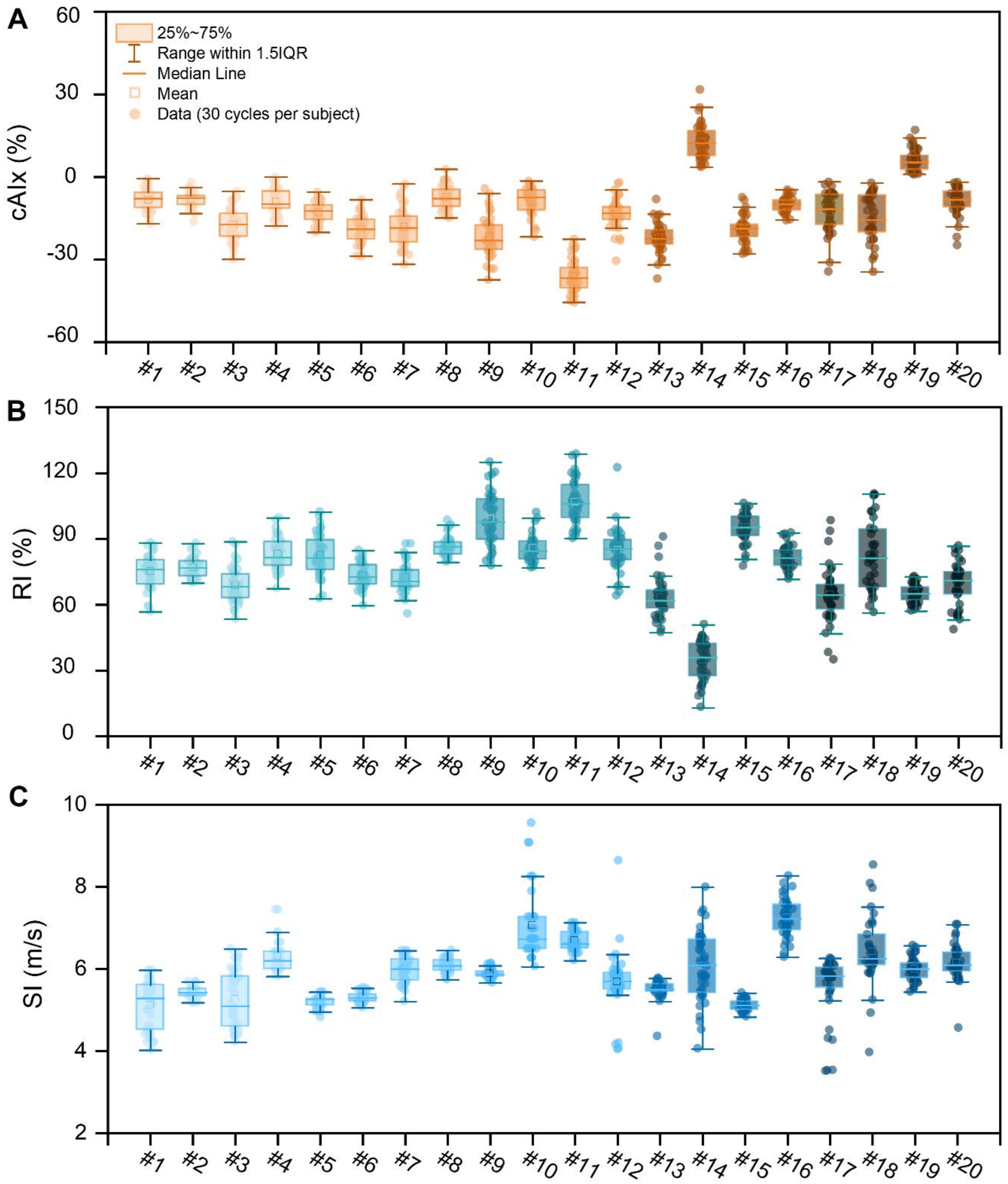

Figure S15. Distribution of cardiovascular risk assessment indicators on the carotid artery in 20 subjects. A) Central augmentation index. B) Reflection index. C) Stiffness index. n = 30 pulse cycles data points in the box plots of (A, B, and C). Square, mean; center line, median; box limits, upper and lower quartiles; whiskers, 1.5 × interquartile range; points, data points.

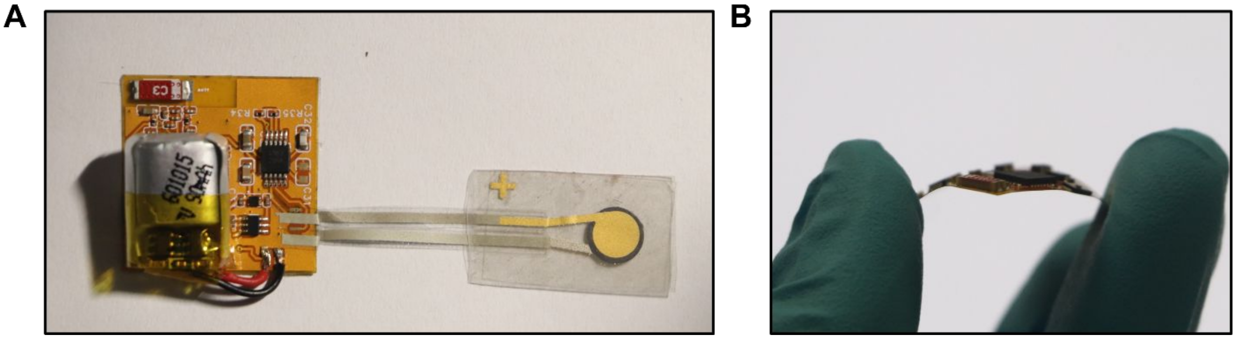

Figure S16. Optical images of the TTW sensor system. (a). Top view of the fPCB and optical images of the TTW sensor. (b) fPCB under bending.

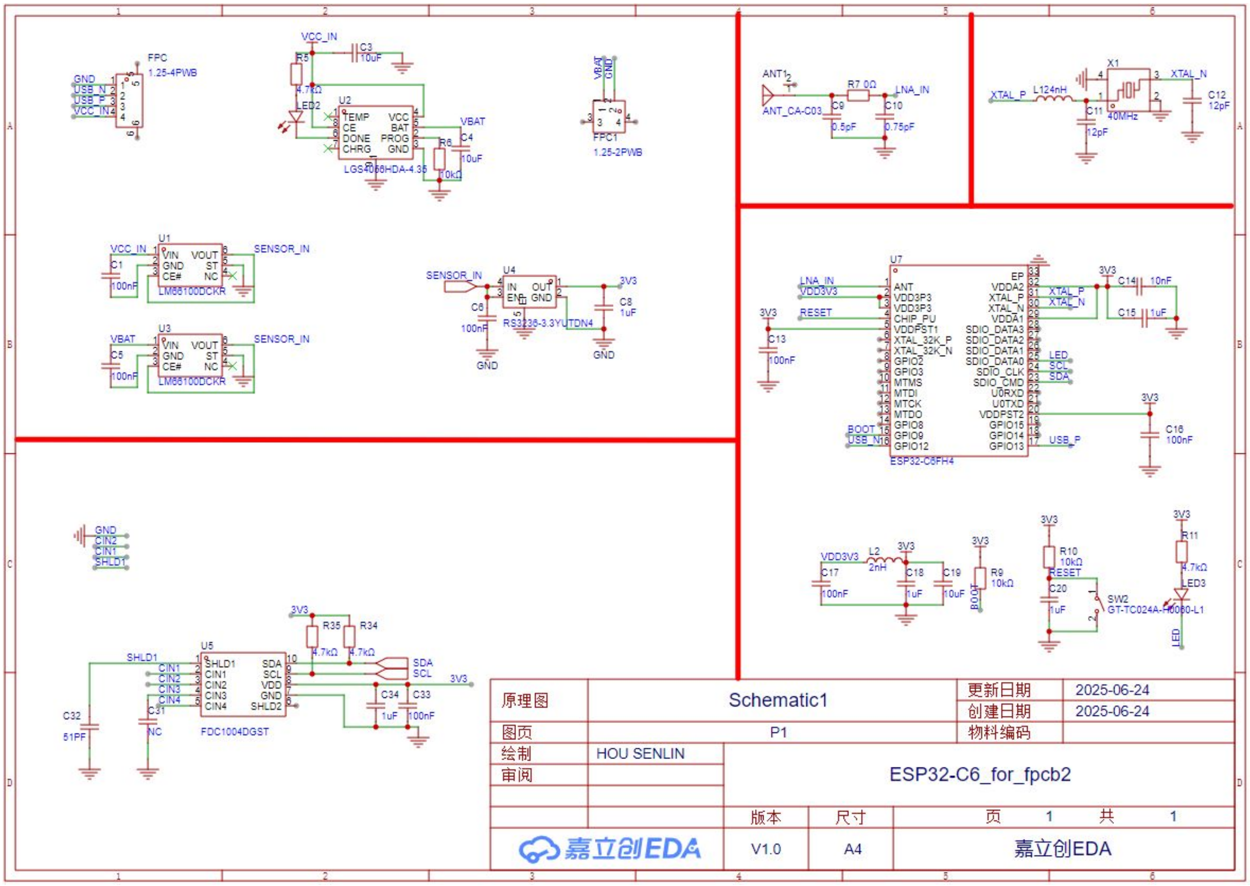

Figure S17. Schematic design of the signal sampling module.

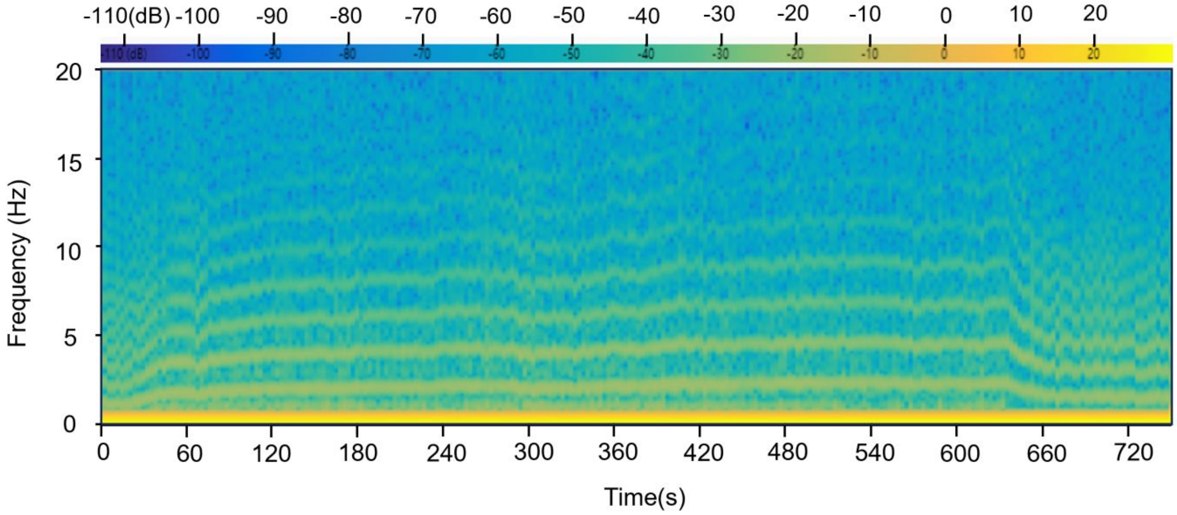

Figure S18. Continuous wavelets transform radial pulse waves during the cycling test.

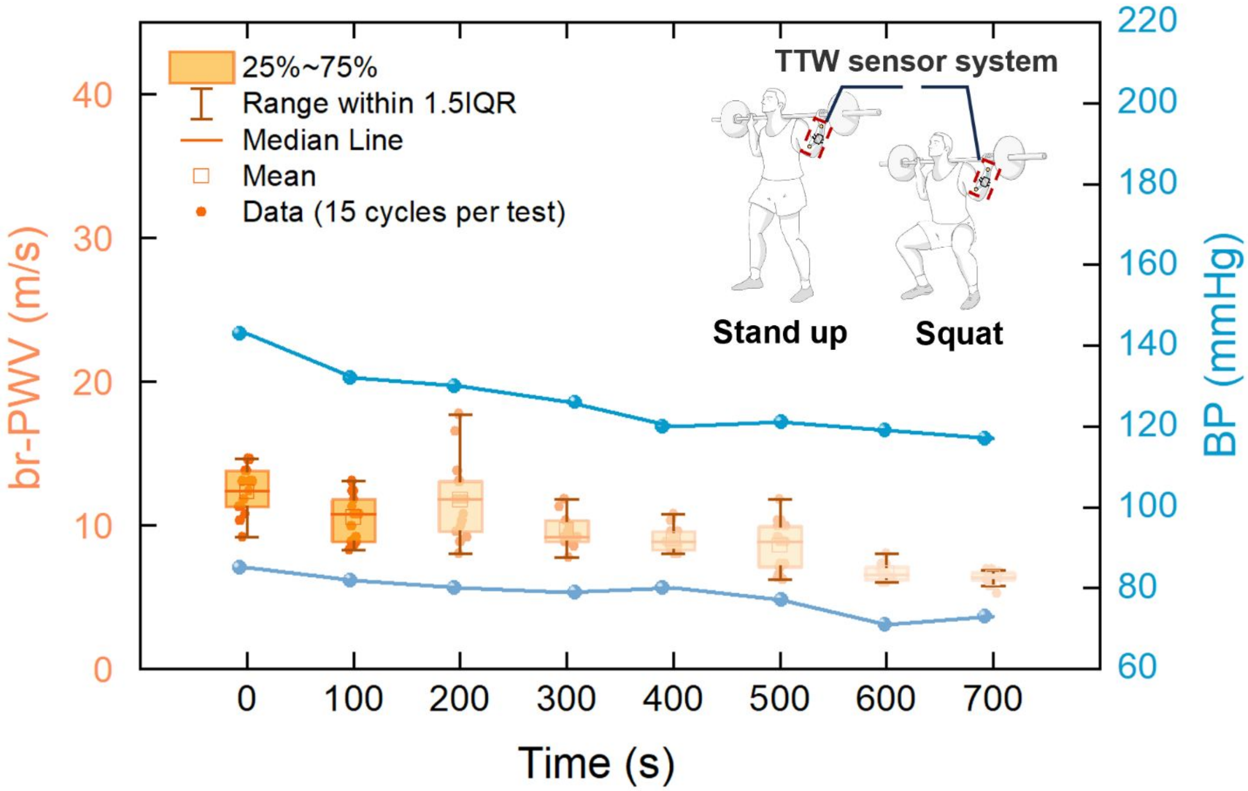

Figure S19. Relationship between blood pressure and brachial-radial pulse wave velocity after high-intensity anaerobic exercise. $n = 15$ pulse cycles data points in the box plot. Square, mean; center line, median; box limits, upper and lower quartiles; whiskers, 1.5 × interquartile range; points, data points.

Table S1. Technical comparison of continuous cardiovascular monitoring.

Sensing Mechanism	Dimension	Versatile (PWV, RI, BP, etc.)	Continuous & Dynamic	Wearable	Detect limit	Endurance	Ref
Photoplethysmography	90 mm × 25 mm × 10 mm	BP<br>PWV<br>CO<br>SVR	20 mins bike	Skin adhesion (3 position)<br>Bluetooth transmission	-	-	12
Photoplethysmography	Diameter: 38 mm<br>Thickness: 13 mm	BP<br>Temperature	-	Wristband (1 position)<br>Bluetooth transmission	-	-	44
Ultrasound	11.5 mm × 2.5 mm (element: 0.175 mm × 2.5 mm, thickness: 0.1 mm)	BP	-	Skin adhesion (1 position)<br>Oscilloscope (DSO6032A)	-	-	24
Ultrasound	3 × 3 elements (element :1.5 mm × 1.5 mm, thickness: 0.1 mm)	RI	-	Skin adhesion (3 position)<br>Oscilloscope (PXI-5922)	-	500 bending cycles	25
Piezoelectric	3 mm × 3 mm × 0.2 mm	BP<br>PWV	-	Wristband (1 position)<br>Bluetooth transmission	-	2500 cycles<br>(50 mN)	21
Piezoelectric	18 mm × 18 mm × 0.1 mm	BP	-	Wristband (2 position)<br>Bluetooth transmission	-	50,000 cycles<br>(10 kPa)	31
Piezoelectric	Diameter: 3 mm	BP<br>PWV	-	Skin adhesion (2 position)<br>Bluetooth transmission	Pulse amplitude:<br>3.1 mV	-	32
**Capacitive**	**15 mm × 8 mm × 0.15 mm**	**BP<br>PWV<br>cAI<br>pAI<br>RI<br>SI**	**10 mins cycling and squatting**	**Skin adhesion (5 positions)<br>Bluetooth transmission**	**10 mg**	**10000 cycles<br>(1-20 kPa)**	**Our work**

Table S2. Volunteer information.

No.	Age	Gender (Male/Female)	Height (cm)	Weight (kg)	BMI (kg/m^2)	SBP (mmHg)	DBP (mmHg)	Average HR (Beats/min)
1	27	Female	168	56	19.84	106	73	59
2	28	Female	160	51.8	20.23	113	78	66
3	26	Male	175	75	24.48	112	73	65
4	28	Male	169	88.1	30.84	127	83	59
5	28	Female	153	46	19.65	113	77	68
6	29	Female	160	53	20.70	106	73	51
7	28	Male	181	65	19.84	122	75	56
8	22	Female	164	55	20.44	108	69	56
9	29	Male	175	71	23.18	112	65	62
10	32	Male	165	62.5	22.95	115	73	57
11	32	Male	173	75	25.06	105	63	55
12	48	Female	158	52.5	21.03	104	62	59
13	52	Female	166	70	25.40	105	72	60
14	50	Female	159	46	18.19	108	71	74
15	60	Female	166	63	22.86	131	83	52
16	53	Female	152	45.3	19.60	114	72	53
17	60	Female	165	65	23.87	100	65	72
18	50	Male	168	95	33.65	115	78	66
19	52	Male	170	65	22.49	107	68	61
20	54	Male	162	63	24.00	123	87	72

References

(1) Zhang, K.; Yan, Y.; Wang, Z.; Ma, G.; Jia, D.; Huang, X.; Zhou, Y. Integration of Electrical Properties and Polarization Loss Modulation on Atomic Fe–N-Rgo for Boosting Electromagnetic Wave Absorption. *Nano-Micro Lett.* **2025**, *17*, 46.

(2) Wu, Y.; Zhao, X.; Li, F.; Fan, Z. Evaluation of Mixing Rules for Dielectric Constants of Composite Dielectrics by Mc-Fem Calculation on 3d Cubic Lattice. *J. Electroceram.* **2003**, *11*, 227-239.

(3) Salomão, F. C.; Lanzoni, E. M.; Costa, C. A.; Deneke, C.; Barros, E. B. Determination of High-Frequency Dielectric Constant and Surface Potential of Graphene Oxide and Influence of Humidity by Kelvin Probe Force Microscopy. *Langmuir* **2015**, *31*, 11339-11343.

(4) Grogan, T.; Sanchez-Gonzalez, M. A.; Illyés, M.; Rizvi, S. A. Noninvasive Central Hemodynamic Monitoring in the Primary Care Setting: Improving Prevention and Management of Cardiovascular Diseases. *J. Clin. Transl. Res.* **2023**, *9*, 175–181.

(5) Chen, Z.; Peng, B.; Zhou, Y.; Hao, Y.; Xie, X. Interpretable and Accurate Curve‐Fitting Method for Arterial Pulse Wave Modeling and Decomposition. *Int. J. Numer. Methods Biomed. Eng.* **2023**, *39*, e3775.

(6) Pingali, U.; Nutalapati, C.; Gundagani, S. Effect of Omega-3 Fatty Acid Alone and in Combination with Proprietary Chromium Complex on Endothelial Function in Subjects with Metabolic Syndrome: A Randomized, Double-Blind, Parallel-Group Clinical Study. *Evidence-Based Complementary and Alternative Medicine* **2021**, *2021*, 2972610.

(7) Wu, H.-T.; Chen, J.-J. Calculation of an Improved Stiffness Index Using Decomposed Radial Pulse and Digital Volume Pulse Signals. *J. Pers. Med.* **2022**, *12*, 1768.

(8) Charlton, P. H.; Paliakaitė, B.; Pilt, K.; Bachler, M.; Zanelli, S.; Kulin, D.; Allen, J.; Hallab, M.; Bianchini, E.; Mayer, C. C.; Terentes-Printzios, D.; Dittrich, V.; Hametner, B.; Veerasingam, D.; Žikić, D.; Marozas, V. Assessing Hemodynamics from the Photoplethysmogram to Gain Insights into Vascular Age: A Review from Vascagenet. *American Journal of Physiology-Heart and Circulatory Physiology* **2022**, *322*, H493-H522.
