# Supplementary material for: Tactile-Transparent Wearable Sensor for Clinician-Friendly Pulse Wave Velocity Monitoring and Cardiovascular Risk Profiling
Source: ACS Nano. 2025 Sep 5;19(36):32822–35. doi: 10.1021/acsnano.5c11375 (PMC12444991; doi:10.1021/acsnano.5c11375)
Supplement: Supplementary file 4 [file nn5c11375_si_004.pdf]

The University of Hong Kong

Hospital Authority Hong Kong West Cluster

Institutional Review Board of The University of Hong Kong/Hospital Authority Hong Kong West Cluster (HKU/HA HKW IRB)

Room 901, Administration Block, Queen [illegible]ary Hospital, 102 Pokfulam Road, Hong Kong
Tel: (852) 2255 408[illegible] [illegible]ax: (852) 2255 4735

HKU/HA HKW IRB is an independent committee established by The University of Hong Kong and Hospital Authority Hong Kong West Cluster and authorized to perform ethics and scientific review and oversight of clinical studies in accordance with its standard operating procedure and the principles of the Declaration of Helsinki and ICH Good Clinical Practice.

Date: 21 Nov 2024 IRB/REC Ref. No.: UW 19-490

To: Prof. JG Shen
School of Chinese Medicine
The University of Hong Kong

This notice is issued by HKU/HA HKW IRB with respect to the application/submission by you, being the principal investigator of the following study at your study site:

- Study Protocol Title: Artificial Intelligence Recognitions for Traditional Chinese Medicine Pulse Patterns and its Correlation Study with Body Constitution Types in Healthy Human Subjects
- Study Protocol No.: N/A
- Coordinating Investigator (if applicable): N/A
- Study Site: Queen Mary Hospital

In accordance with our standard operating procedure, we have duly performed ethics and scientific review of your application/submission as detailed below:

- Nature of Your Application/Submission: ☐ Initial application ☒ Amendments/changes ☒ Others: Progress Report
- Mode of Review: ☐ Full review ☒ Expedited review
  ☐ Harmonized review for across-territories multicenter study
  ☐ Primary review (by <Select full/expedited> review)
  ☐ Secondary review, with <Select full/expedited> primary review by: <Select IRB/REC> (see attached approval notice)
- Date of Review/Decision: 19 Nov 2024
- Document(s) Reviewed: See Schedule 1
- Reviewer(s): See Schedule 2

After due review by our reviewer(s), we hereby write to inform you of our decision on your application/submission as follows:

- Decision:
  ☒ Application approved (Amendments/changes)
  ☒ Receipt of submission acknowledged without comment (Others)
  ☐ Application disapproved (see opinion(s) below)
  ☐ Others (see opinion(s) below)
- Opinion(s) (if applicable): N/A

- Regular Progress Report(s) Required: Every 12 months from the date of initial approval and during the period of the study

You, being the principal investigator and undertaking the ultimate responsibility for the conduct of the study at your study site, are reminded to comply with our requirements and to maintain communication with us during the period of the study by undertaking the principal investigator's responsibilities including (but not limited to):

- supervising your study team and ensuring compliance with the study protocol and all applicable requirements;
- if the study is an industry-sponsored clinical study, submitting to us a copy of the fully executed indemnity agreement satisfying the Hospital Authority's requirement prior to commencement of the study (if it has not been submitted yet);
- observing and complying with all applicable requirements under our standard operating procedure ("IRB/REC SOP"), the Declaration of Helsinki and the ICH GCP (if applicable);
- submitting regular progress report(s) at the required intervals (as specified above) in accordance with the requirements in the IRB/REC SOP;
- not implementing any amendment/change to any approved study document/material without our written approval, except where necessary to eliminate any immediate hazard to the participants or if an amendment/change is only of an administrative or logistical nature;
- notifying us of any new information that may adversely affect the rights, safety or well-being of the participants or the proper conduct of the study;
- reporting any deviation from the study protocol or compliance incident that has occurred during the study and may adversely affect the rights, safety or well-being of any participant in accordance with the requirements in the IRB/REC SOP;
- submitting safety reports on all SAEs observed at your study site or SUSARs reported from outside your study site in accordance with the requirements in the IRB/REC SOP; and
- submitting a final report in accordance with the requirements in the IRB/REC SOP upon completion or termination of the study at your study site.

For the avoidance of doubt, this notice only serves to inform you of our decision from our ethics and scientific evaluation and applies only to the study at the specified study site. It does not release you from your obligation to comply with other applicable management, regulatory and ethics requirements including (but not limited to):

- obtaining the necessary consent from the management of your institution/department in accordance with the requirements of your institution/department;
- if required by Hong Kong laws or regulations, obtaining a certificate for clinical trial through the Hong Kong Department of Health and complying with the associated requirements;
- if required by applicable laws or regulations or relevant organizations or institutions, obtaining other necessary approval(s) or permission(s) (whether local, national or overseas, or of ethical, regulatory, legal or other natures) relevant to the conduct of the study.

Yours sincerely,
for and on behalf of
HKU/HA HKW IRB

Ms. Jenny Ng
HKU/HA HKW IRB Secretary

Schedule 1
Documents Reviewed

The documents reviewed by HKU/HA HKW IRB with respect to the said application/submission include:

01. Protocol Amendment Application Form dated 1 November 2024 (Addition of Co-Investigators - Prof. Wong Chun Ka and Prof. Yap Yat Hin and Addition of Study Site - Queen Mary Hospital)
02. Proposed Research Project; Version 4.0 dated 08 October 2024
03. Informed Consent Form; Version 5.0 dated 2024 04 25 (Chinese)
04. Poster; Version 2.0 dated 2024-10-08 (Chinese)
05. Approval Letter from Shenzhen Bao'an Pure Chinese Medicine Treatment Hospital Research Ethics Committee; Ref: LL-HY-2024-0205 dated 1 March 2024
06. Short CV of Co-Investigators
07. Research Progress Report Form dated 9 September 2024

Schedule 2
List of Reviewers

The reviewers participated in reviewing the said application/submission and making the decision on behalf of HKU/HA HKW IRB include:

Name	Occupation & Affiliated Organization	Gender (M/F)	Membership Category (Mark "Y" as appropriate)		
			Scientific	Non-scientific	Independent
Prof. Ernest Ng	Clinical Professor, Obstetrics & Gynaecology, HKU	M	✓		
