# Supplementary material for: Tactile-Transparent Wearable Sensor for Clinician-Friendly Pulse Wave Velocity Monitoring and Cardiovascular Risk Profiling
Source: ACS Nano. 2025 Sep 5;19(36):32822–35. doi: 10.1021/acsnano.5c11375 (PMC12444991; doi:10.1021/acsnano.5c11375)
Supplement: Supplementary file 5 [file nn5c11375_si_005.pdf]

Informed Consent Form

Study Title: Artificial Intelligence Recognitions for Traditional Chinese Medicine Pulse Patterns and its Correlation Study with Body Constitution Types

Version Number: Version 4.0 2023.08.17

Study Site: School of Chinese Medicine, The University of Hong Kong

Principal Investigator: Professor Shen Jiangang

We are inviting you to participate in a research study. Before you decide whether to participate, it is important that you understand the reasons for conducting this study and the procedures involved. Please read the following information carefully. If you have any questions, please ask the Traditional Chinese Medicine (TCM) practitioner or researcher in charge of this study before signing this consent form.

About This Study

The purpose of our study is to investigate the artificial intelligence recognitions of TCM pulse patterns and its correlation with body constitution types.

What Will I Do If I Participate?

Participants will be divided into two groups based on their physical condition:

1. Control Group: Individuals with no chronic diseases lasting for 3 months or more; currently not using any medication.
2. Case Group: Individuals diagnosed by Western medicine with cardiovascular diseases, metabolic syndrome, diabetes, hypertension, hyperlipidemia, or obesity.

You will undergo pulse diagnosis by both an AI-based pulse diagnosis instrument and an experienced TCM practitioner. You will also be asked to complete the TCM Constitution Questionnaire, the TCM Five-visceral Health Status Questionnaire, and the Perceived Stress Scale. According to the study protocol, you may be arranged to receive complimentary examinations including an electrocardiogram (ECG), quantitative echocardiogram, arterial stiffness measurement, and body composition analysis.

What Are the Benefits of Participating in the Study?

You will be provided with a free pulse diagnosis and TCM constitution diagnosis. If you undergo the complimentary electrocardiogram, quantitative echocardiogram, arterial stiffness measurement, and body composition analysis, you will receive the relevant reports.

What If Something Goes Wrong?

We do not anticipate any harm resulting from your participation in this study. In the unlikely event of an injury resulting from your participation, medical services will be provided. There are no special arrangements for compensation. If you are harmed due to negligence, you may have grounds for legal action. In any case, if you wish to complain about the treatment you received during the study, we will provide you with information about the complaint mechanism for health

services.

Will My Participation in This Study Be Kept Confidential?
As a research subject, all information about you will be kept strictly confidential. Your name or personal identity will not be used for any public purpose, publication, or transmitted outside of the medical center. In accordance with the laws of the Hong Kong Special Administrative Region, particularly the Personal Data (Privacy) Ordinance, you have the right to the protection of your confidential personal data, such as any of your personal data collected, held, stored, managed, controlled, used (including for analysis or comparison), transmitted into or out of Hong Kong, not disclosed, erased, or otherwise handled or destroyed in this study. By signing this consent form to participate, you expressly authorize the Principal Investigator, their research team, and the Institutional Review Board of the University of Hong Kong/Hong Kong West Cluster, Hospital Authority (HKU/HA HKW IRB) to use and retain your personal data for the purposes and under the circumstances described in this consent form. If you have any questions, you should consult the Privacy Commissioner for Personal Data or their office (telephone number: 2255-4086) for proper supervision or guidance on the protection of your personal data to ensure you are fully aware of and understand the importance of complying with the laws governing privacy data.

Upon completion of the project, volunteers will receive an allowance of HKD 500 to compensate for the time, inconvenience, transportation, and other expenses incurred by participating in this study.

By signing and dating below, I agree that:

1. I confirm that I have read and understood the contents of the above research consent form and have been given the opportunity to ask questions.
2. I understand that authorized personnel from regulatory bodies (The Institutional Review Board of the University of Hong Kong/Hong Kong West Cluster, Hospital Authority) may inspect my records related to my participation in this study. I grant them access to my records.
3. I agree to participate in the above research project.

Participant ID:
Study Title: Artificial Intelligence Recognitions for Traditional Chinese Medicine Pulse Patterns and its Correlation Study with Body Constitution Types
Principal Investigator: Professor Shen Jiangang

____________________	____________________	________________
Participant's Signature	Participant's Name	Date

____________________	____________________	________________
Researcher's Signature	Researcher's Name	Date

知情同意書

研究名稱：中醫脈象的人工智能識別及其與體質類型的相關性研究

版本編號：Version 4.0 2023.08.17

研究地點：香港大學中醫藥學院

研究醫生：沈劍剛教授

我們邀請您參加一項研究。決定是否參與研究計劃前，請您務必明白進行這項研究的原因和程式。請仔細閱讀以下內容，如有任何疑問，簽署同意書之前請先向負責此項研究的中醫醫生或研究人員查詢。

關於此項研究

我們研究的目的是探究中醫脈象的人工智能識別及其與體質類型的相關性。

如果我參加將會做什麼?

參加者將按照身體情況分成兩組:

1.對照組：無持續3個月或以上的慢性疾病；目前没有使用任何藥物。

2.病例組：經西醫診斷為心血管疾病、代謝綜合症、糖尿病、高血壓、高血脂症或肥胖。

您將分別接受基於人工智能的脈診儀檢測和有經驗中醫師的脈診檢查，並填寫中醫體質量表、中醫五臟健康量表和壓力知覺量表。根據研究方案，您可能被安排接受免費的心電圖、定量超聲心動圖、動脈僵硬度和身體成分檢查。

參加研究有什麼益處?

您將被提供免費的脈診和中醫體質診斷。如果您接受了免費的心電圖、定量超聲心動圖、動脈僵硬度和身體成分檢查，您將獲得相關報告。

如果出現錯誤?

我們不預期任何因參與本研究帶來的傷害。萬一因參與研究引致損傷，我們會提供醫療服務。沒有特別的補償安排。如果是由於疏忽而導致您受到損害，您可以有理由訴諸法律。無論如何，如果您想投訴在研究過程中得到的待遇，我們會提供有關健康服務投訴機制給您。

我參與本研究會被保密嗎？

作為研究對象，所有關於您的資料會絕對保密。您的姓名或您的個人身分不會被利用作任何公共用途、出版、或傳送到醫療中心之外。根據香港特別行政區執行法例，尤其是個人資料（隱私）條例，您享有或可享有權利保護保密個人資料，例如在這項研究中收集、保管、保存、管理、控制、使用（包括分析或比較）、進出香港的傳輸，不洩露、刪除或以任何方式處理或銷毀任何您的個人資料。 在本同意書上簽署參加，您明確授權給研究醫生、其研究小組和香港大學及醫管局港島西醫院聯網研究倫理委員會使用、保存您的個人資料，並將會如本同意書的目的和所述情況使用。如果您有任何疑問，您應該徵詢個人資料私隱專員或其辦公室（電話號碼：2255-4086），關於妥善監控或指導您對個人資料的保障，以確保您完全知悉及明白到遵守規管私隱資料法例的重要性。

完成項目後，志願者將獲發500港幣津貼以補償參加此項研究所付出之時間、所造成之不便及所需的交通費用或其他開支。

在以下簽署並註明日期，我同意：

1. 我在此證實，我已閱讀及明白上述研究同意書的內容，並獲得了提問的機會。
2. 我明白監管機構（香港大學及醫管局港島西醫院聯網研究倫理委員會）委派的人員可能會查核有關我參與此項研究的記錄。我允許他們可以獲得我的記錄。
3. 我同意參加以上研究項目。

參加者編號：

研究題目：中醫脈象的人工智能識別及其與體質類型的相關性研究

研究醫生：**沈劍剛教授**

陈毅龙	陈毅龙	2024-07-26
參加者簽署	參加者姓名	日期
陈小同	陈小同	2024-07-26
研究人員簽署	研究人員姓名	日期

知情同意書

研究名稱：中醫脈象的人工智能識別及其與體質類型的相關性研究

版本編號：Version 4.0 2023.08.17

研究地點：香港大學中醫藥學院

研究醫生：沈劍剛教授

我們邀請您參加一項研究。決定是否參與研究計劃前，請您務必明白進行這項研究的原因和程式。請仔細閱讀以下內容，如有任何疑問，簽署同意書之前請先向負責此項研究的中醫醫生或研究人員查詢。

關於此項研究

我們研究的目的是探究中醫脈象的人工智能識別及其與體質類型的相關性。

如果我參加將會做什麼？

參加者將按照身體情況分成兩組：

1. 對照組：無持續3個月或以上的慢性疾病；目前没有使用任何藥物。

2. 病例組：經西醫診斷為心血管疾病、代謝綜合症、糖尿病、高血壓、高血脂症或肥胖。

您將分別接受基於人工智能的脈診儀檢測和有經驗中醫師的脈診檢查，並填寫中醫體質量表、中醫五臟健康量表和壓力知覺量表。根據研究方案，您可能被安排接受免費的心電圖、定量超聲心動圖、動脈僵硬度和身體成分檢查。

參加研究有什麼益處？

您將被提供免費的脈診和中醫體質診斷。如果您接受了免費的心電圖、定量超聲心動圖、動脈僵硬度和身體成分檢查，您將獲得相關報告。

如果出現錯誤？

我們不預期任何因參與本研究帶來的傷害。萬一因參與研究引致損傷，我們會提供醫療服務。沒有特別的補償安排。如果是由於疏忽而導致您受到損害，您可以有理由訴諸法律。無論如何，如果您想投訴在研究過程中得到的待遇，我們會提供有關健康服務投訴機制給您。

我參與本研究會被保密嗎？

作為研究對象，所有關於您的資料會絕對保密。您的姓名或您的個人身分不會被利用作任何公共用途、出版、或傳送到醫療中心之外。根據香港特別行政區執行法例，尤其是個人資料（隱私）條例，您享有或可享有權利保護保密個人資料，例如在這項研究中收集、保管、保存、管理、控制、使用（包括分析或比較）、進出香港的傳輸，不洩露、刪除或以任何方式處理或銷毀任何您的個人資料。 在本同意書上簽署參加，您明確授權給研究醫生、其研究小組和香港大學及醫管局港島西醫院聯網研究倫理委員會使用、保存您的個人資料，並將會如本同意書的目的和所述情況使用。如果您有任何疑問，您應該徵詢個人資料私隱專員或其辦公室（電話號碼：2255-4086），關於妥善監控或指導您對個人資料的保障，以確保您完全知悉及明白到遵守規管私隱資料法例的重要性。

完成項目後，志願者將獲發500港幣津貼以補償參加此項研究所付出之時間、所造成之不便及所需的交通費用或其他開支。

在以下簽署並註明日期，我同意：

1. 我在此證實，我已閱讀及明白上述研究同意書的內容，並獲得了提問的機會。
2. 我明白監管機構（香港大學及醫管局港島西醫院聯網研究倫理委員會）委派的人員可能會查核有關我參與此項研究的記錄。我允許他們可以獲得我的記錄。
3. 我同意參加以上研究項目。

參加者編號：

研究題目：中醫脈象的人工智能識別及其與體質類型的相關性研究

研究醫生：**沈劍剛教授**

Peijian Lin	林培坚	2024-07-19
參加者簽署	參加者姓名	日期
[illegible] 陈小同	陈小同	2024-07-19
研究人員簽署	研究人員姓名	日期

知情同意書

研究名稱：中醫脈象的人工智能識別及其與體質類型的相關性研究

版本編號：Version 4.0 2023.08.17

研究地點：香港大學中醫藥學院

研究醫生：沈劍剛教授

我們邀請您參加一項研究。決定是否參與研究計劃前，請您務必明白進行這項研究的原因和程式。請仔細閱讀以下內容，如有任何疑問，簽署同意書之前請先向負責此項研究的中醫醫生或研究人員查詢。

關於此項研究

我們研究的目的是探究中醫脈象的人工智能識別及其與體質類型的相關性。

如果我參加將會做什麼？

參加者將按照身體情況分成兩組：

1.對照組：無持續3個月或以上的慢性疾病；目前没有使用任何藥物。

2.病例組：經西醫診斷為心血管疾病、代謝綜合症、糖尿病、高血壓、高血脂症或肥胖。

您將分別接受基於人工智能的脈診儀檢測和有經驗中醫師的脈診檢查，並填寫中醫體質量表、中醫五臟健康量表和壓力知覺量表。根據研究方案，您可能被安排接受免費的心電圖、定量超聲心動圖、動脈僵硬度和身體成分檢查。

參加研究有什麼益處？

您將被提供免費的脈診和中醫體質診斷。如果您接受了免費的心電圖、定量超聲心動圖、動脈僵硬度和身體成分檢查，您將獲得相關報告。

如果出現錯誤？

我們不預期任何因參與本研究帶來的傷害。萬一因參與研究引致損傷，我們會提供醫療服務。沒有特別的補償安排。如果是由於疏忽而導致您受到損害，您可以有理由訴諸法律。無論如何，如果您想投訴在研究過程中得到的待遇，我們會提供有關健康服務投訴機制給您。

我參與本研究會被保密嗎？

作為研究對象，所有關於您的資料會絕對保密。您的姓名或您的個人身分不會被利用作任何公共用途、出版、或傳送到醫療中心之外。根據香港特別行政區執行法例，尤其是個人資料（隱私）條例，您享有或可享有權利保護保密個人資料，例如在這項研究中收集、保管、保存、管理、控制、使用（包括分析或比較）、進出香港的傳輸，不洩露、刪除或以任何方式處理或銷毀任何您的個人資料。 在本同意書上簽署參加，您明確授權給研究醫生、其研究小組和香港大學及醫管局港島西醫院聯網研究倫理委員會使用、保存您的個人資料，並將會如本同意書的目的和所述情況使用。如果您有任何疑問，您應該徵詢個人資料私隱專員或其辦公室（電話號碼：2255-4086），關於妥善監控或指導您對個人資料的保障，以確保您完全知悉及明白到遵守規管私隱資料法例的重要性。

完成項目後，志願者將獲發500港幣津貼以補償參加此項研究所付出之時間、所造成之不便及所需的交通費用或其他開支。

在以下簽署並註明日期，我同意：

1. 我在此證實，我已閱讀及明白上述研究同意書的內容，並獲得了提問的機會。
2. 我明白監管機構（香港大學及醫管局港島西醫院聯網研究倫理委員會）委派的人員可能會查核有關我參與此項研究的記錄。我允許他們可以獲得我的記錄。
3. 我同意參加以上研究項目。

參加者編號：

研究題目：中醫脈象的人工智能識別及其與體質類型的相關性研究

研究醫生：**沈劍剛教授**

刘庆	**刘庆**	**2024-07-15**
參加者簽署	參加者姓名	日期
陈小同	**陈小同**	**2024-07-15**
研究人員簽署	研究人員姓名	日期

知情同意書

研究名稱：中醫脈象的人工智能識別及其與體質類型的相關性研究

版本編號：Version 4.0 2023.08.17

研究地點：香港大學中醫藥學院

研究醫生：沈劍剛教授

我們邀請您參加一項研究。決定是否參與研究計劃前，請您務必明白進行這項研究的原因和程式。請仔細閱讀以下內容，如有任何疑問，簽署同意書之前請先向負責此項研究的中醫醫生或研究人員查詢。

關於此項研究

我們研究的目的是探究中醫脈象的人工智能識別及其與體質類型的相關性。

如果我參加將會做什麼？

參加者將按照身體情況分成兩組：

1.對照組：無持續3個月或以上的慢性疾病；目前没有使用任何藥物。

2.病例組：經西醫診斷為心血管疾病、代謝綜合症、糖尿病、高血壓、高血脂症或肥胖。

您將分別接受基於人工智能的脈診儀檢測和有經驗中醫師的脈診檢查，並填寫中醫體質量表、中醫五臟健康量表和壓力知覺量表。根據研究方案，您可能被安排接受免費的心電圖、定量超聲心動圖、動脈僵硬度和身體成分檢查。

參加研究有什麼益處？

您將被提供免費的脈診和中醫體質診斷。如果您接受了免費的心電圖、定量超聲心動圖、動脈僵硬度和身體成分檢查，您將獲得相關報告。

如果出現錯誤？

我們不預期任何因參與本研究帶來的傷害。萬一因參與研究引致損傷，我們會提供醫療服務。沒有特別的補償安排。如果是由於疏忽而導致您受到損害，您可以有理由訴諸法律。無論如何，如果您想投訴在研究過程中得到的待遇，我們會提供有關健康服務投訴機制給您。

我參與本研究會被保密嗎?

作為研究對象，所有關於您的資料會絕對保密。您的姓名或您的個人身分不會被利用作任何公共用途、出版、或傳送到醫療中心之外。根據香港特別行政區執行法例，尤其是個人資料（隱私）條例，您享有或可享有權利保護保密個人資料，例如在這項研究中收集、保管、保存、管理、控制、使用（包括分析或比較）、進出香港的傳輸，不洩露、刪除或以任何方式處理或銷毀任何您的個人資料。 在本同意書上簽署參加，您明確授權給研究醫生、其研究小組和香港大學及醫管局港島西醫院聯網研究倫理委員會使用、保存您的個人資料，並將會如本同意書的目的和所述情況使用。如果您有任何疑問，您應該徵詢個人資料私隱專員或其辦公室（電話號碼：2255-4086），關於妥善監控或指導您對個人資料的保障，以確保您完全知悉及明白到遵守規管私隱資料法例的重要性。

完成項目後，志願者將獲發500港幣津貼以補償參加此項研究所付出之時間、所造成之不便及所需的交通費用或其他開支。

在以下簽署並註明日期，我同意：

1. 我在此證實，我已閱讀及明白上述研究同意書的內容，並獲得了提問的機會。
2. 我明白監管機構（香港大學及醫管局港島西醫院聯網研究倫理委員會）委派的人員可能會查核有關我參與此項研究的記錄。我允許他們可以獲得我的記錄。
3. 我同意參加以上研究項目。

參加者編號:

研究題目：中醫脈象的人工智能識別及其與體質類型的相關性研究

研究醫生：**沈劍剛教授**

刘希望	刘希望	2024-07-26
參加者簽署	參加者姓名	日期
陈小同	陈小同	2024-07-26
研究人員簽署	研究人員姓名	日期

知情同意書

研究名稱：中醫脈象的人工智能識別及其與體質類型的相關性研究

版本編號：Version 4.0 2023.08.17

研究地點：香港大學中醫藥學院

研究醫生：沈劍剛教授

我們邀請您參加一項研究。決定是否参與研究計劃前，請您務必明白進行這項研究的原因和程式。請仔細閱讀以下內容，如有任何疑問，簽署同意書之前請先向負責此項研究的中醫醫生或研究人員查詢。

關於此項研究

我們研究的目的是探究中醫脈象的人工智能識別及其與體質類型的相關性。

如果我參加將會做什麼？

參加者將按照身體情況分成兩組：

1.對照組：無持續3個月或以上的慢性疾病；目前没有使用任何藥物。

2.病例組：經西醫診斷為心血管疾病、代謝綜合症、糖尿病、高血壓、高血脂症或肥胖。

您將分別接受基於人工智能的脈診儀檢測和有經驗中醫師的脈診檢查，並填寫中醫體質量表、中醫五臟健康量表和壓力知覺量表。根據研究方案，您可能被安排接受免費的心電圖、定量超聲心動圖、動脈僵硬度和身體成分檢查。

參加研究有什麼益處？

您將被提供免費的脈診和中醫體質診斷。如果您接受了免費的心電圖、定量超聲心動圖、動脈僵硬度和身體成分檢查，您將獲得相關報告。

如果出現錯誤？

我們不預期任何因参與本研究帶來的傷害。萬一因参與研究引致損傷，我們會提供醫療服務。沒有特別的補償安排。如果是由於疏忽而導致您受到損害，您可以有理由訴諸法律。無論如何，如果您想投訴在研究過程中得到的待遇，我們會提供有關健康服務投訴機制給您。

我參與本研究會被保密嗎?

作為研究對象，所有關於您的資料會絕對保密。您的姓名或您的個人身分不會被利用作任何公共用途、出版、或傳送到醫療中心之外。根據香港特別行政區執行法例，尤其是個人資料（隱私）條例，您享有或可享有權利保護保密個人資料，例如在這項研究中收集、保管、保存、管理、控制、使用（包括分析或比較）、進出香港的傳輸，不洩露、刪除或以任何方式處理或銷毀任何您的個人資料。 在本同意書上簽署參加，您明確授權給研究醫生、其研究小組和香港大學及醫管局港島西醫院聯網研究倫理委員會使用、保存您的個人資料，並將會如本同意書的目的和所述情況使用。如果您有任何疑問，您應該徵詢個人資料私隱專員或其辦公室（電話號碼：2255-4086），關於妥善監控或指導您對個人資料的保障，以確保您完全知悉及明白到遵守規管私隱資料法例的重要性。

完成項目後，志願者將獲發500港幣津貼以補償參加此項研究所付出之時間、所造成之不便及所需的交通費用或其他開支。

在以下簽署並註明日期，我同意：

1. 我在此證實，我已閱讀及明白上述研究同意書的內容，並獲得了提問的機會。
2. 我明白監管機構（香港大學及醫管局港島西醫院聯網研究倫理委員會）委派的人員可能會查核有關我參與此項研究的記錄。我允許他們可以獲得我的記錄。
3. 我同意參加以上研究項目。

參加者編號:

研究題目：中醫脈象的人工智能識別及其與體質類型的相關性研究

研究醫生：**沈劍剛教授**

汪陈羽	**汪陈羽**	**2024-07-26**
參加者簽署	參加者姓名	日期
[illegible] 陈小同	**陈小同**	**2024-07-26**
研究人員簽署	研究人員姓名	日期

知情同意書

研究名稱：中醫脈象的人工智能識別及其與體質類型的相關性研究

版本編號：Version 4.0　2023.08.17

研究地點：香港大學中醫藥學院

研究醫生：沈劍剛教授

我們邀請您參加一項研究。決定是否參與研究計劃前，請您務必明白進行這項研究的原因和程式。請仔細閱讀以下內容，如有任何疑問，簽署同意書之前請先向負責此項研究的中醫醫生或研究人員查詢。

關於此項研究

我們研究的目的是探究中醫脈象的人工智能識別及其與體質類型的相關性。

如果我參加將會做什麼?

參加者將按照身體情況分成兩組：

1.對照組：無持續3個月或以上的慢性疾病；目前沒有使用任何藥物。

2.病例組：經西醫診斷為心血管疾病、代謝綜合症、糖尿病、高血壓、高血脂症或肥胖。

您將分別接受基於人工智能的脈診儀檢測和有經驗中醫師的脈診檢查，並填寫中醫體質量表、中醫五臟健康量表和壓力知覺量表。根據研究方案，您可能被安排接受免費的心電圖、定量超聲心動圖、動脈僵硬度和身體成分檢查。

參加研究有什麼益處?

您將被提供免費的脈診和中醫體質診斷。如果您接受了免費的心電圖、定量超聲心動圖、動脈僵硬度和身體成分檢查，您將獲得相關報告。

如果出現錯誤?

我們不預期任何因參與本研究帶來的傷害。萬一因參與研究引致損傷，我們會提供醫療服務。沒有特別的補償安排。如果是由於疏忽而導致您受到損害，您可以有理由訴諸法律。無論如何，如果您想投訴在研究過程中得到的待遇，我們會提供有關健康服務投訴機制給您。

我參與本研究會被保密嗎?

作為研究對象，所有關於您的資料會絕對保密。您的姓名或您的個人身分不會被利用作任何公共用途、出版、或傳送到醫療中心之外。根據香港特別行政區執行法例，尤其是個人資料（隱私）條例，您享有或可享有權利保護保密個人資料，例如在這項研究中收集、保管、保存、管理、控制、使用（包括分析或比較）、進出香港的傳輸，不洩露、刪除或以任何方式處理或銷毀任何您的個人資料。 在本同意書上簽署參加，您明確授權給研究醫生、其研究小組和香港大學及醫管局港島西醫院聯網研究倫理委員會使用、保存您的個人資料，並將會如本同意書的目的和所述情況使用。如果您有任何疑問，您應該徵詢個人資料私隱專員或其辦公室（電話號碼：2255-4086），關於妥善監控或指導您對個人資料的保障，以確保您完全知悉及明白到遵守規管私隱資料法例的重要性。

完成項目後，志願者將獲發500港幣津貼以補償參加此項研究所付出之時間、所造成之不便及所需的交通費用或其他開支。

在以下簽署並註明日期，我同意：

1. 我在此證實，我已閱讀及明白上述研究同意書的內容，並獲得了提問的機會。
2. 我明白監管機構（香港大學及醫管局港島西醫院聯網研究倫理委員會）委派的人員可能會查核有關我參與此項研究的記錄。我允許他們可以獲得我的記錄。
3. 我同意參加以上研究項目。

參加者編號：

研究題目：中醫脈象的人工智能識別及其與體質類型的相關性研究

研究醫生：**沈劍剛教授**

汪青海	汪青海	2024-07-26
參加者簽署	參加者姓名	日期
[illegible] 陈小同	陈小同	2024-07-26
研究人員簽署	研究人員姓名	日期

知情同意書

研究名稱：中醫脈象的人工智能識別及其與體質類型的相關性研究

版本編號：Version 4.0　2023.08.17

研究地點：香港大學中醫藥學院

研究醫生：沈劍剛教授

我們邀請您參加一項研究。決定是否參與研究計劃前，請您務必明白進行這項研究的原因和程式。請仔細閱讀以下內容，如有任何疑問，簽署同意書之前請先向負責此項研究的中醫醫生或研究人員查詢。

關於此項研究

我們研究的目的是探究中醫脈象的人工智能識別及其與體質類型的相關性。

如果我參加將會做什麼？

參加者將按照身體情況分成兩組：

1.對照組：無持續3個月或以上的慢性疾病；目前没有使用任何藥物。

2.病例組：經西醫診斷為心血管疾病、代謝綜合症、糖尿病、高血壓、高血脂症或肥胖。

您將分別接受基於人工智能的脈診儀檢測和有經驗中醫師的脈診檢查，並填寫中醫體質量表、中醫五臟健康量表和壓力知覺量表。根據研究方案，您可能被安排接受免費的心電圖、定量超聲心動圖、動脈僵硬度和身體成分檢查。

參加研究有什麼益處？

您將被提供免費的脈診和中醫體質診斷。如果您接受了免費的心電圖、定量超聲心動圖、動脈僵硬度和身體成分檢查，您將獲得相關報告。

如果出現錯誤？

我們不預期任何因參與本研究帶來的傷害。萬一因參與研究引致損傷，我們會提供醫療服務。沒有特別的補償安排。如果是由於疏忽而導致您受到損害，您可以有理由訴諸法律。無論如何，如果您想投訴在研究過程中得到的待遇，我們會提供有關健康服務投訴機制給您。

我參與本研究會被保密嗎？

作為研究對象，所有關於您的資料會絕對保密。您的姓名或您的個人身分不會被利用作任何公共用途、出版、或傳送到醫療中心之外。根據香港特別行政區執行法例，尤其是個人資料（隱私）條例，您享有或可享有權利保護保密個人資料，例如在這項研究中收集、保管、保存、管理、控制、使用（包括分析或比較）、進出香港的傳輸，不洩露、刪除或以任何方式處理或銷毀任何您的個人資料。 在本同意書上簽署參加，您明確授權給研究醫生、其研究小組和香港大學及醫管局港島西醫院聯網研究倫理委員會使用、保存您的個人資料，並將會如本同意書的目的和所述情況使用。如果您有任何疑問，您應該徵詢個人資料私隱專員或其辦公室（電話號碼：2255-4086），關於妥善監控或指導您對個人資料的保障，以確保您完全知悉及明白到遵守規管私隱資料法例的重要性。

完成項目後，志願者將獲發500港幣津貼以補償參加此項研究所付出之時間、所造成之不便及所需的交通費用或其他開支。

在以下簽署並註明日期，我同意：

1. 我在此證實，我已閱讀及明白上述研究同意書的內容，並獲得了提問的機會。
2. 我明白監管機構（香港大學及醫管局港島西醫院聯網研究倫理委員會）委派的人員可能會查核有關我參與此項研究的記錄。我允許他們可以獲得我的記錄。
3. 我同意參加以上研究項目。

參加者編號：

研究題目：中醫脈象的人工智能識別及其與體質類型的相關性研究

研究醫生：**沈劍剛教授**

王一帆	**王一帆**	**2024-07-17**
參加者簽署	參加者姓名	日期
陈小同	**陈小同**	**2024-07-17**
研究人員簽署	研究人員姓名	日期

知情同意書

研究名稱：中醫脈象的人工智能識別及其與體質類型的相關性研究

版本編號：Version 4.0 2023.08.17

研究地點：香港大學中醫藥學院

研究醫生：沈劍剛教授

我們邀請您參加一項研究。決定是否參與研究計劃前，請您務必明白進行這項研究的原因和程式。請仔細閱讀以下內容，如有任何疑問，簽署同意書之前請先向負責此項研究的中醫醫生或研究人員查詢。

關於此項研究

我們研究的目的是探究中醫脈象的人工智能識別及其與體質類型的相關性。

如果我參加將會做什麼？

參加者將按照身體情況分成兩組：

1.對照組：無持續3個月或以上的慢性疾病；目前没有使用任何藥物。

2.病例組：經西醫診斷為心血管疾病、代謝綜合症、糖尿病、高血壓、高血脂症或肥胖。

您將分別接受基於人工智能的脈診儀檢測和有經驗中醫師的脈診檢查，並填寫中醫體質量表、中醫五臟健康量表和壓力知覺量表。根據研究方案，您可能被安排接受免費的心電圖、定量超聲心動圖、動脈僵硬度和身體成分檢查。

參加研究有什麼益處？

您將被提供免費的脈診和中醫體質診斷。如果您接受了免費的心電圖、定量超聲心動圖、動脈僵硬度和身體成分檢查，您將獲得相關報告。

如果出現錯誤？

我們不預期任何因參與本研究帶來的傷害。萬一因參與研究引致損傷，我們會提供醫療服務。沒有特別的補償安排。如果是由於疏忽而導致您受到損害，您可以有理由訴諸法律。無論如何，如果您想投訴在研究過程中得到的待遇，我們會提供有關健康服務投訴機制給您。

我參與本研究會被保密嗎？

作為研究對象，所有關於您的資料會絕對保密。您的姓名或您的個人身分不會被利用作任何公共用途、出版、或傳送到醫療中心之外。根據香港特別行政區執行法例，尤其是個人資料（隱私）條例，您享有或可享有權利保護保密個人資料，例如在這項研究中收集、保管、保存、管理、控制、使用（包括分析或比較）、進出香港的傳輸，不洩露、刪除或以任何方式處理或銷毀任何您的個人資料。 在本同意書上簽署參加，您明確授權給研究醫生、其研究小組和香港大學及醫管局港島西醫院聯網研究倫理委員會使用、保存您的個人資料，並將會如本同意書的目的和所述情況使用。如果您有任何疑問，您應該徵詢個人資料私隱專員或其辦公室（電話號碼：2255-4086），關於妥善監控或指導您對個人資料的保障，以確保您完全知悉及明白到遵守規管私隱資料法例的重要性。

完成項目後，志願者將獲發500港幣津貼以補償參加此項研究所付出之時間、所造成之不便及所需的交通費用或其他開支。

在以下簽署並註明日期，我同意：

1. 我在此證實，我已閱讀及明白上述研究同意書的內容，並獲得了提問的機會。
2. 我明白監管機構（香港大學及醫管局港島西醫院聯網研究倫理委員會）委派的人員可能會查核有關我參與此項研究的記錄。我允許他們可以獲得我的記錄。
3. 我同意參加以上研究項目。

參加者編號：

研究題目：中醫脈象的人工智能識別及其與體質類型的相關性研究

研究醫生：**沈劍剛教授**

舒焱火	**舒焱火**	**2024-07-26**
參加者簽署	參加者姓名	日期
陈小同	**陈小同**	**2024-07-26**
研究人員簽署	研究人員姓名	日期

知情同意書

研究名稱： 中醫脈象的人工智能識別及其與體質類型的相關性研究

版本編號： Version 4.0 2023.08.17

研究地點： 香港大學中醫藥學院

研究醫生： 沈劍剛教授

我們邀請您參加一項研究。決定是否參與研究計劃前，請您務必明白進行這項研究的原因和程式。請仔細閱讀以下內容，如有任何疑問，簽署同意書之前請先向負責此項研究的中醫醫生或研究人員查詢。

關於此項研究

我們研究的目的是探究中醫脈象的人工智能識別及其與體質類型的相關性。

如果我參加將會做什麼？

參加者將按照身體情況分成兩組：

1.對照組：無持續3個月或以上的慢性疾病；目前没有使用任何藥物。

2.病例組：經西醫診斷為心血管疾病、代謝綜合症、糖尿病、高血壓、高血脂症或肥胖。

您將分別接受基於人工智能的脈診儀檢測和有經驗中醫師的脈診檢查，並填寫中醫體質量表、中醫五臟健康量表和壓力知覺量表。根據研究方案，您可能被安排接受免費的心電圖、定量超聲心動圖、動脈僵硬度和身體成分檢查。

參加研究有什麼益處？

您將被提供免費的脈診和中醫體質診斷。如果您接受了免費的心電圖、定量超聲心動圖、動脈僵硬度和身體成分檢查，您將獲得相關報告。

如果出現錯誤？

我們不預期任何因參與本研究帶來的傷害。萬一因參與研究引致損傷，我們會提供醫療服務。沒有特別的補償安排。如果是由於疏忽而導致您受到損害，您可以有理由訴諸法律。無論如何，如果您想投訴在研究過程中得到的待遇，我們會提供有關健康服務投訴機制給您。

我參與本研究會被保密嗎?

作為研究對象，所有關於您的資料會絕對保密。您的姓名或您的個人身分不會被利用作任何公共用途、出版、或傳送到醫療中心之外。根據香港特別行政區執行法例，尤其是個人資料（隱私）條例，您享有或可享有權利保護保密個人資料，例如在這項研究中收集、保管、保存、管理、控制、使用（包括分析或比較）、進出香港的傳輸，不洩露、刪除或以任何方式處理或銷毀任何您的個人資料。 在本同意書上簽署參加，您明確授權給研究醫生、其研究小組和香港大學及醫管局港島西醫院聯網研究倫理委員會使用、保存您的個人資料，並將會如本同意書的目的和所述情況使用。如果您有任何疑問，您應該徵詢個人資料私隱專員或其辦公室（電話號碼：2255-4086），關於妥善監控或指導您對個人資料的保障，以確保您完全知悉及明白到遵守規管私隱資料法例的重要性。

完成項目後，志願者將獲發500港幣津貼以補償參加此項研究所付出之時間、所造成之不便及所需的交通費用或其他開支。

在以下簽署並註明日期，我同意：

1. 我在此證實，我已閱讀及明白上述研究同意書的內容，並獲得了提問的機會。
2. 我明白監管機構（香港大學及醫管局港島西醫院聯網研究倫理委員會）委派的人員可能會查核有關我參與此項研究的記錄。我允許他們可以獲得我的記錄。
3. 我同意參加以上研究項目。

參加者編號：

研究題目：中醫脈象的人工智能識別及其與體質類型的相關性研究

研究醫生：**沈劍剛教授**

顧一飛	顧一飛	2024-07-12
參加者簽署	參加者姓名	日期
陳小同	陳小同	2024-07-12
研究人員簽署	研究人員姓名	日期

知情同意書

研究名稱：中醫脈象的人工智能識別及其與體質類型的相關性研究

版本編號：Version 4.0　2023.08.17

研究地點：香港大學中醫藥學院

研究醫生：沈劍剛教授

我們邀請您參加一項研究。決定是否參與研究計劃前，請您務必明白進行這項研究的原因和程式。請仔細閱讀以下內容，如有任何疑問，簽署同意書之前請先向負責此項研究的中醫醫生或研究人員查詢。

關於此項研究

我們研究的目的是探究中醫脈象的人工智能識別及其與體質類型的相關性。

如果我參加將會做什麼？

參加者將按照身體情況分成兩組：

1.對照組：無持續3個月或以上的慢性疾病；目前没有使用任何藥物。

2.病例組：經西醫診斷為心血管疾病、代謝綜合症、糖尿病、高血壓、高血脂症或肥胖。

您將分別接受基於人工智能的脈診儀檢測和有經驗中醫師的脈診檢查，並填寫中醫體質量表、中醫五臟健康量表和壓力知覺量表。根據研究方案，您可能被安排接受免費的心電圖、定量超聲心動圖、動脈僵硬度和身體成分檢查。

參加研究有什麼益處？

您將被提供免費的脈診和中醫體質診斷。如果您接受了免費的心電圖、定量超聲心動圖、動脈僵硬度和身體成分檢查，您將獲得相關報告。

如果出現錯誤？

我們不預期任何因參與本研究帶來的傷害。萬一因參與研究引致損傷，我們會提供醫療服務。沒有特別的補償安排。如果是由於疏忽而導致您受到損害，您可以有理由訴諸法律。無論如何，如果您想投訴在研究過程中得到的待遇，我們會提供有關健康服務投訴機制給您。

我參與本研究會被保密嗎?

作為研究對象，所有關於您的資料會絕對保密。您的姓名或您的個人身分不會被利用作任何公共用途、出版、或傳送到醫療中心之外。根據香港特別行政區執行法例，尤其是個人資料（隱私）條例，您享有或可享有權利保護保密個人資料，例如在這項研究中收集、保管、保存、管理、控制、使用（包括分析或比較）、進出香港的傳輸，不洩露、刪除或以任何方式處理或銷毀任何您的個人資料。 在本同意書上簽署參加，您明確授權給研究醫生、其研究小組和香港大學及醫管局港島西醫院聯網研究倫理委員會使用、保存您的個人資料，並將會如本同意書的目的和所述情況使用。如果您有任何疑問，您應該徵詢個人資料私隱專員或其辦公室（電話號碼：2255-4086），關於妥善監控或指導您對個人資料的保障，以確保您完全知悉及明白到遵守規管私隱資料法例的重要性。

完成項目後，志願者將獲發500港幣津貼以補償參加此項研究所付出之時間、所造成之不便及所需的交通費用或其他開支。

在以下簽署並註明日期，我同意：

1. 我在此證實，我已閱讀及明白上述研究同意書的內容，並獲得了提問的機會。
2. 我明白監管機構（香港大學及醫管局港島西醫院聯網研究倫理委員會）委派的人員可能會查核有關我參與此項研究的記錄。我允許他們可以獲得我的記錄。
3. 我同意參加以上研究項目。

參加者編號：

研究題目：中醫脈象的人工智能識別及其與體質類型的相關性研究

研究醫生：**沈劍剛教授**

參加者簽署	參加者姓名	日期 2024-07-16

研究人員簽署	研究人員姓名	日期 2024-07-16

知情同意書

研究名稱：中醫脈象的人工智能識別及其與體質類型的相關性研究

版本編號：Version 4.0 2023.08.17

研究地點：香港大學中醫藥學院

研究醫生：沈劍剛教授

我們邀請您參加一項研究。決定是否參與研究計劃前，請您務必明白進行這項研究的原因和程式。請仔細閱讀以下內容，如有任何疑問，簽署同意書之前請先向負責此項研究的中醫醫生或研究人員查詢。

關於此項研究

我們研究的目的是探究中醫脈象的人工智能識別及其與體質類型的相關性。

如果我參加將會做什麼？

參加者將按照身體情況分成兩組：

1.對照組：無持續3個月或以上的慢性疾病；目前没有使用任何藥物。

2.病例組：經西醫診斷為心血管疾病、代謝綜合症、糖尿病、高血壓、高血脂症或肥胖。

您將分別接受基於人工智能的脈診儀檢測和有經驗中醫師的脈診檢查，並填寫中醫體質量表、中醫五臟健康量表和壓力知覺量表。根據研究方案，您可能被安排接受免費的心電圖、定量超聲心動圖、動脈僵硬度和身體成分檢查。

參加研究有什麼益處？

您將被提供免費的脈診和中醫體質診斷。如果您接受了免費的心電圖、定量超聲心動圖、動脈僵硬度和身體成分檢查，您將獲得相關報告。

如果出現錯誤？

我們不預期任何因參與本研究帶來的傷害。萬一因參與研究引致損傷，我們會提供醫療服務。沒有特別的補償安排。如果是由於疏忽而導致您受到損害，您可以有理由訴諸法律。無論如何，如果您想投訴在研究過程中得到的待遇，我們會提供有關健康服務投訴機制給您。

我參與本研究會被保密嗎？

作為研究對象，所有關於您的資料會絕對保密。您的姓名或您的個人身分不會被利用作任何公共用途、出版、或傳送到醫療中心之外。根據香港特別行政區執行法例，尤其是個人資料（隱私）條例，您享有或可享有權利保護保密個人資料，例如在這項研究中收集、保管、保存、管理、控制、使用（包括分析或比較）、進出香港的傳輸，不洩露、刪除或以任何方式處理或銷毀任何您的個人資料。 在本同意書上簽署參加，您明確授權給研究醫生、其研究小組和香港大學及醫管局港島西醫院聯網研究倫理委員會使用、保存您的個人資料，並將會如本同意書的目的和所述情況使用。如果您有任何疑問，您應該徵詢個人資料私隱專員或其辦公室（電話號碼：2255-4086），關於妥善監控或指導您對個人資料的保障，以確保您完全知悉及明白到遵守規管私隱資料法例的重要性。

完成項目後，志願者將獲發500港幣津貼以補償參加此項研究所付出之時間、所造成之不便及所需的交通費用或其他開支。

在以下簽署並註明日期，我同意：

1. 我在此證實，我已閱讀及明白上述研究同意書的內容，並獲得了提問的機會。
2. 我明白監管機構（香港大學及醫管局港島西醫院聯網研究倫理委員會）委派的人員可能會查核有關我參與此項研究的記錄。我允許他們可以獲得我的記錄。
3. 我同意參加以上研究項目。

參加者編號:

研究題目：中醫脈象的人工智能識別及其與體質類型的相關性研究

研究醫生：**沈劍剛教授**

[illegible]	田晴婷	21/03/2024
參加者簽署	參加者姓名	日期
[illegible]	陳小同	21/03/2024
研究人員簽署	研究人員姓名	日期

知情同意書

研究名稱： 中醫脈象的人工智能識別及其與體質類型的相關性研究

版本編號： Version 4.0 2023.08.17

研究地點： 香港大學中醫藥學院

研究醫生： 沈劍剛教授

我們邀請您參加一項研究。決定是否參與研究計劃前，請您務必明白進行這項研究的原因和程式。請仔細閱讀以下內容，如有任何疑問，簽署同意書之前請先向負責此項研究的中醫醫生或研究人員查詢。

關於此項研究

我們研究的目的是探究中醫脈象的人工智能識別及其與體質類型的相關性。

如果我參加將會做什麼？

參加者將按照身體情況分成兩組：

1. 對照組：無持續3個月或以上的慢性疾病；目前沒有使用任何藥物。
2. 病例組：經西醫診斷為心血管疾病、代謝綜合症、糖尿病、高血壓、高血脂症或肥胖。

您將分別接受基於人工智能的脈診儀檢測和有經驗中醫師的脈診檢查，並填寫中醫體質量表、中醫五臟健康量表和壓力知覺量表。根據研究方案，您可能被安排接受免費的心電圖、定量超聲心動圖、動脈僵硬度和身體成分檢查。

參加研究有什麼益處？

您將被提供免費的脈診和中醫體質診斷。如果您接受了免費的心電圖、定量超聲心動圖、動脈僵硬度和身體成分檢查，您將獲得相關報告。

如果出現錯誤？

我們不預期任何因參與本研究帶來的傷害。萬一因參與研究引致損傷，我們會提供醫療服務。沒有特別的補償安排。如果是由於疏忽而導致您受到損害，您可以有理由訴諸法律。無論如何，如果您想投訴在研究過程中得到的待遇，我們會提供有關健康服務投訴機制給您。

我參與本研究會被保密嗎？

作為研究對象，所有關於您的資料會絕對保密。您的姓名或您的個人身分不會被利用作任何公共用途、出版、或傳送到醫療中心之外。根據香港特別行政區執行法例，尤其是個人資料（隱私）條例，您享有或可享有權利保護保密個人資料，例如在這項研究中收集、保管、保存、管理、控制、使用（包括分析或比較）、進出香港的傳輸，不洩露、刪除或以任何方式處理或銷毀任何您的個人資料。 在本同意書上簽署參加，您明確授權給研究醫生、其研究小組和香港大學及醫管局港島西醫院聯網研究倫理委員會使用、保存您的個人資料，並將會如本同意書的目的和所述情況使用。如果您有任何疑問，您應該徵詢個人資料私隱專員或其辦公室（電話號碼：2255-4086），關於妥善監控或指導您對個人資料的保障，以確保您完全知悉及明白到遵守規管私隱資料法例的重要性。

完成項目後，志願者將獲發500港幣津貼以補償參加此項研究所付出之時間、所造成之不便及所需的交通費用或其他開支。

在以下簽署並註明日期，我同意：

1. 我在此證實，我已閱讀及明白上述研究同意書的內容，並獲得了提問的機會。
2. 我明白監管機構（香港大學及醫管局港島西醫院聯網研究倫理委員會）委派的人員可能會查核有關我參與此項研究的記錄。我允許他們可以獲得我的記錄。
3. 我同意參加以上研究項目。

參加者編號：

研究題目：中醫脈象的人工智能識別及其與體質類型的相關性研究

研究醫生：**沈劍剛教授**

[signature]	周晴婷	21/03/2024
參加者簽署	參加者姓名	日期
[signature]	陳小同	21/03/2024
研究人員簽署	研究人員姓名	日期

知情同意書

研究名稱： 中醫脈象的人工智能識別及其與體質類型的相關性研究

版本編號：Version 4.0 2023.08.17

研究地點：香港大學中醫藥學院

研究醫生：沈劍剛教授

我們邀請您參加一項研究。決定是否參與研究計劃前，請您務必明白進行這項研究的原因和程式。請仔細閱讀以下內容，如有任何疑問，簽署同意書之前請先向負責此項研究的中醫醫生或研究人員查詢。

關於此項研究

我們研究的目的是探究中醫脈象的人工智能識別及其與體質類型的相關性。

如果我參加將會做什麼？

參加者將按照身體情況分成兩組：

1. 對照組：無持續3個月或以上的慢性疾病；目前没有使用任何藥物。

2. 病例組：經西醫診斷為心血管疾病、代謝綜合症、糖尿病、高血壓、高血脂症或肥胖。

您將分別接受基於人工智能的脈診儀檢測和有經驗中醫師的脈診檢查，並填寫中醫體質量表、中醫五臟健康量表和壓力知覺量表。根據研究方案，您可能被安排接受免費的心電圖、定量超聲心動圖、動脈僵硬度和身體成分檢查。

參加研究有什麼益處？

您將被提供免費的脈診和中醫體質診斷。如果您接受了免費的心電圖、定量超聲心動圖、動脈僵硬度和身體成分檢查，您將獲得相關報告。

如果出現錯誤？

我們不預期任何因參與本研究帶來的傷害。萬一因參與研究引致損傷，我們會提供醫療服務。沒有特別的補償安排。如果是由於疏忽而導致您受到損害，您可以有理由訴諸法律。無論如何，如果您想投訴在研究過程中得到的待遇，我們會提供有關健康服務投訴機制給您。

我參與本研究會被保密嗎？

作為研究對象，所有關於您的資料會絕對保密。您的姓名或您的個人身分不會被利用作任何公共用途、出版、或傳送到醫療中心之外。根據香港特別行政區執行法例，尤其是個人資料（隱私）條例，您享有或可享有權利保護保密個人資料，例如在這項研究中收集、保管、保存、管理、控制、使用（包括分析或比較）、進出香港的傳輸，不洩露、刪除或以任何方式處理或銷毀任何您的個人資料。 在本同意書上簽署參加，您明確授權給研究醫生、其研究小組和香港大學及醫管局港島西醫院聯網研究倫理委員會使用、保存您的個人資料，並將會如本同意書的目的和所述情況使用。如果您有任何疑問，您應該徵詢個人資料私隱專員或其辦公室（電話號碼：2255-4086），關於妥善監控或指導您對個人資料的保障，以確保您完全知悉及明白到遵守規管私隱資料法例的重要性。

完成項目後，志願者將獲發500港幣津貼以補償參加此項研究所付出之時間、所造成之不便及所需的交通費用或其他開支。

在以下簽署並註明日期，我同意：

1. 我在此證實，我已閱讀及明白上述研究同意書的內容，並獲得了提問的機會。
2. 我明白監管機構（香港大學及醫管局港島西醫院聯網研究倫理委員會）委派的人員可能會查核有關我參與此項研究的記錄。我允許他們可以獲得我的記錄。
3. 我同意參加以上研究項目。

參加者編號：

研究題目：中醫脈象的人工智能識別及其與體質類型的相關性研究

研究醫生：**沈劍剛教授**

參加者簽署	參加者姓名	2024-07-<br>16<br>日期
研究人員簽署	研究人員姓名	2024-07-<br>16<br>日期

知情同意書

研究名稱：中醫脈象的人工智能識別及其與體質類型的相關性研究

版本編號：Version 4.0　2023.08.17

研究地點：香港大學中醫藥學院

研究醫生：沈劍剛教授

我們邀請您參加一項研究。決定是否參與研究計劃前，請您務必明白進行這項研究的原因和程式。請仔細閱讀以下內容，如有任何疑問，簽署同意書之前請先向負責此項研究的中醫醫生或研究人員查詢。

關於此項研究

我們研究的目的是探究中醫脈象的人工智能識別及其與體質類型的相關性。

如果我參加將會做什麼？

參加者將按照身體情況分成兩組：

1. 對照組：無持續3個月或以上的慢性疾病；目前没有使用任何藥物。
2. 病例組：經西醫診斷為心血管疾病、代謝綜合症、糖尿病、高血壓、高血脂症或肥胖。

您將分別接受基於人工智能的脈診儀檢測和有經驗中醫師的脈診檢查，並填寫中醫體質量表、中醫五臟健康量表和壓力知覺量表。根據研究方案，您可能被安排接受免費的心電圖、定量超聲心動圖、動脈僵硬度和身體成分檢查。

參加研究有什麼益處？

您將被提供免費的脈診和中醫體質診斷。如果您接受了免費的心電圖、定量超聲心動圖、動脈僵硬度和身體成分檢查，您將獲得相關報告。

如果出現錯誤？

我們不預期任何因參與本研究帶來的傷害。萬一因參與研究引致損傷，我們會提供醫療服務。沒有特別的補償安排。如果是由於疏忽而導致您受到損害，您可以有理由訴諸法律。無論如何，如果您想投訴在研究過程中得到的待遇，我們會提供有關健康服務投訴機制給您。

我參與本研究會被保密嗎?

作為研究對象，所有關於您的資料會絕對保密。您的姓名或您的個人身分不會被利用作任何公共用途、出版、或傳送到醫療中心之外。根據香港特別行政區執行法例，尤其是個人資料（隱私）條例，您享有或可享有權利保護保密個人資料，例如在這項研究中收集、保管、保存、管理、控制、使用（包括分析或比較）、進出香港的傳輸，不洩露、刪除或以任何方式處理或銷毀任何您的個人資料。 在本同意書上簽署參加，您明確授權給研究醫生、其研究小組和香港大學及醫管局港島西醫院聯網研究倫理委員會使用、保存您的個人資料，並將會如本同意書的目的和所述情況使用。如果您有任何疑問，您應該徵詢個人資料私隱專員或其辦公室（電話號碼：2255-4086），關於妥善監控或指導您對個人資料的保障，以確保您完全知悉及明白到遵守規管私隱資料法例的重要性。

完成項目後，志願者將獲發500港幣津貼以補償參加此項研究所付出之時間、所造成之不便及所需的交通費用或其他開支。

在以下簽署並註明日期，我同意：

1. 我在此證實，我已閱讀及明白上述研究同意書的內容，並獲得了提問的機會。
2. 我明白監管機構（香港大學及醫管局港島西醫院聯網研究倫理委員會）委派的人員可能會查核有關我參與此項研究的記錄。我允許他們可以獲得我的記錄。
3. 我同意參加以上研究項目。

參加者編號：

研究題目：中醫脈象的人工智能識別及其與體質類型的相關性研究

研究醫生：**沈劍剛教授**

王玉华	王玉华	2024-07-26
參加者簽署	參加者姓名	日期
陈小同	陈小同	2024-07-26
研究人員簽署	研究人員姓名	日期

知情同意書

研究名稱：中醫脈象的人工智能識別及其與體質類型的相關性研究

版本編號：Version 4.0　2023.08.17

研究地點：香港大學中醫藥學院

研究醫生：沈劍剛教授

我們邀請您參加一項研究。決定是否參與研究計劃前，請您務必明白進行這項研究的原因和程式。請仔細閱讀以下內容，如有任何疑問，簽署同意書之前請先向負責此項研究的中醫醫生或研究人員查詢。

關於此項研究

我們研究的目的是探究中醫脈象的人工智能識別及其與體質類型的相關性。

如果我參加將會做什麼？

參加者將按照身體情況分成兩組：

1. 對照組：無持續3個月或以上的慢性疾病；目前没有使用任何藥物。

2. 病例組：經西醫診斷為心血管疾病、代謝綜合症、糖尿病、高血壓、高血脂症或肥胖。

您將分別接受基於人工智能的脈診儀檢測和有經驗中醫師的脈診檢查，並填寫中醫體質量表、中醫五臟健康量表和壓力知覺量表。根據研究方案，您可能被安排接受免費的心電圖、定量超聲心動圖、動脈僵硬度和身體成分檢查。

參加研究有什麼益處？

您將被提供免費的脈診和中醫體質診斷。如果您接受了免費的心電圖、定量超聲心動圖、動脈僵硬度和身體成分檢查，您將獲得相關報告。

如果出現錯誤？

我們不預期任何因參與本研究帶來的傷害。萬一因參與研究引致損傷，我們會提供醫療服務。沒有特別的補償安排。如果是由於疏忽而導致您受到損害，您可以有理由訴諸法律。無論如何，如果您想投訴在研究過程中得到的待遇，我們會提供有關健康服務投訴機制給您。

我參與本研究會被保密嗎？

作為研究對象，所有關於您的資料會絕對保密。您的姓名或您的個人身分不會被利用作任何公共用途、出版、或傳送到醫療中心之外。根據香港特別行政區執行法例，尤其是個人資料（隱私）條例，您享有或可享有權利保護保密個人資料，例如在這項研究中收集、保管、保存、管理、控制、使用（包括分析或比較）、進出香港的傳輸，不洩露、刪除或以任何方式處理或銷毀任何您的個人資料。 在本同意書上簽署參加，您明確授權給研究醫生、其研究小組和香港大學及醫管局港島西醫院聯網研究倫理委員會使用、保存您的個人資料，並將會如本同意書的目的和所述情況使用。如果您有任何疑問，您應該徵詢個人資料私隱專員或其辦公室（電話號碼：2255-4086），關於妥善監控或指導您對個人資料的保障，以確保您完全知悉及明白到遵守規管私隱資料法例的重要性。

完成項目後，志願者將獲發500港幣津貼以補償參加此項研究所付出之時間、所造成之不便及所需的交通費用或其他開支。

在以下簽署並註明日期，我同意：

1. 我在此證實，我已閱讀及明白上述研究同意書的內容，並獲得了提問的機會。
2. 我明白監管機構（香港大學及醫管局港島西醫院聯網研究倫理委員會）委派的人員可能會查核有關我參與此項研究的記錄。我允許他們可以獲得我的記錄。
3. 我同意參加以上研究項目。

參加者編號:

研究題目：中醫脈象的人工智能識別及其與體質類型的相關性研究

研究醫生：**沈劍剛教授**

陈馨铭 (signature)	陈馨铭	2024-07-26
參加者簽署	參加者姓名	日期
陈小同 (signature)	陈小同	2024-07-26
研究人員簽署	研究人員姓名	日期

知情同意書

研究名稱：中醫脈象的人工智能識別及其與體質類型的相關性研究

版本編號：Version 4.0 2023.08.17

研究地點：香港大學中醫藥學院

研究醫生：沈劍剛教授

我們邀請您參加一項研究。決定是否參與研究計劃前，請您務必明白進行這項研究的原因和程式。請仔細閱讀以下內容，如有任何疑問，簽署同意書之前請先向負責此項研究的中醫醫生或研究人員查詢。

關於此項研究

我們研究的目的是探究中醫脈象的人工智能識別及其與體質類型的相關性。

如果我參加將會做什麼？

參加者將按照身體情況分成兩組：

1. 對照組：無持續3個月或以上的慢性疾病；目前没有使用任何藥物。

2. 病例組：經西醫診斷為心血管疾病、代謝綜合症、糖尿病、高血壓、高血脂症或肥胖。

您將分別接受基於人工智能的脈診儀檢測和有經驗中醫師的脈診檢查，並填寫中醫體質量表、中醫五臟健康量表和壓力知覺量表。根據研究方案，您可能被安排接受免費的心電圖、定量超聲心動圖、動脈僵硬度和身體成分檢查。

參加研究有什麼益處？

您將被提供免費的脈診和中醫體質診斷。如果您接受了免費的心電圖、定量超聲心動圖、動脈僵硬度和身體成分檢查，您將獲得相關報告。

如果出現錯誤？

我們不預期任何因參與本研究帶來的傷害。萬一因參與研究引致損傷，我們會提供醫療服務。沒有特別的補償安排。如果是由於疏忽而導致您受到損害，您可以有理由訴諸法律。無論如何，如果您想投訴在研究過程中得到的待遇，我們會提供有關健康服務投訴機制給您。

我參與本研究會被保密嗎？

作為研究對象，所有關於您的資料會絕對保密。您的姓名或您的個人身分不會被利用作任何公共用途、出版、或傳送到醫療中心之外。根據香港特別行政區執行法例，尤其是個人資料（隱私）條例，您享有或可享有權利保護保密個人資料，例如在這項研究中收集、保管、保存、管理、控制、使用（包括分析或比較）、進出香港的傳輸，不洩露、刪除或以任何方式處理或銷毀任何您的個人資料。 在本同意書上簽署參加，您明確授權給研究醫生、其研究小組和香港大學及醫管局港島西醫院聯網研究倫理委員會使用、保存您的個人資料，並將會如本同意書的目的和所述情況使用。如果您有任何疑問，您應該徵詢個人資料私隱專員或其辦公室（電話號碼：2255-4086），關於妥善監控或指導您對個人資料的保障，以確保您完全知悉及明白到遵守規管私隱資料法例的重要性。

完成項目後，志願者將獲發500港幣津貼以補償參加此項研究所付出之時間、所造成之不便及所需的交通費用或其他開支。

在以下簽署並註明日期，我同意：

1. 我在此證實，我已閱讀及明白上述研究同意書的內容，並獲得了提問的機會。
2. 我明白監管機構（香港大學及醫管局港島西醫院聯網研究倫理委員會）委派的人員可能會查核有關我參與此項研究的記錄。我允許他們可以獲得我的記錄。
3. 我同意參加以上研究項目。

參加者編號：

研究題目：中醫脈象的人工智能識別及其與體質類型的相關性研究

研究醫生：**沈劍剛教授**

林穎怡	林穎怡.	18-07-2024
參加者簽署	參加者姓名	日期
[signature] 陈小同	陈小同	18-07-2024
研究人員簽署	研究人員姓名	日期

知情同意書

研究名稱： 中醫脈象的人工智能識別及其與體質類型的相關性研究

版本編號： Version 4.0　2023.08.17

研究地點： 香港大學中醫藥學院

研究醫生： 沈劍剛教授

我們邀請您參加一項研究。決定是否參與研究計劃前，請您務必明白進行這項研究的原因和程式。請仔細閱讀以下內容，如有任何疑問，簽署同意書之前請先向負責此項研究的中醫醫生或研究人員查詢。

關於此項研究

我們研究的目的是探究中醫脈象的人工智能識別及其與體質類型的相關性。

如果我參加將會做什麼？

參加者將按照身體情況分成兩組：

1. 對照組：無持續3個月或以上的慢性疾病；目前没有使用任何藥物。
2. 病例組：經西醫診斷為心血管疾病、代謝綜合症、糖尿病、高血壓、高血脂症或肥胖。

您將分別接受基於人工智能的脈診儀檢測和有經驗中醫師的脈診檢查，並填寫中醫體質量表、中醫五臟健康量表和壓力知覺量表。根據研究方案，您可能被安排接受免費的心電圖、定量超聲心動圖、動脈僵硬度和身體成分檢查。

參加研究有什麼益處？

您將被提供免費的脈診和中醫體質診斷。如果您接受了免費的心電圖、定量超聲心動圖、動脈僵硬度和身體成分檢查，您將獲得相關報告。

如果出現錯誤？

我們不預期任何因參與本研究帶來的傷害。萬一因參與研究引致損傷，我們會提供醫療服務。沒有特別的補償安排。如果是由於疏忽而導致您受到損害，您可以有理由訴諸法律。無論如何，如果您想投訴在研究過程中得到的待遇，我們會提供有關健康服務投訴機制給您。

我參與本研究會被保密嗎？

作為研究對象，所有關於您的資料會絕對保密。您的姓名或您的個人身分不會被利用作任何公共用途、出版、或傳送到醫療中心之外。根據香港特別行政區執行法例，尤其是個人資料（隱私）條例，您享有或可享有權利保護保密個人資料，例如在這項研究中收集、保管、保存、管理、控制、使用（包括分析或比較）、進出香港的傳輸，不洩露、刪除或以任何方式處理或銷毀任何您的個人資料。 在本同意書上簽署參加，您明確授權給研究醫生、其研究小組和香港大學及醫管局港島西醫院聯網研究倫理委員會使用、保存您的個人資料，並將會如本同意書的目的和所述情況使用。如果您有任何疑問，您應該徵詢個人資料私隱專員或其辦公室（電話號碼：2255-4086），關於妥善監控或指導您對個人資料的保障，以確保您完全知悉及明白到遵守規管私隱資料法例的重要性。

完成項目後，志願者將獲發500港幣津貼以補償參加此項研究所付出之時間、所造成之不便及所需的交通費用或其他開支。

在以下簽署並註明日期，我同意：

1. 我在此證實，我已閱讀及明白上述研究同意書的內容，並獲得了提問的機會。
2. 我明白監管機構（香港大學及醫管局港島西醫院聯網研究倫理委員會）委派的人員可能會查核有關我參與此項研究的記錄。我允許他們可以獲得我的記錄。
3. 我同意參加以上研究項目。

參加者編號：

研究題目：中醫脈象的人工智能識別及其與體質類型的相關性研究

研究醫生：**沈劍剛教授**

HOU Saolin	侯[illegible]	2024.01.25
參加者簽署	參加者姓名	日期
陈小同	陈小同	18-07-2024
研究人員簽署	研究人員姓名	日期

知情同意書

研究名稱： 中醫脈象的人工智能識別及其與體質類型的相關性研究

版本編號：Version 4.0 2023.08.17

研究地點：香港大學中醫藥學院

研究醫生：沈劍剛教授

我們邀請您參加一項研究。決定是否參與研究計劃前，請您務必明白進行這項研究的原因和程式。請仔細閱讀以下內容，如有任何疑問，簽署同意書之前請先向負責此項研究的中醫醫生或研究人員查詢。

關於此項研究

我們研究的目的是探究中醫脈象的人工智能識別及其與體質類型的相關性。

如果我參加將會做什麼？

參加者將按照身體情況分成兩組：

1. 對照組：無持續3個月或以上的慢性疾病；目前没有使用任何藥物。

2. 病例組：經西醫診斷為心血管疾病、代謝綜合症、糖尿病、高血壓、高血脂症或肥胖。

您將分別接受基於人工智能的脈診儀檢測和有經驗中醫師的脈診檢查，並填寫中醫體質量表、中醫五臟健康量表和壓力知覺量表。根據研究方案，您可能被安排接受免費的心電圖、定量超聲心動圖、動脈僵硬度和身體成分檢查。

參加研究有什麼益處？

您將被提供免費的脈診和中醫體質診斷。如果您接受了免費的心電圖、定量超聲心動圖、動脈僵硬度和身體成分檢查，您將獲得相關報告。

如果出現錯誤？

我們不預期任何因參與本研究帶來的傷害。萬一因參與研究引致損傷，我們會提供醫療服務。沒有特別的補償安排。如果是由於疏忽而導致您受到損害，您可以有理由訴諸法律。無論如何，如果您想投訴在研究過程中得到的待遇，我們會提供有關健康服務投訴機制給您。

我參與本研究會被保密嗎？

作為研究對象，所有關於您的資料會絕對保密。您的姓名或您的個人身分不會被利用作任何公共用途、出版、或傳送到醫療中心之外。根據香港特別行政區執行法例，尤其是個人資料（隱私）條例，您享有或可享有權利保護保密個人資料，例如在這項研究中收集、保管、保存、管理、控制、使用（包括分析或比較）、進出香港的傳輸，不洩露、刪除或以任何方式處理或銷毀任何您的個人資料。 在本同意書上簽署參加，您明確授權給研究醫生、其研究小組和香港大學及醫管局港島西醫院聯網研究倫理委員會使用、保存您的個人資料，並將會如本同意書的目的和所述情況使用。如果您有任何疑問，您應該徵詢個人資料私隱專員或其辦公室（電話號碼：2255-4086），關於妥善監控或指導您對個人資料的保障，以確保您完全知悉及明白到遵守規管私隱資料法例的重要性。

完成項目後，志願者將獲發500港幣津貼以補償參加此項研究所付出之時間、所造成之不便及所需的交通費用或其他開支。

在以下簽署並註明日期，我同意：

1. 我在此證實，我已閱讀及明白上述研究同意書的內容，並獲得了提問的機會。
2. 我明白監管機構（香港大學及醫管局港島西醫院聯網研究倫理委員會）委派的人員可能會查核有關我參與此項研究的記錄。我允許他們可以獲得我的記錄。
3. 我同意參加以上研究項目。

參加者編號：

研究題目：中醫脈象的人工智能識別及其與體質類型的相關性研究

研究醫生：**沈劍剛教授**

[illegible]	祝竹兰	2024, 04, 04
參加者簽署	參加者姓名	日期
[illegible]	陳小同	2024-04-04
研究人員簽署	研究人員姓名	日期

知情同意書

研究名稱：中醫脈象的人工智能識別及其與體質類型的相關性研究

版本編號：Version 4.0 2023.08.17

研究地點：香港大學中醫藥學院

研究醫生：沈劍剛教授

我們邀請您參加一項研究。決定是否參與研究計劃前，請您務必明白進行這項研究的原因和程式。請仔細閱讀以下內容，如有任何疑問，簽署同意書之前請先向負責此項研究的中醫醫生或研究人員查詢。

關於此項研究

我們研究的目的是探究中醫脈象的人工智能識別及其與體質類型的相關性。

如果我參加將會做什麼？

參加者將按照身體情況分成兩組：

1. 對照組：無持續3個月或以上的慢性疾病；目前没有使用任何藥物。

2. 病例組：經西醫診斷為心血管疾病、代謝綜合症、糖尿病、高血壓、高血脂症或肥胖。

您將分別接受基於人工智能的脈診儀檢測和有經驗中醫師的脈診檢查，並填寫中醫體質量表、中醫五臟健康量表和壓力知覺量表。根據研究方案，您可能被安排接受免費的心電圖、定量超聲心動圖、動脈僵硬度和身體成分檢查。

參加研究有什麼益處？

您將被提供免費的脈診和中醫體質診斷。如果您接受了免費的心電圖、定量超聲心動圖、動脈僵硬度和身體成分檢查，您將獲得相關報告。

如果出現錯誤？

我們不預期任何因參與本研究帶來的傷害。萬一因參與研究引致損傷，我們會提供醫療服務。沒有特別的補償安排。如果是由於疏忽而導致您受到損害，您可以有理由訴諸法律。無論如何，如果您想投訴在研究過程中得到的待遇，我們會提供有關健康服務投訴機制給您。

我參與本研究會被保密嗎？

作為研究對象，所有關於您的資料會絕對保密。您的姓名或您的個人身分不會被利用作任何公共用途、出版、或傳送到醫療中心之外。根據香港特別行政區執行法例，尤其是個人資料（隱私）條例，您享有或可享有權利保護保密個人資料，例如在這項研究中收集、保管、保存、管理、控制、使用（包括分析或比較）、進出香港的傳輸，不洩露、刪除或以任何方式處理或銷毀任何您的個人資料。 在本同意書上簽署參加，您明確授權給研究醫生、其研究小組和香港大學及醫管局港島西醫院聯網研究倫理委員會使用、保存您的個人資料，並將會如本同意書的目的和所述情況使用。如果您有任何疑問，您應該徵詢個人資料私隱專員或其辦公室（電話號碼：2255-4086），關於妥善監控或指導您對個人資料的保障，以確保您完全知悉及明白到遵守規管私隱資料法例的重要性。

完成項目後，志願者將獲發500港幣津貼以補償參加此項研究所付出之時間、所造成之不便及所需的交通費用或其他開支。

在以下簽署並註明日期，我同意：

1. 我在此證實，我已閱讀及明白上述研究同意書的內容，並獲得了提問的機會。
2. 我明白監管機構（香港大學及醫管局港島西醫院聯網研究倫理委員會）委派的人員可能會查核有關我參與此項研究的記錄。我允許他們可以獲得我的記錄。
3. 我同意參加以上研究項目。

參加者編號：

研究題目：中醫脈象的人工智能識別及其與體質類型的相關性研究

研究醫生：**沈劍剛教授**

	潘美[illegible]	18/7/2024
參加者簽署	參加者姓名	日期
	田嬌嬌	18/07/2024
研究人員簽署	研究人員姓名	日期

知情同意書

研究名稱： 中醫脈象的人工智能識別及其與體質類型的相關性研究

版本編號：Version 4.0　2023.08.17

研究地點：香港大學中醫藥學院

研究醫生：沈劍剛教授

我們邀請您參加一項研究。決定是否參與研究計劃前，請您務必明白進行這項研究的原因和程式。請仔細閱讀以下內容，如有任何疑問，簽署同意書之前請先向負責此項研究的中醫醫生或研究人員查詢。

關於此項研究

我們研究的目的是探究中醫脈象的人工智能識別及其與體質類型的相關性。

如果我參加將會做什麼？

參加者將按照身體情況分成兩組：

1. 對照組：無持續3個月或以上的慢性疾病；目前沒有使用任何藥物。

2. 病例組：經西醫診斷為心血管疾病、代謝綜合症、糖尿病、高血壓、高血脂症或肥胖。

您將分別接受基於人工智能的脈診儀檢測和有經驗中醫師的脈診檢查，並填寫中醫體質量表、中醫五臟健康量表和壓力知覺量表。根據研究方案，您可能被安排接受免費的心電圖、定量超聲心動圖、動脈僵硬度和身體成分檢查。

參加研究有什麼益處？

您將被提供免費的脈診和中醫體質診斷。如果您接受了免費的心電圖、定量超聲心動圖、動脈僵硬度和身體成分檢查，您將獲得相關報告。

如果出現錯誤？

我們不預期任何因參與本研究帶來的傷害。萬一因參與研究引致損傷，我們會提供醫療服務。沒有特別的補償安排。如果是由於疏忽而導致您受到損害，您可以有理由訴諸法律。無論如何，如果您想投訴在研究過程中得到的待遇，我們會提供有關健康服務投訴機制給您。

我參與本研究會被保密嗎？

作為研究對象，所有關於您的資料會絕對保密。您的姓名或您的個人身分不會被利用作任何公共用途、出版、或傳送到醫療中心之外。根據香港特別行政區執行法例，尤其是個人資料（隱私）條例，您享有或可享有權利保護保密個人資料，例如在這項研究中收集、保管、保存、管理、控制、使用（包括分析或比較）、進出香港的傳輸，不洩露、刪除或以任何方式處理或銷毀任何您的個人資料。 在本同意書上簽署參加，您明確授權給研究醫生、其研究小組和香港大學及醫管局港島西醫院聯網研究倫理委員會使用、保存您的個人資料，並將會如本同意書的目的和所述情況使用。如果您有任何疑問，您應該徵詢個人資料私隱專員或其辦公室（電話號碼：2255-4086），關於妥善監控或指導您對個人資料的保障，以確保您完全知悉及明白到遵守規管私隱資料法例的重要性。

完成項目後，志願者將獲發500港幣津貼以補償參加此項研究所付出之時間、所造成之不便及所需的交通費用或其他開支。

在以下簽署並註明日期，我同意：

1. 我在此證實，我已閱讀及明白上述研究同意書的內容，並獲得了提問的機會。
2. 我明白監管機構（香港大學及醫管局港島西醫院聯網研究倫理委員會）委派的人員可能會查核有關我參與此項研究的記錄。我允許他們可以獲得我的記錄。
3. 我同意參加以上研究項目。

參加者編號：

研究題目：中醫脈象的人工智能識別及其與體質類型的相關性研究

研究醫生：**沈劍剛教授**

[signature]	Du Qiaohui	2024-06-27
參加者簽署	參加者姓名	日期
[signature]	田嬌[illegible]	2024-06-27
研究人員簽署	研究人員姓名	日期
